# Biomass Pre-Extraction as a Versatile Strategy to Improve Biorefinery Feedstock Flexibility, Sugar Yields, and Lignin Purity

**DOI:** 10.1021/acssuschemeng.2c00838

**Published:** 2022-04-27

**Authors:** Arjan T. Smit, André van Zomeren, Karla Dussan, Luke A. Riddell, Wouter J. J. Huijgen, Jan Wilco Dijkstra, Pieter C. A. Bruijnincx

**Affiliations:** † Unit Energy Transition, Biobased & Circular Technologies Group, The Netherlands Organisation for Applied Scientific Research (TNO), P.O. Box 1, 1755 ZG Petten, The Netherlands; ‡ Organic Chemistry and Catalysis, Debye Institute for Nanomaterials Science, Utrecht University, Universiteitsweg 99, 3584 CG Utrecht, The Netherlands

**Keywords:** sustainable feedstocks, biorefinery, cascading valorization, extractives, organosolv, lignin purity

## Abstract

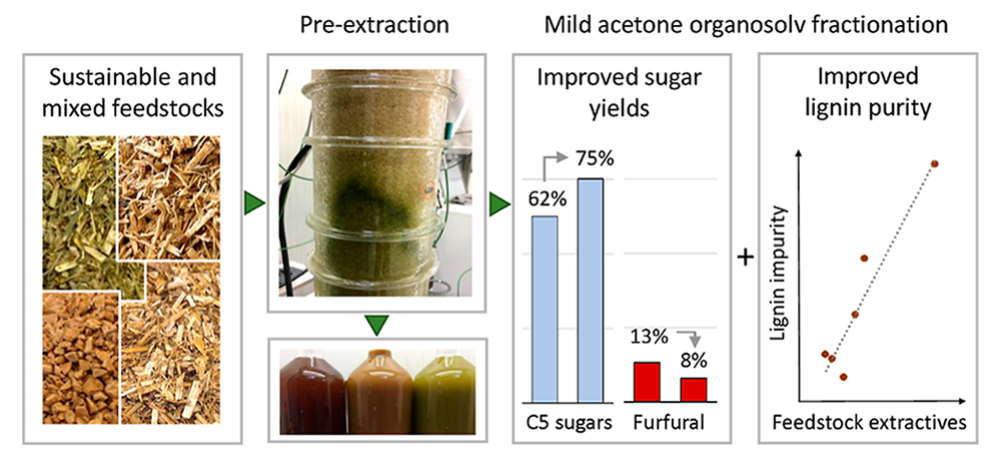

Feedstock flexibility is highly advantageous for the viability of (solvent-based) biorefineries but comes with the considerable challenge of having to cope with the varying nature and typically high abundance of nonlignocellulose compounds in the most readily available residual biomass streams. Here, we demonstrate that mild aqueous acetone organosolv fractionation of various complex lignocellulosic raw materials (roadside grass, wheat straw, birch branches, almond shells, and a mixed stream thereof) is indeed negatively affected by these compounds and present a versatile strategy to mitigate this bottleneck in biorefining. A biomass pre-extraction approach has been developed to remove the detrimental extractives with (aqueous) acetone prior to fractionation. Pre-extraction removed organic extractives as well as minerals, primarily reducing acid dose requirements for fractionation and loss of hemicellulose sugars by degradation and improved the purity of the isolated lignin. We show how pre-extraction affects the effectiveness of the biorefinery process, including detailed mass balances for pretreatment, downstream processing, and product characteristics, and how it affects solvent and energy use with a first conceptual process design. The integrated biorefining approach allows for the improved compatibility of biorefineries with sustainable feedstock supply chains, enhanced biomass valorization (i.e., isolation of bioactive compounds from the extract), and more effective biomass processing with limited variation in product quality.

## Abstract 2

Lignocellulose biorefining with improved feedstock flexibility, product purity, and enhanced biomass valorization by adopting an integrated extraction and fractionation approach.

## Introduction

The development of biorefineries converting biomass into sustainable energy carriers, chemicals, and materials is of paramount importance for the transition from a fossil-based society to a (circular) biobased one. Lignocellulosic biomass suitable for biorefining can come from agricultural residues, forestry residues, food-processing residues, dedicated energy crops, or biomass from other origins such as cattle manure and biomass from roadside verges. Biomass availability from a variety of sources in the European Union can more than match the needs of the biobased industry.^1−3^ Nevertheless, a major challenge in establishing viable biorefinery operations is to build sustainable and cost-effective value chains. This involves integration of feedstock supply with biorefinery processing and downstream applications and requires efficient logistics for biomass collection, densification, storage, and transportation.^2,4^ Environmental aspects must also be considered (land use, conservation of biodiversity, and soil quality).^5−7^ Biorefineries that operate on a single feedstock only, such as wood chips, may face challenges related to biomass availability and prices when demand for such feedstocks increases during the transition to a biobased economy. Indeed, maximum valorization of a wide variety of residual streams would be highly beneficial for the overall sustainability and viability of a biorefinery process. This requires technology to flexibly process multiple or mixed lignocellulosic streams to cope with variability in feedstock composition and biomass availability.

Most agricultural, food-processing, and forestry residues (including bark), as well as energy crops contain a significant amount of nonlignocellulose compounds. These compounds can be divided in two classes depending on their polarity and solubility. Hydrophilic extractives are soluble in water and/or polar solvents and mainly consist of a mix of (oligomeric) sugars, alditols, uronic acids, organic acids, proteins, pigments, terpenes, terpenoids, (in)organic salts, and so forth.^8,9^ Lipophilic extractives are soluble in more apolar solvents and include fatty and resin acids, fatty acid esters (e.g., steryl esters, waxes, and triglycerides), fatty alcohols, sterols, sterol glycosides, lipids, and oils.^10^ These extractives affect biorefinery processing and product yield/purity in different ways. For example, inorganic salts and especially chlorides in an acidic environment accelerate corrosion of the processing equipment by enhanced stress corrosion cracking, pitting, and destabilization of the steel surface film.^11−13^ In addition, inorganic salts can accelerate degradation of oligomeric and monomeric sugars during pretreatment.^14,15^ Besides loss of sugars, the sugar derivatives as well as nonlignocellulose compounds can potentially affect lignin purity by formation of polyphenolic moieties, commonly referred to as pseudo-lignin.^16−18^ Lignin purity can also be affected by reaction/condensation with nonlignocellulose compounds or by coprecipitation of extractives with lignin during solvent recovery/lignin precipitation. All these negatively affect the economic viability of a biorefinery process, for which high-value lignin applications are key.

Biomass extractive removal by pre-extraction prior to fractionation can minimize the aforementioned effects. Furthermore, pre-extraction offers the opportunity to isolate extractives to further maximize biomass valorization in new application outlets, such as flavors, vitamins, organic acids, antioxidants, antimicrobial agents, flavonoids, waxes, biopolymers, and so forth. Many examples are available for optimized processes for the extraction and isolation of biomass extractives.^19−22^ However, these studies primarily focus on the stand-alone valorization of extractives but do not integrate the extraction process with subsequent biorefining of the extracted solids. Combining biomass pre-extraction and fractionation in an integrated cascading process can both maximize biomass utilization and increase feedstock flexibility for biorefineries.

**Figure 1:**
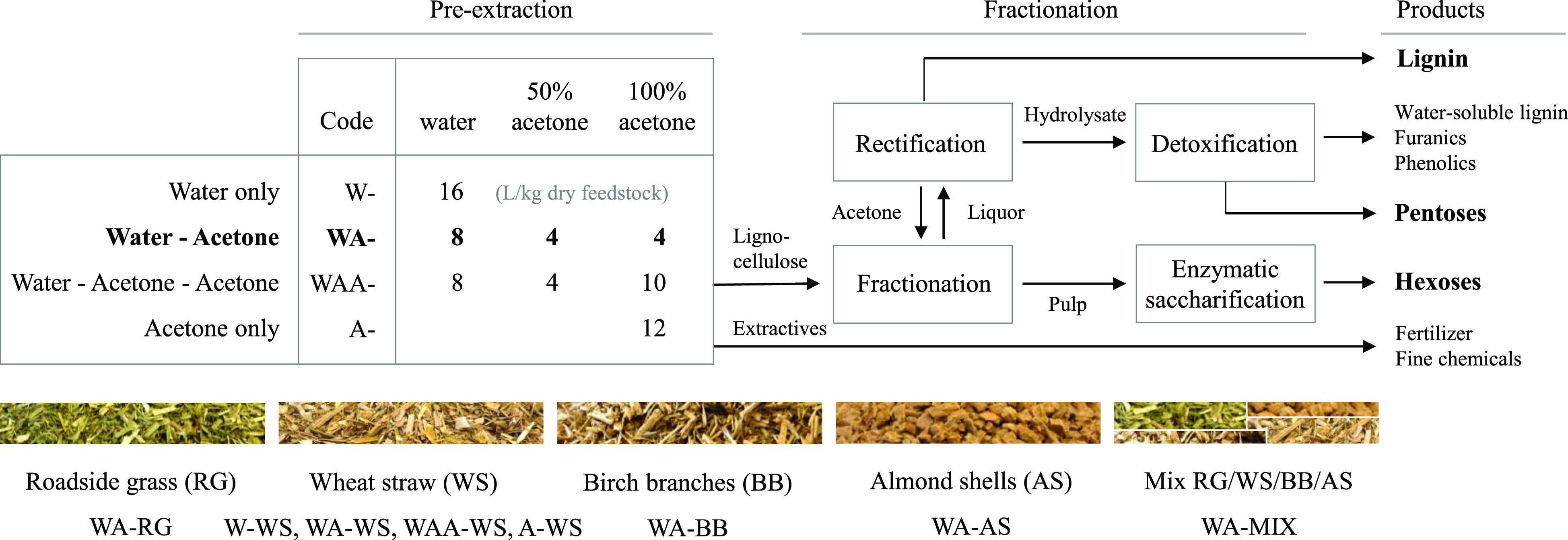
Experimental design.WS was either pre-extracted with water only (W-WS), with 95% acetone only (A-WS), with water followed by 50% acetone followed by 100% acetone (WA–WS), or as WA–WS but with 2.5 times more 100% acetone (WAA–WS). BB, AS, RG, and a mixture of 25% of each feedstock were pre-extracted with the WA method only. The table depicts liquid-to-solid ratios used of the solvents in each of the pre-extraction tests. Untreated and pre-extracted feedstocks were fractionated at 140 °C for 60 min using 50% aqueous acetone and sulfuric acid (liquor pH 1.8).

In this study, we explored this connection using mild acetone-based organosolv fractionation (the so-called Fabiola process)^23^ as an example of a solvent-based biorefinery process. We incorporated a mild acetone–water-based pre-extraction step in the organosolv process (Figure 1) aiming to maximize extractive removal, while preserving the lignocellulose structure for subsequent fractionation.^24^ Here, we comprehensively assess how choices made upstream in terms of the pre-extraction process design affect the efficiency of downstream fractionation, further processing, and purity of the obtained pulp, sugar hydrolysate, and lignin.

## Results and Discussion

### Feedstock Selection and Composition

**Figure 2:**
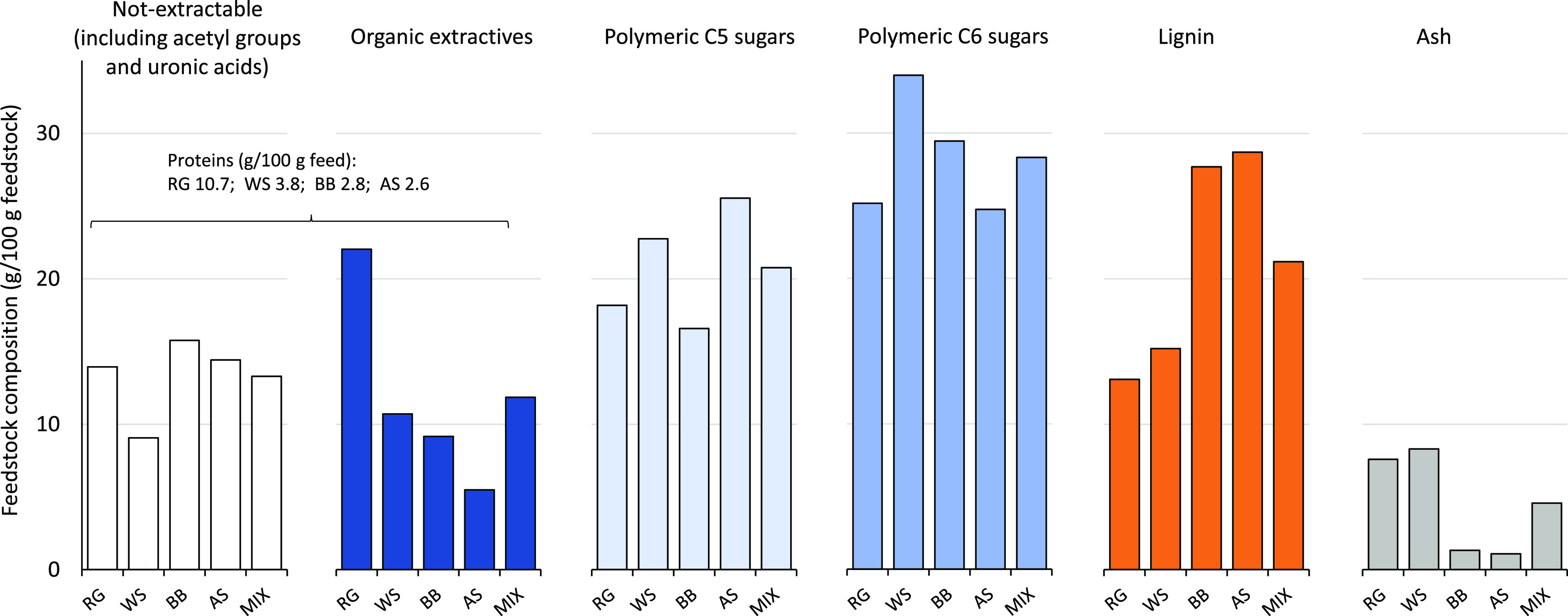
Feedstock composition (dry basis). Not extractable: 100%—feedstock organic extractives, lignocellulose, and ash content. Organic extractives are the sum of water-soluble and solvent-soluble organic extractives. Distribution of extractable and nonextractable proteins was not determined. Polymeric C5 sugars: arabinan and xylan and polymeric C6 sugars: glucan, galactan, mannan, and rhamnan.

Four different feedstocks were selected, considering availability, biomass type (herbaceous and hardwood), origin (agricultural, forestry, and food-processing residues), bulk density, extractives content, and mineral content (Figure 2). This feedstock range allowed us to assess pre-extraction efficiency and fractionation performance and understand the effects of extractives and their removal on product composition, rather than prioritizing on competitiveness and/or intrinsic suitability for biorefining.

Roadside verges have been identified as an underutilized source of biomass.^25,26^ Roadside grass (RG) is characterized by a high organic extractive and mineral content (Figure 2 and Tables S1–S6) and is thus an excellent feed to assess the impact of pre-extraction. RG has a low bulk density (212 kg/m^3^) and a lignocellulose composition representative of herbaceous biomass with a relatively high polymeric sugar (arabinoxylan and glucan) and low lignin content (Figure 2 and Tables S1–S3). RG has a high protein content of 10.7%, consisting of extractable and structurally bound proteins. Hemicellulosic acetyl groups, uronic acid content, and summative composition are available in the Supporting Information (Table S3). The relatively high content of extractive sugars (7.2% of the total feedstock weight) is added to the polymeric C6 and C5 sugars but may consist of monomeric sugars.

Wheat straw (WS) is an interesting feedstock due to its high availability^27^ and extractive composition. The WS epicuticular layer is rich in hydrophobic compounds that serve as protection against dehydration, UV radiation, and parasites. Extractives include free fatty acids such as palmitic acid (C16), pentadecanoic acid (C15), and myristic acid (C14), as well as lipophilic resin acids, sterols, waxes, steryl esters, and mono-, di-, and triglycerides.^28^ In addition, hydrophilic compounds such as phenolic substances, fatty acids, sugar alcohols, glycerides, resin acids, fatty alcohols, triterpenes, and hydrocarbons were identified using acetone as an extraction solvent.^29^ WS bulk density (195 kg/m^3^) and relative lignocellulose composition are relatively similar to RG, but WS has a lower extractive content, which is in line with the literature.^30,31^

Forestry residues are an abundant waste stream worldwide, rich in both lignocellulose and bioactive compounds^32,33^ with birch branches (BB) and bark being underutilized residues. While stem wood extractives contain mainly low molecular weight compounds such as sugars, free fatty acids, and phenolics,^34^ the extractives in birch bark are more diverse and include ether oils, saponins, tannins, hydrocarbons, flavonoids, coumarins, carotenoids, and terpenoids. Major bark components are triterpenoid lupine derivatives such as betulin, which are interesting bioactive compounds for application in drugs, cosmetics, dietary supplements, biocides, and bactericides.^35−37^ The relative abundance of aliphatic suberin can exceed 50% in the extractive-free outer bark of *Betula pendula,* and its derivatives are potential building blocks for polymer synthesis.^38,39^ Typically, BB and especially bark contain more extractives, lignin, ash, and less sugars as compared to birch stem wood.^40^ BB has a higher bulk density (325 kg/m^3^) and a lignocellulose composition similar to hardwood with lower polymeric sugar and higher lignin content.

Finally, almond shells (AS) were selected as the food-processing residue of moderate availability.^41^ The shells have a low organic extractive, protein, and mineral content. AS extractives contain triterpenoids (such as betulinic acid, oleanolic acid, and ursolic acid), lactones, phenolics, and sterols, which are interesting bioactive compounds for dietary and pharmaceutical applications.^42−45^ AS has the highest bulk density (605 kg/m^3^) and is characterized by a relatively high arabinoxylan and low glucan content.

### Pre-Extraction

The pre-extraction process design involves multiple extraction steps, rather than a single extraction process with a water–solvent mixture. Here, aqueous extraction is followed by extraction with 50% w/w acetone and finally 100% acetone (sequence denoted as WA). Extracting first with water has the benefit of separating extractives on polarity. Additionally, it can enable the recycling of nutrients and minerals from agricultural residues back to land to avoid soil depletion. Biomass pre-extraction was conducted in a 14 L custom-built, automated extraction unit that can percolate water and acetone over a preheated biomass bed (Figure S1). WA extractions were conducted on all feedstocks by consecutive down-flow percolation of the preheated solvents. Extraction efficiency was studied initially at various temperatures (50–120 °C) using pressurized solvent extraction, showing improved removal of lipophilic extractives from a selection of feedstocks at the highest temperatures (data not shown). However, to prevent degradation of fine chemicals and bioactive compounds in the extracts, a temperature of 50 °C was selected for extraction [see the Supporting Information for extraction details, Table S7]. Extractions were conducted as single experiments; duplicate WA extractions on RG, WS, and AS, though showed the solid recovery to be reproducible with a relative standard deviation below 0.8%.

**Figure 3:**
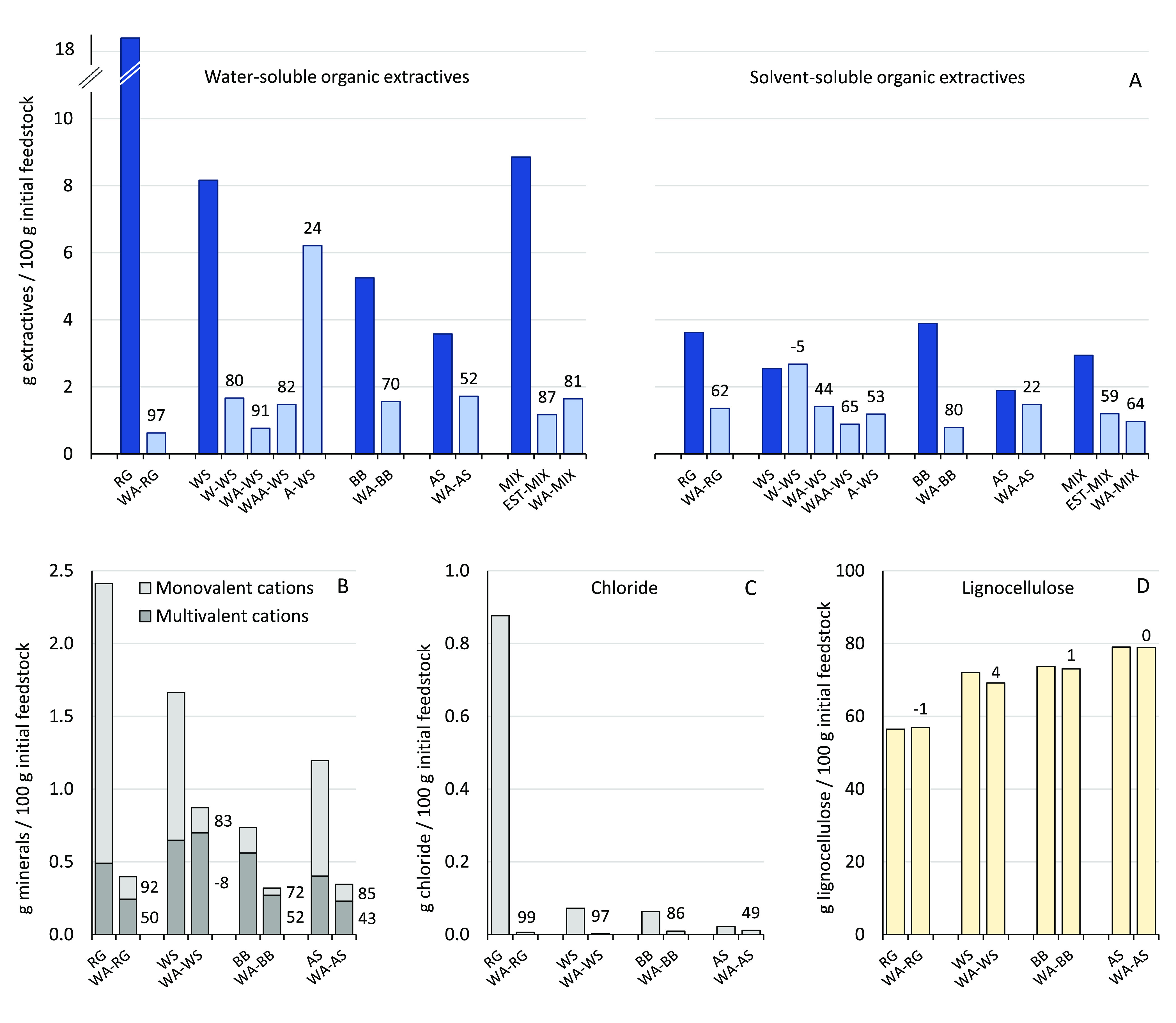
Organic extractives (A), minerals (B), chloride (C), and lignocellulose content (D) of untreated and pre-extracted feedstocks expressed as g/100 g initial untreated feedstock (dry weight basis). Labels represent the percent removal after pre-extraction (negative values may result from experimental error and feedstock composition heterogeneity). EST–MIX shows the values for the feedstock mixture estimated from the single feedstock extraction results. Solvent-soluble organic extractives are the sum of extractives soluble in 50% acetone, 100% acetone, and 100% pentanone. Monovalent cations include K and Na; multivalent cations include, among others, Ca, Mg, Fe, Al, Ni, Cr, Mn, and Zn. Lignocellulose content is the summative value of the contents of hexose/pentose sugars and total lignin in the extracted feedstocks corrected for mass loss after extraction (g/100 g of initial untreated feedstock).

For WS, three additional extraction experiments were conducted. WS aqueous extraction (W–WS) consisted of an extraction with water only to remove water-soluble extractives. WAA–WS included an additional 100% acetone extraction to potentially increase lipophilic extractive removal. Extraction with 95% acetone (A–WS) involved extraction of the straw with 95% acetone as a single solvent without prewetting or aqueous extraction of the straw. The results are summarized in Figure 3 (see also Tables S8 and S9).

The total extractive content of untreated and pre-extracted feedstocks was determined with a modified NREL protocol using sequential sample extraction with water, 50% acetone, 100% acetone, and 100% pentanone at 100 °C. Water-soluble extractives were corrected for the solubilized feedstock ash during extraction. The 50% acetone, 100% acetone, and 100% pentanone extractives were summed as solvent-soluble organic extractives (see Figure S3 for details).

The dark blue bars in Figure 3A denote the total amount of extractives, and the light blue bars represent the remaining extractives after the particular extraction. Notably, WA–RG pre-extraction removed 97% of the water-soluble organic extractives, 99% chloride, and 89% potassium, while preserving the lignocellulose fraction. The design of the pre-extraction unit, the applied feedstock particle size, and the liquid-to-solid ratio are thus sufficient to access all parts of the grass. Extraction efficiency of chloride, one of the most mobile components, ranged from 99 to 49% and decreased as RG ∼ WS > BB ≫ AS, indicating an effect of particle shape, structure, and biomass particle density on extraction efficiency (Figure 3C). A similar trend is observed for water-soluble organic extractives. Despite some variations due to experimental error, the removal of water-soluble organic extractives is more or less similar for the W–WS and aqueous acetone extractions (WA–WS and WAA–WS), indicating a plateau in extraction efficiency at 50 °C (total feedstock extractive content was determined at a temperature of 100 °C). A–WS showed a low extraction efficiency of water-soluble organic compounds. The extraction of a mixed stream of complex biomass (WA–MIX) consisting of equal parts of RG, WS, BB, and AS (MIX) was consistent with the calculated average results of the extraction of the single feedstocks (EST–MIX). Protein was only partially removed from the feedstock with a maximum of 56% for RG (Table S3). Protein extraction largely depends on the feedstock type, the biological function of protein (storage and functional proteins), and its positioning in the biomass ultrastructure. The neutral conditions during pre-extraction are unfavorable for protein solubilization as the pH is close to their isoelectric point. Protein extraction typically improves under alkaline conditions.^46,47^ However, such conditions may result in undesired hemicellulose and lignin extraction and do not match the subsequent acid-catalyzed organosolv fractionation.

Pre-extraction removed most of the monovalent cations such as potassium and sodium, while removal of multivalent cations such as calcium, magnesium, and iron was significantly lower (Figures 3, S14, Tables S3 and S4), in line with the results reported for neutral aqueous extraction of bamboo.^48^

Differences in the solvent-soluble organic extractive content were less pronounced than the water-soluble organic extractive content. Besides feedstock density effects, the extractive composition and solubility in organic solvent play an important role. WS contains lipophilic compounds such as waxes and triglycerides, and for example, BB contain more hydrophilic terpenoids and phenolics.^28,36^

The extraction procedure clearly translated into large differences in WS extractive removal, highlighting the importance of using the adopted gradient of water–acetone mixtures to sequentially solubilize extractives with various polarities. W–WS did not remove any solvent-soluble organic extractives, while the extraction with additional acetone (WAA–WS) removed more extractives than WA–WS. WS extraction with 95% acetone (A–WS) efficiently removed lipophilic extractives (soluble in pure acetone) but removed less extractives soluble in 50% acetone as compared to WA–WS and WAA–WS (Figure S3).

An important prerequisite for the comparison of the untreated and pre-extracted feedstock fractionation is the preservation of lignocellulose during pre-extraction. Therefore, the lignocellulose content (expressed as g/100 g initial feedstock; Figure 3D) of the pre-extracted feedstocks was corrected for mass loss after extraction. Notably, for all experiments, no significant loss of lignocellulose was observed in any of the pretreatments. Loss of hemicellulose acetyl groups is also likely to be minimal under these conditions.^48^ In general, extractive removal results in a higher lignocellulose content in the extracted feedstocks of 79, 77, 79, and 81% (i.e., g/100 g of extracted feedstock) for WA–RG, WA–WS, WA–BB, and WA–AS, respectively.

### Fractionation

(Partial) removal of minerals and (polar and less polar) extractives allowed for a detailed study of the effects of these extractives on fractionation performance and product purity. Mild acetone organosolv treatment of untreated and pre-extracted feedstocks was conducted at a 2 kg scale (liquid-to-solid ratio of 6 L/kg of dry feedstock) using 50% w/w aqueous acetone at a temperature of 140 °C for 60 min.

To ensure fractionation at similar pH, small-scale organosolv screening experiments were conducted for all feedstocks with varying acid concentrations to determine the acid dose required for a liquor pH of 1.8 (Figures S4, S5, and S7). The results translated well to the larger scale fractionation of untreated and pre-extracted feedstocks as all liquors were found to have a pH in the range of 1.8–1.9. Biomass pre-extraction resulted in a significant reduction of acid use for fractionation (and hence less sulfate is present in the pulp and hydrolysate). For RG, the acid dose was reduced from 92 to 42 kg sulfuric acid/tonne (WA-)RG and for WS from 61 to 38 kg per tonne (WA-)WS. After fractionation, the obtained slurry was filtered and the solids were washed with 3 L of 50% aqueous acetone/kg initial dry feedstock and water to remove acetone from the solids. The washed cellulose-rich solid fraction is referred to as the pulp. The liquor and aqueous acetone wash liquor were combined, and lignin was precipitated from this mixture by direct evaporation of acetone. The isolated lignin wash liquid was combined with the lignin-lean aqueous fraction obtained after lignin precipitation. Herein, the hemicellulose-rich liquid fraction recovered from the precipitation process is referred to as the hemicellulose hydrolysate. For further details of the methodology, see Supporting Information.

### Pulp

**Figure 4:**
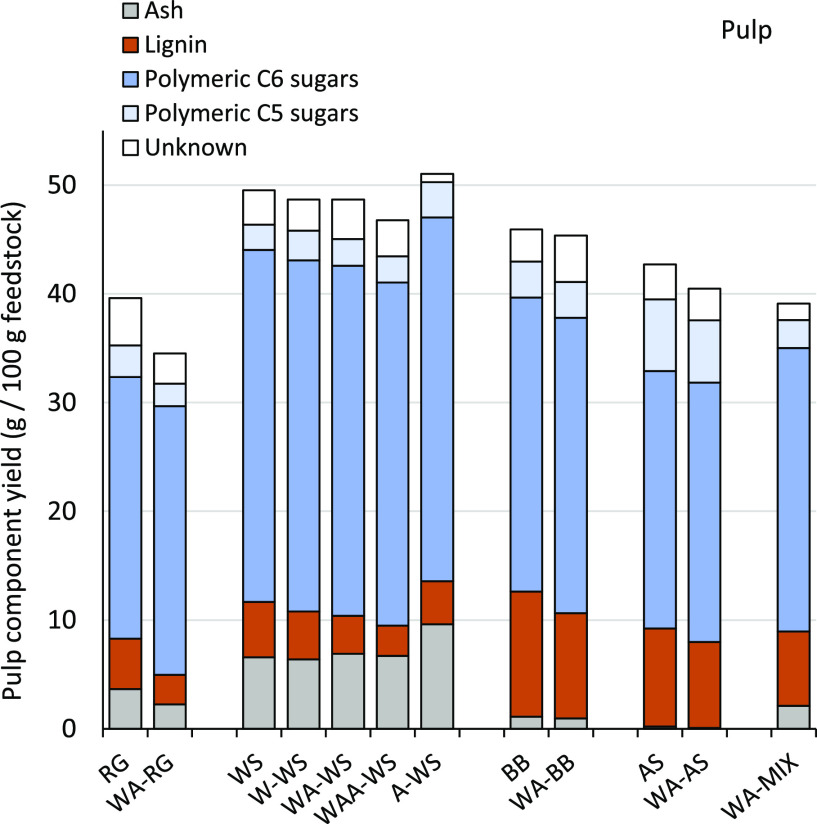
Yield of pulp components expressed as g of component per 100 g of the initial feedstock (dry weight basis).

Pulp yield and composition are shown in Figure 4 (and Table S17), where glucan enrichment is observed for all pulps. RG and WS pulps are characterized by a low lignin and high ash content and BB and AS by a higher lignin and lower ash content, in line with the feedstock characteristics.

The polymeric C6 sugars were mainly recovered in the pulp (Figure S7 and Table S10), with less than 12% being hydrolyzed and lost to the liquor. All pre-extracted feedstocks showed a larger degree of delignification (i.e., lower pulp lignin content) compared to the untreated feedstocks. It is unclear to what extent this can be attributed to improved fractionation or reduced (condensed) extractive precipitation onto the pulp. Unfortunately, the complexity of extractive composition and possible reaction pathways prevent their further characterization and quantification in the products. Additional delignification and ash removal can be achieved by bleaching and alkaline treatment, respectively, but the added processing costs need to be carefully weighed against the gain in the product value.

WA pretreatment slightly improved glucan enrichment due to increased C5 sugar and lignin removal. Difference in the remaining ash content is mainly feedstock-dependent and less affected by pre-extraction or acid dosage. Biomass aqueous pre-extraction has been shown to retain silica and calcium and to only partially remove magnesium and iron, as observed also in this study.^48−50^ Mild acetone organosolv treatment resulted in further solubilization of potassium, sodium, magnesium, and iron, while calcium (such as gypsum), aluminum, and silica accumulated in the pulp ash (Figure S14 and Table S18).

Pulp extractive content was again determined using the water, 50 and 100% acetone, and pentanone extraction. As expected, pulp from the untreated feedstocks (and W–WS) consistently had more extractives as compared to the pulp obtained from the aqueous acetone pre-extracted feedstocks (Figure S15). Precise gravimetric quantification of the pulp extractive content is difficult as the correction for extracted lignin is based on UV spectroscopy only. Analysis of the composition of the WS and WAA–WS pulp extractives by thermal desorption gas chromatography (GC)/mass spectroscopy (MS) analysis (Figure S16) showed a higher fatty acid content in WS than in the WAA–WS pulp. Partly, these fatty acids may be thermal decomposition products of wax esters, and the myristic and palmitic acids containing di- and triglycerides that are abundant in lipophilic extractives of WS.^28,51^ The fatty alcohols hepta- and tetracosanol were not removed by pre-extraction, being less soluble in acetone. Surprisingly, long-chain *n*-alkanes such as pentacosane and triacontane were only found in the pulp from untreated WS. However, the assignments for the *n*-fatty alcohols and *n*-alkanes identified in the (WAA-) WS pulp should be taken with caution, given the low match factors with the NIST library.

Cellulose-enriched pulp can also be valorized by enzymatic saccharification to monomeric sugars for subsequent fermentation to fuels and chemicals.^52−54^ The key to efficient pulp cellulose saccharification is to increase cellulose accessibility for the hydrolytic enzymes.^55^ Organosolv processes were previously shown to produce pulp with high enzymatic digestibility.^56−58^ We assessed the digestibility of pulps obtained from untreated and pre-extracted feedstocks using an enzyme solution MetZyme SUNO 036 from MetGen (see Figure S17 for yields).

For the RG and WS pulps, near-complete saccharification was reached using an enzyme dose of 0.15 g MetZyme SUNO 036 enzyme solution/g pulp glucan, while glucose yields for the BB and AS pulps are low at this loading. The higher pulp lignin content of BB and AS likely negatively affects saccharification, with lignin both reducing cellulose accessibility and causing enzyme deactivation, for example, by adsorption or inhibition with lignin-derived solubles.^59−61^ However, the effect of soluble lignin-derived compounds is believed to be low, as BB pulp post-processing at 140 °C for 60 min and hot washing of the pulp to remove such compounds did not improve saccharification (Figure S18). The presence of lignin-condensed phenolic moieties, such as those found in bark, may also play a role in nonproductive binding of enzymes to lignin.^59,62−64^ Increasing the enzyme dose to 0.5 g of enzyme solution/g pulp glucan increased the glucose yield, demonstrating that the glucan in the BB and AS pulp is in principle susceptible to enzymatic saccharification. However, such a high enzyme dose comes with a cost that will negatively impact process viability.^65,66^ Bleaching of the WA–BB pulp removed residual lignin and significantly improved glucose yield, demonstrating that residual pulp lignin indeed plays a major role in the saccharification of organosolv pulps (data not shown). Post-treatment of the BB pulp using higher temperatures can remove part of the pulp lignin, but such increased delignification (from 58 to 76% after 60 min post-treatment at 180 °C and 5 mM sulfuric acid) only resulted in minor improvement of the glucose yield (Figure S18).

The pulps obtained from the untreated or pre-extracted (WA) feedstocks showed similar saccharification rates. Apparently, the reduced lignin, extractives, and fatty acid content in pulps from pre-extracted feedstocks do not significantly enhance enzymatic cellulose hydrolysis.

### Hemicellulose Hydrolysate

**Figure 5:**
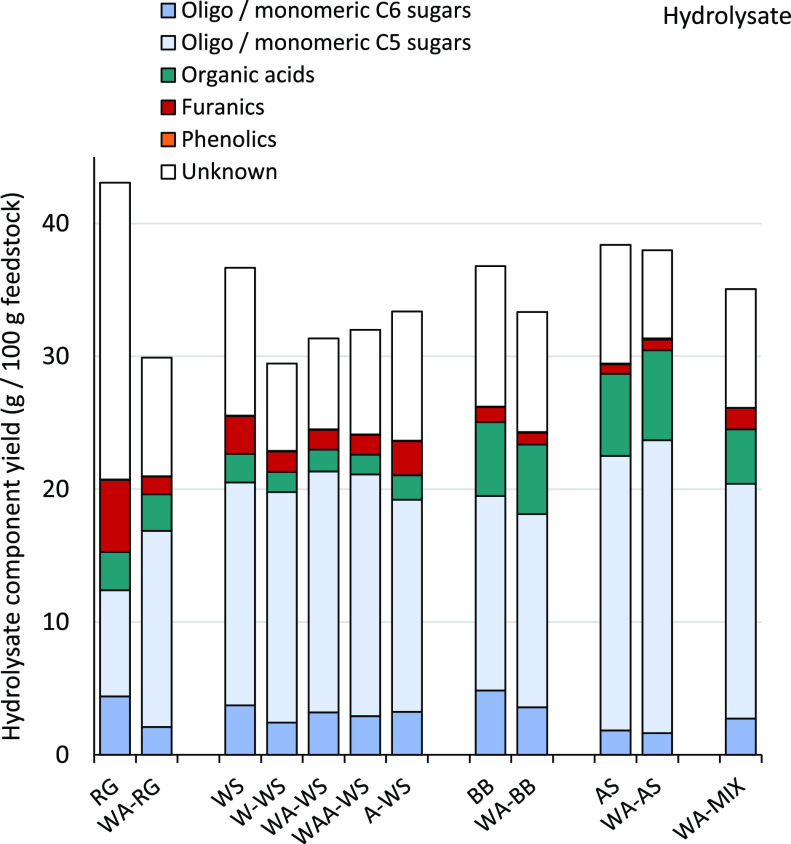
Yield of hydrolysate components expressed as g of component per 100 g of the initial feedstock (dry weight basis).

Various chemocatalytic and biotechnological routes have been reported for the valorization of the monomeric sugars that mainly make up the hemicellulose hydrolysate.^67−69^ The mass balance in Figure 5 represents the summative values of water-soluble fractionation products from the combined streams (liquor and pulp/lignin washings; see Figure S6).

Biomass pre-extraction positively influenced the fractionation product distribution. A considerable amount of the feedstock C5 sugars was solubilized as monomers (see also Figure S7 and Table S10). For RG, pre-extraction improved monomeric C5 sugar yield from 35% (RG) to 66% (WA–RG) of the initial feedstock C5 sugars. Sugar degradation to furfural was significantly reduced from 26% (RG) to 9% (WA–RG) of the initial feedstock C5 sugars.

For WS, solubilized C5 monomer yields improved from 60% (WS) to 69%, 72, and 74% for W–WS, WA–WS, and WAA–WS, respectively, with concomitant reduction in sugar degradation to furfural [from 15 to 9% for (WA-) WS]. The effect of inefficient water-soluble extractive and mineral removal with A–WS is directly reflected in a higher furfural yield of 14%. BB and AS showed comparable results for untreated and pre-extracted feedstocks, showing a high yield of monomeric sugars, limited furfural formation, and a larger retention of polymeric C5 sugars in the pulp than RG and WS.

Degradation of C6 sugars to HMF is very minor, except for RG where its high extractive sugar content (Figure S7 and Table S6) led to immediate sugar release into the liquor during fractionation, resulting in increased degradation. Acetic acid is the major component of the solubilized organic acids and is produced mostly from deacetylation of hemicellulose. Very low concentrations of lignin-derived phenolics (mainly vanillin and syringaldehyde, Table S14) were found in the hydrolysates.

A significant part of the hydrolysate composition consists of various unidentified compounds (Figure 5, white bars), which were quantified indirectly from the feedstock and product mass balance excluding possible gas formation (see the Supporting Information for a detailed discussion).

The unidentified water-soluble organics may include proteins, uronic acids, and organic extractives, as well as water-soluble lignin (WSL) and the sugar derivatives missing in the RG and WS mass balances. While it is clear that pre-extraction reduced the amount of unidentified compounds in the hydrolysate, that is, by 60, 38, 14, and 26% for WA–RG, WA–WS, WA–BB, and WA–AS, respectively, a significant amount is still present in the hydrolysates of pre-extracted biomass. For example, the RG protein content is only reduced from 10.7 to 6.5% w/w after pre-extraction as part of the protein fraction was incorporated in the macrostructure of the grass.

Inorganic salts in the hydrolysate are included in the “unknown” category in Figure 5. Cations (mostly monovalent) represent only a minor fraction of the total hydrolysate composition (<5%).

However, especially RG and WS hydrolysates have relatively high concentrations of potassium and chloride. Pre-extraction removed most of these elements, greatly reducing their concentration in hydrolysates. Pre-extraction also significantly reduced hydrolysate sulfate concentrations as less sulfuric acid was needed for fractionation. Valorization of the hemicellulose-derived sugars in the hydrolysate can, for example, be pursued by fermentation. A reduced salt content in the hydrolysates is then beneficial, as this can limit the osmotic stress and ion toxicity experienced by fermentation microorganisms.^70^ However, sugar fermentation would require hydrolysate detoxification by removal of other fermentation inhibitors such as organic acids, furanics, and phenolics. The required extent of removal largely depends on the tolerance of the fermentation strain to certain inhibitor types.^71−73^ Notably, hydrolysate detoxification with activated charcoal was more efficient for the hydrolysates of pre-extracted feedstock, as indicated by the extent of discoloration (Figure S19), showing the impact of pretreatment on downstream processing and reducing the costs involved. The adsorption capacities of the charcoal for organic acids, furanics, and phenolics were found to be comparable for the detoxification of hydrolysates from untreated and pre-extracted feedstocks (Figure S20). This indicates that there were no specific effects from the extractive content in the various hydrolysates. However, lower furfural formation during fractionation of pre-extracted feedstocks does lower the amount of sorbent needed for detoxification.

### Lignin

**Figure 6:**
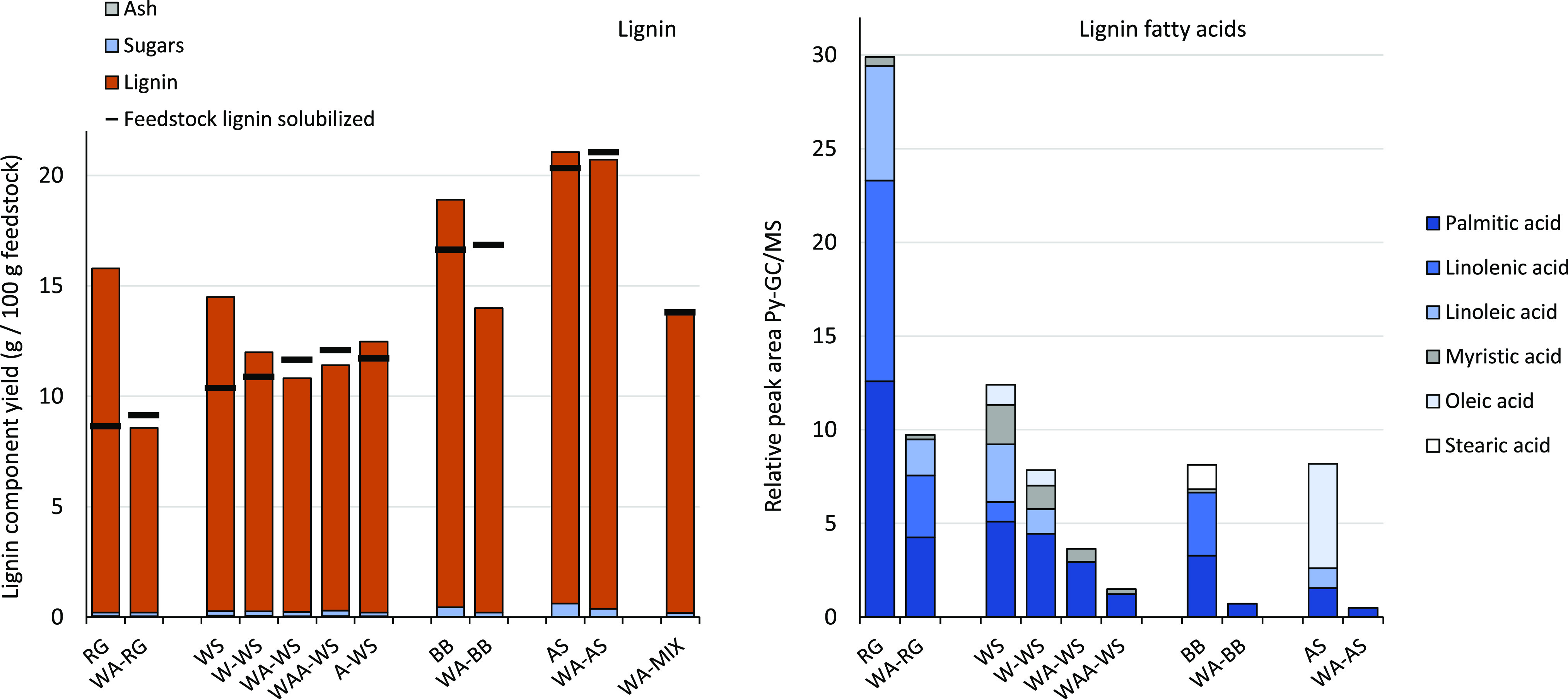
Left: yield of lignin components expressed as g of component per 100 g of the initial feedstock (dry weight basis). The black bars represent how much of the feedstock lignin was solubilized in the liquor. Right: relative peak area of fatty acids detected by Py-GC/MS of isolated lignin from untreated and pre-extracted feedstocks.

The effect of pretreatment on lignin purity is shown in Figure 6. The black markers represent how much feedstock lignin was solubilized, and the orange bars represent how much was precipitated in total. Pseudolignin formation caused the total solubilized “lignin” recovery to exceed 100% as seen for RG, WS, BB, and AS (orange bar is higher than the black marker, as also noted in Figure S7). The same was observed for both W–WS and A–WS, indicating that water-soluble as well as solvent-soluble extractives may affect lignin purity. Notably, the untreated feedstock extractive content was more than enough to account for the excess “lignin” in the mass balance (Figure S8). For all WA experiments and the WAA–WS experiment, lignin recovery was less than the amount available in the liquor. The missing mass consists likely of WSL oligomers rather than monomers, as the phenolic content of the liquor is low (Table S14). Note that the amount of WSL is underestimated, as all precipitated lignins can contain impurities. For example, ∼10% of the furfural formed likely coprecipitated with the lignin, adding from 0.2 (AS) to 3.1 (RG) % w/w to the precipitated lignins (Figure S21). The sugar (mono/oligomeric and lignin–carbohydrate complexes) and ash content are low for all isolated lignins (Figure 6, Tables S21 and S22).

Pyrolysis-GC (Py-GC)–MS analysis can be used to estimate, among others, lignin fatty acid content and provide insights into protein impurities and solvent condensation products in the lignin. Pre-extraction significantly reduced the fatty acid abundance in the isolated lignins (Figure 6). RG lignin shows the highest abundance of fatty acids, in accordance with the high solvent extractive content and typical compositions (palmitic-, linolenic-, and linoleic acids) reported for grasses.^74^ del Río et al. reported a 2% lipophilic (acetone) extractive content in WS, mainly composed of fatty acids.^75^ The fractionation of WS produced 10 g liquor-solubilized lignin/100 g feedstock, and thus, partial coprecipitation of the 2.6 g solvent extractives/100 g WS can significantly contribute to lignin impurity. Indeed, as expected, W–WS showed a higher (and WAA–WS lower) fatty acid abundance in the lignin fraction as compared to WA–WS. Suberin (derivatives) are not thought to contribute significantly to the impurities in BB lignin, given the mild acid conditions applied for fractionation and lack of α,ω-diacids, ω-hydroxyacids Py-GC/MS markers typical for suberin.^76^

On average, WA-pre-extraction decreased fatty acid abundance in lignins by 75% (Figure S22 and Table S23), considerably improving the purity of the lignins. *N*-Heterocyclic compounds are typical pyrolysis products for proteins.^77^ Py-GC/MS also provides markers for protein impurities in the lignin. For RG and AS, the summative relative peak area of nitrogen containing pyrolysis products such as pyridines and pyrroles amounted to 2.2 and 0.5%, respectively, indeed indicating the presence of protein (derivatives) in the isolated lignins. Traces of the acetone self-condensation products diacetone alcohol (DAA) and mesityloxide (MO) could also be identified in the Py-GC/MS chromatograms of the isolated lignins from either the untreated or pre-extracted feedstocks (Table S23). Total acetone loss through DAA/MO formation was generally the same, ranging from 0.4 to 0.7% w/w (excluding outlier RG, Table S26).

Size exclusion chromatography analysis shows that, regardless of the observed differences in lignin yield and purity, lignin molecular weight and polydispersity did not differ significantly for the untreated and pre-extracted samples (Table S24). ^31^P NMR hydroxyl content analysis of the lignin samples from the pre-extracted feedstocks showed the aliphatic-, phenolic-, and total OH content to be slightly higher and the COOH content lower than the untreated ones, with AS as the exception. The higher COOH content for RG and WS lignin may originate partly from the fatty acids present in the lignin. 2D HSQC–NMR analysis of a selection of the isolated lignins provided a detailed insight into lignin aromatic unit composition and interunit linkages (Table S24). For comparison, a WS cellulolytic enzymatic lignin (CEL) was also prepared. The monolignol ratio was generally in accordance with the literature.^78−82^ Pre-extraction did not cause any significant changes in lignin aromatic unit composition, except for lower *p*-hydroxyphenyl (H) content in the pre-extracted lignin samples. This is likely caused by cross-peak overlap with protein-derived phenylalanine, indicating the presence of proteins in the lignin.^83^

The primary mechanism for lignin depolymerization and subsequent solubilization during mild acetone organosolv is acidolysis of the β–O–4 bonds. Isolated lignins showed only minor variations in linkage abundance. While β–O–4 and β–β contents in WS lignin were significantly lower than that in CEL lignin, abundance was still relatively high for an organosolv lignin because of applied mild fractionation conditions. 2D HSQC–NMR signal intensities from fatty acids are in line with the Py-GC/MS results, showing a significant decrease for WA-lignins. Furthermore, quantification of the whole alkyl region shows a significant decrease in intensity with the largest difference seen for (WA-) RG and smallest for (WA-) AS.

A significant part of the signal intensity of the alkyl region typically originates from nonlignin compounds and a reduction therein again points to increased lignin purity. 2D HSCQ–NMR spectra, the quantification of lignin structures, and methodology details can be found in the Supporting Information (Figure S26 and Tables S24–S26). Importantly, taken together, the lignin analysis data clearly show that combining pre-extraction and mild acetone organosolv fractionation provides access to high quality lignins from a wide variety of lignocellulosic feedstocks.

### Process Considerations

The results mentioned above show pre-extraction can to a great extent mitigate the challenges associated with fractionation of various feedstocks of heterogeneous composition. Thus, economic and sustainability advances include the extended feedstock pool to include cheaper biomass sources, improved product purity, increased biorefinery output (via valorization of extractives), and reduced use of chemicals (acid and lime). However, an additional process unit (and associated unit operations) implies increased capital and operational costs. A process model based on higher TRL biorefining data can provide insights into the effect of the additional process step on potential solvent loss and energy use for acetone recycling.

Promisingly, first trials with consecutive pilot-scale pre-extraction and fractionation were successfully conducted in a 460 L percolating reactor (Fraunhofer Center for Chemical-Biotechnological Processes CBP, Leuna, Germany) using 24–50 kg WS, BB, and a mixture thereof. In these trials, pre-extraction was followed directly by fractionation of the wet-extracted biomass in the percolating reactor. Based on these scale-up results, a first conceptual process design for the pre-extraction process was constructed in Aspen Plus (AspenTech, 2021), including column modeling using the non-random two-liquid model. This was done for BB using an aqueous extraction followed by an extraction with 75% aqueous acetone using 4 kg of water and 8 kg of aqueous acetone per kg dry BB, respectively. Acetone is recovered from the extracts in a distillation column. The pre-extraction and organosolv process model included transfer of adsorbed acetone in the extracted biomass to the fractionation process and a recycling of part of the acetone (recovered from the fractionation liquor) to the pre-extraction unit rather than to the fractionation reactor.

Process modeling indicated that it is feasible to recover 99.999% of the aqueous acetone used for pre-extraction using a 25-stage column with a reflux ratio of 0.335. Heat integration was done for the pre-extraction and organosolv fractionation sections separately. The heat demand for pre-extraction (mainly for the reboiler of the acetone recovery column) amounts to 3.8 MJ/kg of dry weight biomass feed, which is in the same range as the heat requirement for the organosolv fractionation process. Assuming a price of 13 EUR/GJ for heat from a biomass boiler, the estimated energy cost of pre-extraction is 50 EUR/tonne dry weight biomass. Other operational and capital costs are not included as these largely depend on the specific process design and optimization. One such optimization step is to minimize water usage by integrating the pulp water wash and aqueous waste streams from sugar fermentation with the aqueous pre-extraction. Detailed techno-economic and life cycle studies, focusing on feedstock selection, supply logistics, and outlets for each generated product to complete the value chain, will provide further insights into the economic impact and sustainability credentials of this integrated biorefinery process.

## Conclusions

Including a biomass pre-extraction step prior to fractionation proved to be a highly versatile strategy for enhanced mild acetone organosolv biorefining of a diverse selection of heterogeneous and mixed lignocellulosic biomass feeds. Benefits include a reduced lignin content of the pulps, an increase in hemicellulose sugar yield, and a higher lignin purity. Lignins from mild acetone organosolv fractionation can potentially be used in high value-added material applications such as coatings, foams, and resins, provided that a high purity lignin stream can be generated with minimal quality variation. The results show that pre-extraction can contribute to this aim.

Downstream advantages of pre-extraction included less sugar degradation products (i.e., furfural), which is beneficial for further valorization of hemicellulose sugars via, for example, fermentation. Notably, enzymatic pulp saccharification rates were not affected by inclusion of the pre-extraction process step. Pulps from the herbaceous feedstocks RG and WS proved much more amenable to saccharification than the BB and AS pulps, as attributed to the differences in delignification extent.

From a process point of view, pre-extraction significantly reduced acid dose requirements for fractionation, especially for the herbaceous feedstocks. The combination of lower sulfate concentrations and the removal of chlorides is anticipated to decrease corrosion rates of the process equipment. The use of a single aqueous solvent for pre-extraction and fractionation greatly simplifies process integration and solvent recovery at a larger scale. A first conceptual design, based on scale-up trials, provided insights into the effect of this additional process step on solvent and energy use. Follow-up modeling studies will assess how any increase in water use and energy demand is offset by improved feedstock utilization, cost savings (e.g., cheaper feedstocks and reduced transport costs), and added revenues (e.g., improved product quality and possibly extract valorization). Overall, pre-extraction process design, flexibility, and optimization depend on various aspects ranging from feedstock characteristics to final product applications that should be considered for optimal integration of the process in biorefinery value chains.

## Supporting Information Available

Additional experimental details, materials, and methods, including photographs of samples and experimental setup (PDF)
