# Supplementary material for: Biomass Pre-Extraction as a Versatile Strategy to Improve Biorefinery Feedstock Flexibility, Sugar Yields, and Lignin Purity
Source: ACS Sustain Chem Eng. 2022 Apr 27;10(18):6012–22. doi: 10.1021/acssuschemeng.2c00838 (PMC9092456; doi:10.1021/acssuschemeng.2c00838)
Supplement: Supplementary file 1 — sc2c00838_si_001.pdf [file sc2c00838_si_001.pdf]

Supporting Information

Biomass pre-extraction as versatile strategy to improve biorefinery feedstock flexibility, sugar yields and lignin purity.

Arjan T. Smit,[a,b] André van Zomeren,[a] Karla Dussan,[a] Luke A. Riddell,[b] Wouter J. J. Huijgen,[a,†] J. W. Dijkstra,[a] and Pieter C. A. Bruijnincx[b]*

a. The Netherlands Organisation for Applied Scientific Research (TNO), unit Energy Transition, Biobased & Circular Technologies group. P.O. Box 1, 1755 ZG Petten, The Netherlands.

* Email: arjan.smit@tno.nl

b. Organic Chemistry and Catalysis, Debye Institute for Nanomaterials Science, Utrecht University, Universiteitsweg 99, 3584 CG Utrecht, The Netherlands.

[†] Current affiliation: Cosun R&D, Kreekweg 1, 4670 VA, Dinteloord, The Netherlands.

Number of pages: 51

Number of figures: 26

Number of tables: 27

Number of schemes: 0

Contents

Feedstock composition. 6

Pre-extraction 9

Acid dose screening for fractionation 13

Fractionation 15

Pulp 27

Hemicellulose hydrolysate 33

Lignin 36

List of Tables

Table S1 Extractives content of (pre-extracted) feedstocks. 7
Table S2 Composition of (pre-extracted) feedstocks. 7
Table S3 Protein, uronic acid, acetyl and mineral content of (pre-extracted) feedstocks. 8
Table S4 Inorganic elemental composition of (extracted) feedstocks. 8
Table S5 Elemental composition of (pre-extracted) feedstocks. 9
Table S6 Extractives sugar content. 9
Table S7 Experimental conditions for biomass pre-extraction. 10
Table S8 Pre-extraction results. 11
Table S9 Mass balance biomass pre-extraction. 12
Table S10 General fractionation performance. 18
Table S11 Product distribution of hexoses and pentoses. 19
Table S12 Mass of solids and liquids. 21
Table S13 Liquor and hydrolysate sugar composition. 22
Table S14 Liquor and hydrolysate oxygenate composition. 23
Table S15 Composition of (post-treated) birch branches pulp. 27
Table S16 Results of birch branches pulp post-treatment. 27
Table S17 Biochemical composition of pulps. 28
Table S18 Inorganic elemental composition of pulps. 29
Table S19 Indirect quantification of hemicellulose unidentified constituents. 33
Table S20 Assignments and correction factors for integral regions of HSQC-NMR spectra 37
Table S21 Biochemical composition of precipitated lignin. 39
Table S22 Biochemical composition of pulp waterwash lignin. 40
Table S23 Lignin Py-GC/MS response for a selection of components. 42
Table S24 Isolated lignin characteristics 45
Table S25 2D HSQC-NMR semi-quantitative analysis of lignin minor structures. 45
Table S26 Lignin G:S:H ratio as determined by Py-GC/MS and HSQC-NMR. 49
Table S27 Diacetone alcohol and mesityloxide concentrations in fractionation liquors 50

List of Figures

Figure S1 Biomass pre-extraction unit and pre-extraction of roadside grass 10
Figure S2 Biomass extracts 11
Figure S3 Extractives content of untreated and pre-extracted feedstocks 12
Figure S4 Estimated required acid dose for organosolv fractionation. 13
Figure S5 Small scale fractionation screening experiments using a series of acid doses. 14
Figure S6 Mild acetone organosolv fractionation and processing of fractions. 15
Figure S7 Fractionation product distribution 20
Figure S8 Correlation of excess lignin found per fractionation experiment 21
Figure S9 Pictures of feedstock, pulp and precipitated lignin. 24
Figure S10 Birch wood chips, pre-extracted birch branches and pre-extracted hardwood bark composition 25
Figure S11 Birch, birch branches and hardwood bark fractionation results 25
Figure S12 Product distribution for 60 and 120 min fractionation of pre-extracted birch branches and almond shells 26
Figure S13 Combined results of fractionation of pre-extracted birch branches and pulp post-treatment 27
Figure S14 Mineral content of (pre-extracted) feedstock and pulp per fractionation of 1 kg feedstock 29
Figure S15 Pulp extractives content. 30
Figure S16 TD-GC/MS results of wheat straw (WS) and pre-extracted wheat straw (WAA-WS) pulps. 31
Figure S17 Glucose yield from the saccharification of pulps produced from untreated and pre-extracted feedstocks 32
Figure S18 Glucose yields obtained from enzymatic saccharification of post-treated BB pulp 32
Figure S19 Detoxified hydrolysates 34
Figure S20 Detoxification of hydrolysates. 35
Figure S21 Lignin precipitation mass balance closure. 39
Figure S22 Lignin Py-GC/MS response per component group. 41
Figure S23 Correlation lignin volatile fatty acid signal and lignin precipitation yield. 41

Figure S24 Fractionation results of WS and WAA-WS lignin....43
Figure S25 Py-GC/MS analysis results of WS and WAA-WS lignin fractions....44
Figure S26 Aromatic/unsaturated region, oxygenated aliphatic side chain region and alkyl region of the 2D HSQC NMR spectra of lignin obtained from fractionation of the untreated and pre-extracted (WA) feedstocks....49

Feedstock composition

Experimental

Technical acetone was obtained from VWR Chemicals and sulfuric acid (98%) and 3-pentanone from Sigma-Aldrich. Roadside grass (RG) was cut in June 2019 (the Netherlands) and air dried for two months. Ambient-dry wheat straw (WS) was harvested in August 2018 and kindly provided by Itabia (Italy). Birch branches (BB) were harvested in March 2019 and kindly provided by the Dutch Forest management agency (Staatsbosbeheer, the Netherlands). The branches were cut to chips using a wood chipper and air dried. Crushed almond shells (AS, 1-5 mm) were purchased from Biopowder (Schilling LTD, Malta). Roadside grass, wheat straw and chipped birch branches were further cut to a smaller particle size using a Retsch SM300 cutter mill equipped with a 4 mm sieve. A mixture was made containing 25% (on dry weight basis) of milled roadside grass, wheat straw, birch branches and almond shells. The moisture content was determined using an oven at 105 °C.
Feedstock bulk density was determined by the weight and volume of milled ambient-dry feedstocks. The measurements were conducted in quadruplicate by tapping the sample 6 times in a 2 L container (height/width ratio: 1.5).

Feedstock extractives content was determined using water and ethanol according to NREL/TP-510-42619 and a Dionex ASE350 Accelerated Solvent Extractor.[1] A second (modified) protocol was used for determining extractives content and its removal from (pre-extracted) feedstocks prior to biochemical composition analysis. Samples were milled using a Fritz pulverisette equipped with an 0.2 mm sieve except for RG which was milled over an 0.5 mm sieve to prevent clogging of the ASE. A 34 mL extraction cell was fitted with two Whatman GF/D glass fibre filters and 6 gram of sample was weighed into the cell. Samples were extracted at 100 °C with water (3 cycles), 50% v/v acetone (2 cycles), 100% acetone (2 cycles) and 3-pentanone (1 cycle) successively according to NREL/TP-510-42619 extraction conditions. No duplicate extractions were conducted. Extracts were analysed for sugar content after post hydrolysis in 1M sulfuric acid at 100 °C for 2 h. The remaining extract was dried and weighed. Extracted solids were dried, weighed and milled using an IKA laboratory mill for subsequent biochemical composition analysis. The summative composition of solids was determined using procedures described in earlier work.[2] These procedures are modified versions of the NREL standard biomass analytical procedures (NREL/TP-510-42618).[1] In short, the content of lignin and carbohydrates was determined in duplicate as follows: the sample was hydrolysed in two steps: (1) 12 M (72% w/w) H_2SO_4 (30 °C, 1 h) and (2) 1.2 M H_2SO_4 (100 °C, 3 h). The solid residue was determined gravimetrically and its ash content was measured. The acid-insoluble lignin (AIL) content was based on the amount of ash-free residue, and acid-soluble lignin (ASL) was determined using UV–VIS absorption. Finally, the hydrolysate was analysed for monomeric sugars and corrected for sugar degradation. Biomass ash content was determined according to NREL/TP-510-42622.[3] The average relative standard deviation of the biochemical composition analysis was 2.6%. Quadruplicate analyses were conducted in case relevant sugars, lignin or ash showed elevated deviations.

Analysis of monomeric sugars was performed in duplicate by HPAEC-PAD (ICS3000, Dionex) equipped with a CarboPac PA1 column and a post column addition of 0.2 mL/min 0.25 M NaOH. A gradient of NaOH was used as eluent (0.25 mL/min): 15 mM (0–1 min), 0 mM (1–21 min), increasing from 0 to 187.5 mM (21–37 min), 250 mM (37–42 min), decreasing from 250 to 15 mM (42.0-42.1 min) and 15 mM (42.1-50 min). Lactose was used as an internal standard. Samples containing 1.2 M sulfuric acid from biochemical composition analysis were neutralised with barium carbonate and centrifuged before analysis. The average relative standard deviation of the sugar analysis in fractionation liquors and hydrolysates was 2.0%

The (extracted) feedstock elemental composition was measured in duplicate with an elemental analyser (ISO 16948)) and ion chromatography after bomb combustion (Cl) using ISO 16994. Inductively coupled plasma atomic emission spectroscopy (ICP-AES) was used for the other elements after microwave digestion with HCl, HNO_3, H_2O_2 and (in a few samples) HF according to ISO 16967 (major elements) and ISO 16968 (minor elements). The oxygen content was determined by the difference required to make a mass closure of 100% when considering the sum of the C, H, N, S and ash contents. All analyses were performed in duplicate.The (extracted) feedstock protein content was estimated by multiplying the nitrogen content (%) by a factor of 6.25.[4]

Results

Table S1 shows the feedstock extractives content using three extraction cycles with water followed by three extraction cycles with ethanol as described in NREL procedure TP-510-42619. The modified procedure also applied three extraction cycles with water but was followed by two extraction cycles with 50 % v/v aqueous acetone, two cycles with pure acetone and one cycle with pure pentanone. Extraction with water was identical for both procedures and the measured values for organic water-soluble extractives (calculated as the amount of water-soluble extractives minus the soluble ash) are comparable

except for BB. The modified (ketone) procedure shows a higher summative solvent extractives content for all feedstocks. Likely, this is a combined effect of an increased number of extraction cycles as well as the increased polarity range of the applied solvents. Overall, the observed differences are relatively small. Sun et al. showed how the choice of solvent affects the extraction of wheat straw lipophilic components and highest yields of sterols, waxes and triglycerides were obtained using methyl tert-butyl ether (MTBE).[5] Here, pentanone was selected as solvent for the final extraction cycle to check whether any lipophilic extractives remained in the feedstock after extraction with acetone, but only minor amounts were found in WS.

The summative content of identified components (sugars, lignin, ash, protein, acetyl groups and uronic acids) is 85%, 89% and 88% for RG, WS and BB, respectively. The remaining 11-15% consists of both extractable and non-extractable compounds such as chlorophyll, metabolites, fatty acids etc, which were not further quantified. Dewhurst et al. found total fatty acid contents in the range of 1-3% for grass dry matter with significant seasonal and grass species variation.[6] Acetyl groups and uronic acids were not quantified for AS but composition analysis reported by Queiros et al. showed a 4.5% acetyl group and 1% uronic acid content in almond shells.[7] The total mass balance of identified components is generally in line with reported compositional values for almond shells.[7-8]

Table S1 Extractives content of (pre-extracted) feedstocks.

% w/w (dry weight basis)		Extractives (NREL)		Extractives (ketones)			
		Water (not ash)	Ethanol	Water (not ash)	50% acetone	100% acetone	100% 3-pentanone
Roadside grass	RG	19.2	3.4	18.4	3.2	0.4	[a]
	WA-RG	ND	ND	0.9	1.3	0.5	
Wheat straw	WS	8.3	2.2	8.2	2.0	0.4	0.2
	W-WS	ND	ND	1.9	2.4	0.5	0.2
	WA-WS	ND	ND	0.9	1.1	0.3	0.2
	WAA-WS	ND	ND	1.7	0.7	0.2	0.1
	A-WS	ND	ND	6.4	1.0	0.2	
Birch branches	BB	3.8	3.5	5.2	3.5	0.4	
	WA-BB	ND	ND	1.7	0.8	0.1	
Almond shells	AS	3.4	1.5	3.6	1.8	0.1	
	WA-AS	ND	ND	1.8	1.4	0.1	
Mixed stream	WA-MIX	ND	ND	1.9	1.0	0.2	0.1

[a] Empty cell: below detection limit set at 0.1 % w/w. ND Not determined.

Table S2 Composition of (pre-extracted) feedstocks.

% w/w (dry weight basis)		Organic extractives[a]	Polymeric C5 sugars[b]		Polymeric C6 sugars[b]					
		Total	Xylan	Arabinan	Glucan	Galactan	Mannan	Rhamnan	Lignin	Ash
Roadside grass	RG	22.0	15.8	2.4	24.4	0.7	[c]		13.1	7.6
	WA-RG	2.8	23.1	3.4	35.7	0.9			16.2	3.7
Wheat straw	WS	10.7	20.5	2.3	33.4	0.6			15.2	8.3
	W-WS	4.9	21.0	2.4	36.6	0.7	0.5		16.9	7.3
	WA-WS	2.4	21.6	2.4	36.2	0.6			16.7	7.9
	WAA-WS	2.7	20.7	2.6	36.4	0.8	0.5		16.5	8.9
	A-WS	7.7	21.3	2.5	36.3	0.8	0.3		16.1	9.5
Birch branches	BB	9.1	15.6	1.0	26.9	1.0	1.2	0.4	27.7	1.3
	WA-BB	2.5	16.8	1.0	29.6	1.2	1.2	0.5	29.2	1.2
Almond shells	AS	5.5	24.8	0.8	23.5	0.9		0.4	28.7	1.1
	WA-AS	3.3	25.5	0.8	24.4	0.9		0.4	29.5	0.6
Mixed stream	WA-MIX	3.1	21.0	2.1	32.7	1.0			23.6	3.2

[a] Including sugars in extractives (Table S6). [b] Excluding sugars in extractives. [c] Empty cell: below detection limit.

Table S3 Protein, uronic acid, acetyl content and mineral composition of (pre-extracted) feedstocks.

% w/w (dry weight basis)					Ash				
		Protein	Uronic acids	Acetyl	Total	Monovalent cations	Multivalent cations	Phospho-rous	Si [b]
Roadside grass	RG	10.7	2.8	1.1	7.59	1.92	0.49	0.18	0.10
	WA-RG	6.5			3.72	0.22	0.34	0.02	0.47
Wheat straw	WS	3.8	1.8	1.0	8.31	1.02	0.65	0.04	0.13
	W-WS	[a]			7.33	0.16	0.68	0.01	2.19
	WA-WS	3.5			7.89	0.19	0.79	0.02	0.68
	WAA-WS				8.90	0.21	0.69	0.02	2.27
Birch branches	BB	2.8	5.6	3.6	1.34	0.17	0.56	0.01	0.00
	WA-BB	2.9			1.22	0.05	0.30	0.01	0.01
Almond shells	AS	2.6			1.09	0.79	0.40	0.05	0.02
	WA-AS	1.7			0.63	0.12	0.24	0.00	0.01
Mixed stream	WA-MIX				3.17				

[a] Empty cell: not determined. [b] W-WS and WAA-WS digestion before ICP analysis was conducted using HF in combination with HCl, HNO_3 and H_2O_2 to dissolve Si completely. Other samples were digested with HCl, HNO_3 and H_2O_2 only.

Table S4 Inorganic elemental composition of (extracted) feedstocks.

Ppm (dry weight basis)	RG	WA-RG	WS	W-WS[a]	WA-WS	WAA-WS[a]	BB	WA-BB	AS	WA-AS
K	18743	2040	9577	1456	1783	1986	1450	422	7774	1157
Na	487	115	579	95	146	127	298	113	162	32
Ca	3189	2815	4232	4442	5573	4577	3567	2397	2789	1692
Mg	944	378	597	443	551	480	464	315	482	158
Fe	487	115	886	484	726	479	823	119	518	450
Al	25	37	474	815	936	832	47	27	120	28
Cr	108	2	133	4	9	4	9	3	1	2
Mn	21	13	21	17	19	17	1	27	8	5
Ni	86	4	102	2	4	2	131	3	22	6
Zn	13	13	12	12	13	13	87	46	55	4
As	0	2	0	0	0	0	0	0	0	0
B	4	2	2	0	0	1	9	1	0	0
Ba	0	0	0	31	0	33	0	0	0	0
Cd	0	0	0	0	0	0	0	0	0	0
Co	1	0	0	11	0	16	174	2	4	0
Cu	4	2	3	2	2	2	1	1	3	4
Mo	3	3	6	0	1	1	294	0	0	0
Pb	1	4	1	0	4	0	0	4	3	4
S	0	0	0	289	0	301	0	0	0	0
Sb	6	9	4	0	6	0	0	3	0	0
Se	0	0	0	0	0	0	0	0	0	0
Sn	0	0	0	0	0	0	0	0	0	0
Sr	0	0	0	15	0	16	0	0	0	0
Ti	3	2	11	26	29	27	0	1	5	2
V	1	2	1	1	3	1	0	1	0	2
W	0	0	0	162	0	126	0	0	0	0
Ag	1	1	0	0	0	0	1	0	0	0
P	1769	175	409	123	147	182	66	90	543	43
Si	1012	4707	1277	21913	6762	22675	3	49	153	68

[a] W-WS and WAA-WS digestion before ICP analysis was conducted using HF in combination with HCl, HNO_3 and H_2O_2 to dissolve Si completely. Other samples were digested with HCl, HNO_3 and H_2O_2 only.

Table S5 Elemental composition of (pre-extracted) feedstocks.

% w/w (dry weight basis)	Carbon	Hydrogen	Nitrogen	Sulfur	Oxygen	Chloride
RG	45.96	5.61	1.71	0.12	39.32	0.877
WA-RG	46.84	5.69	1.04	0.02	43.13	0.006
WS	44.93	5.55	0.61	0.01	40.97	0.072
WA-WS	44.74	5.47	0.56	0.01	42.11	0.002
BB	50.85	5.94	0.44	0.02	41.26	0.063
WA-BB	49.60	5.79	0.46	0.00	43.30	0.009
AS	50.53	5.65	0.42	0.00	41.56	0.022
WA-AS	50.42	5.76	0.27	0.01	43.08	0.011

Table S6 Extractives sugar content.

% w/w (dry weight basis)[a]		C5 sugars		C6 sugars			
		Xylose	Arabinose	Glucose	Galactose	Fructose	Mannose + Rhamnose
Roadside grass	RG	[b]	0.4	5.3	0.4	1.1	
	WA-RG						
Wheat straw	WS	0.5	0.2	0.9	0.2		
	W-WS			0.2			
	WA-WS	0.2		0.3			
	WAA-WS	0.2		0.2			
	A-WS			0.8			
Birch branches	BB			1.0			
	WA-BB			0.3			
Almond shells	AS	0.1	0.1	0.2	0.1		
	WA-AS	0.1					

[a] Analysed from water extract ASE350 extractions. [b] Empty cell: below detection limit.

Pre-extraction

Experimental

Roadside grass, wheat straw and chipped birch branches were cut to a smaller particle size using a Retsch SM300 cutter mill equipped with a 4 mm sieve. Milled roadside grass, wheat straw, birch branches, almond shells and a mix thereof were pre-extracted using a custom build, software controlled, pre-extraction unit with an effective volume of 14 L. The pre-extraction unit consists of a double walled glass tube (15 cm internal diameter), temperature controlled by a heating circulator (Julabo, 200F). Extraction liquid was added using two piston pumps (Delta 0450) and a mixing chamber. The liquid was preheated using a heat exchanger (Secespol LA14LN-10-3/4”) connected to a separate heating circulator (Julabo, 200F) and sprayed in the top section of the glass extraction tube using a perforated PTFE tubing. Extraction liquid percolated through the biomass bed after which it was cooled in a conical double walled section using a refrigerated circulator (Julabo FL1701).

For herbaceous biomass types, filling the extraction unit with dry biomass causes the particles to stack horizontally upon each other which impaired distribution (channelling) and penetration of the biomass with the aqueous extraction liquid. It was found that pre-wetting of the biomass with water prior to loading the extraction unit caused the pre-soaked particles to have a random orientation which improved the extraction process significantly. Therefore, 2.5 kg feedstock (dry weight) was premixed with 7.5 L demineralised water before loading into the pre-extraction unit (Table S7). Due to the higher density and a lower water absorption capacity of birch branches, 3 kg (dry weight) of feedstock was premixed with 6 L of demineralised water and loaded in the pre-extraction unit resulting in a biomass bed length of 80 cm. The wetted feedstocks were transferred to the pre-extraction unit and preheated at 50 °C for 2 h where the internal biomass bed temperature reached 35 °C. Demineralised water of 48 °C (heated by a heat exchanger) was sprayed onto the top of the biomass bed with a flow rate of 200 mL/min. At the end of the aqueous extraction cycle the average bed temperature reached 46 °C. After water addition, the liquid was allowed to percolate down the biomass bed for 30 min and collected in a vessel. Then, 50% w/w aqueous acetone was added following the same procedure followed by the addition of 10 L of 100% acetone. At the end of the aqueous acetone and pure acetone extraction cycle the average bed temperature reached 47 °C and 43 °C, respectively. The lower bed temperature observed for the pure acetone extraction cycle is likely caused by evaporation of acetone and its recirculation to the biomass bed from the cooler located at the top of the extraction unit.

Almond shells had the highest bulk density and the bed height only reached 34 cm using 3 kg of shells. The combination of a low bed height, high bulk density (limited mass transfer) and fast percolation of extraction liquid through the column could have a negative effect on extraction efficiency. Therefore, it was decided to stepwise soak the shells in extraction liquid followed by draining rather than using percolation to maintain a comparable contact time for all feedstock extractions.
Extractions were conducted as single experiments in this study. However, the solids recovery after duplicate WA-extraction experiments were very similar e.g. 71.8% and 72.2% for WA-RG; 89.3% and 88.3% for WA-WS and 96.9% and 97.2% for WA-AS.

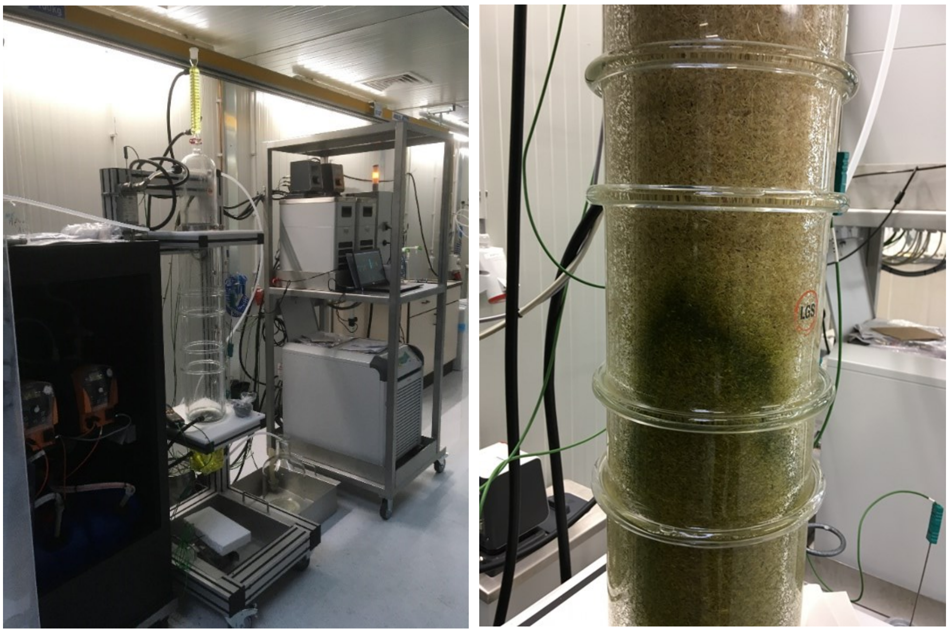

Figure S1 Left: biomass pre-extraction unit, right: pre-extraction of roadside grass (RG)

The extract was collected in three approximately 10 L fractions (F1, F2 and F3). The first and mainly aqueous fraction F1 was sampled and stored frozen. The second and third fraction, F2 and F3 were processed separately in a 20 L rotary evaporator at 60 °C to remove acetone. Precipitate in the extracts was collected by centrifugation at 3488 *g* for 10 min and stored frozen. The remainder of the fractions was further concentrated by rotary evaporation at 60 °C to remove most of the water and to obtain a viscous wet paste that was stored frozen. The extracted feedstocks were dried overnight at 60 °C, weighed and stored for further processing.
The extractives content in the untreated samples and in the samples after pre-extraction was determined using a Dionex Accelerated Solvent Extractor (ASE350) and a temperature of 100 °C as described in the feedstock composition experimental section. Samples were extracted with water, 50% acetone, 100% acetone and 100% pentanone and the extracts processed separately to obtain a fingerprint of hydrophilic and lipophilic extractives content in (larger-scale) pre-extracted and untreated feedstocks.

Table S7 Experimental conditions for biomass pre-extraction.

				Liquid to solid ratio (L solvent/kg dry weight feedstock)					
				Wetting	Extraction				
		Feedstock (kg)	Bed height (cm)	Water	Water	50% acetone	100% acetone	Water	Sum
Roadside grass	WA-RG	2.5	88	3.0	2.5	4.0	4.0	2.5	16.0
Wheat straw	W-WS	2.5	87	3.0	13.0	0.0	0.0	0.0	16.0
	WA-WS	2.5	93	3.0	2.5	4.0	4.0	2.5	16.0
	WAA-WS	2.5	89	2.9	2.5	4.0	10.0	2.5	21.9
	A-WS	2.2	90	0.0	0.0	0.0	12.0 [a]	0.0	12.0
Birch branches	WA-BB	3.0	80	2.0	3.5	4.0	4.0	2.5	16.1
Almond shells	WA-AS	3.0	34	1.7	3.8	4.0	4.0	2.5	16.0
Mixed stream	WA-MIX	3.0	[b]	2.5	3.0	4.0	4.0	2.5	16.0

[a] 95% aqueous acetone instead of 100% acetone. [b] Not determined.

Results

The extracts were collected in three fractions (Figure S2). The first fraction (F1) contained mostly water and extractives and the odour did not indicate significant concentrations of acetone. The second and third fraction (F2, F3) was a mixture of water and acetone. The mass of precipitate collected from the extract after acetone removal in F2 and F3 is low as compared to the total amount of solubilised extractives in each fraction. This does not mean that most of the components are water-soluble as a concentrated milky suspension remained after centrifugation which was not collected as precipitate. This suspended matter can be collected when the extract acidity is lowered to around pH 1. However, concentration to a slurry was selected for these experiments to preserve the extractives.

Table S8 shows that most extractives were found in the water extract F1, except for WA-BB where more extractives were found in F2. Moreover, WA-BB F3 also contained the highest amount of extractives as compared to the other feedstocks.

Figure S3 shows the 50% acetone, 100% acetone and 100% pentanone solvent-soluble extractives content untreated and pre-extracted feedstocks. Most solvent-soluble extractives in the untreated feedstocks are removed using 50% acetone (at a temperature of 50 °C). Consecutive extraction with pure acetone yields a lower amount of extractives. The extraction with pentanone was applied mainly to determine if a significant amount of lipophilic components remained in the sample after the water and acetone extraction. Only for wheat straw these extractives were found in quantities higher than the set detection limit of > 0.1 g / 100 g initial feedstock.

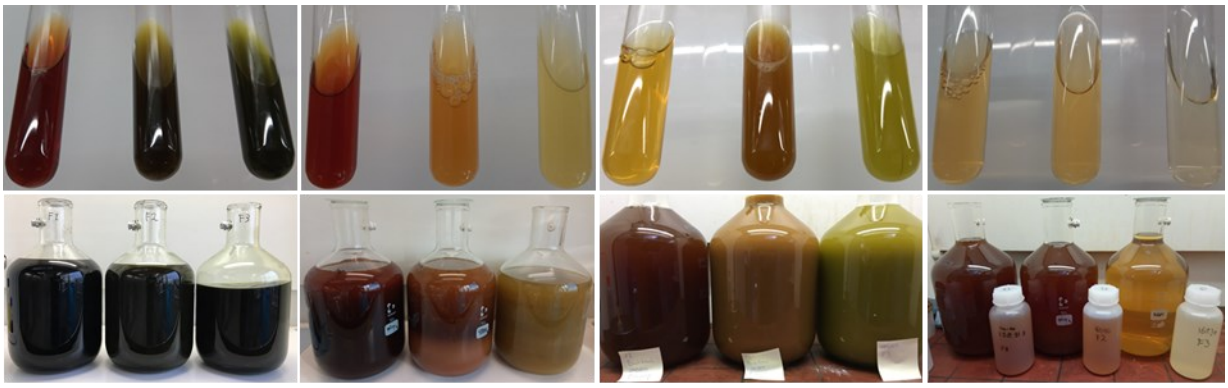

Roadside grass Wheat straw Birch branches Almond shells

Figure S2 Biomass extracts (left: water extract F1, middle: water/acetone extract F2, right: predominantly acetone extract F3).

Table S8 Pre-extraction results.

% w/w		Feedstock recovery[a]	Organic extractives removed[b]	Ash removed	Extractives distribution[c] F1	F2	F3
Roadside grass	WA-RG	71.8	91.0	64.8	77	17	6
Wheat straw	W-WS	88.8	59.4	21.7	85	11	4
	WA-WS	89.3	79.6	15.2	65	24	11
	WAA-WS	88.6	77.9	5.1	67	22	11
	A-WS	96.4	30.9	-9.7	[d]		
Birch branches	WA-BB	92.1	74.3	16.1	31	42	27
Almond shells	WA-AS	96.9	41.5	44.0	69	21	9
Mixed stream	EST-MIX	87.5	71.6	35.0			
	WA-MIX	86.5	77.1	40.1	[d]		

[a] % w/w biomass recovered after extraction. [b] Water and solvent extractives combined. [c] Relative distribution of recovered solids in extracts. [d] Not determined.

Table S9 Mass balance biomass pre-extraction.

		Feedstock (gram dry weight)	Extracted feedstock (gram dry weight)	Water used (ml)[a]	50% acetone used (ml)	100% acetone used (ml)[b]	Extract F1 (gram)	Extract F2 (gram)	Extract F3 (gram)
Roadside grass	WA-RG	2502	1797	19998	10000	10000	10295	9670	9684
Wheat straw	W-WS	2499	2220	40001	0	0	10095	10130	7310
	WA-WS	2506	2237	19998	10000	10000	10205	9565	9053
	WAA-WS	2503	2218	19818	10000	25000	10055	9330	17153
	A-WS	2200	2120	0	0	26400	26400		
Birch branches	WA-BB	2977	2741	23999	12000	12000	11754	11430	10213
Almond shells	WA-AS	2995	2902	24000	12000	12000	11986	11300	15415
Mixed stream	WA-MIX	3049	2638	24393	12196	12196	11908	10989	11513

[a] Total water used for biomass pre-wetting, extraction and final water wash. [b] 95% aqueous acetone was used for A-WS.

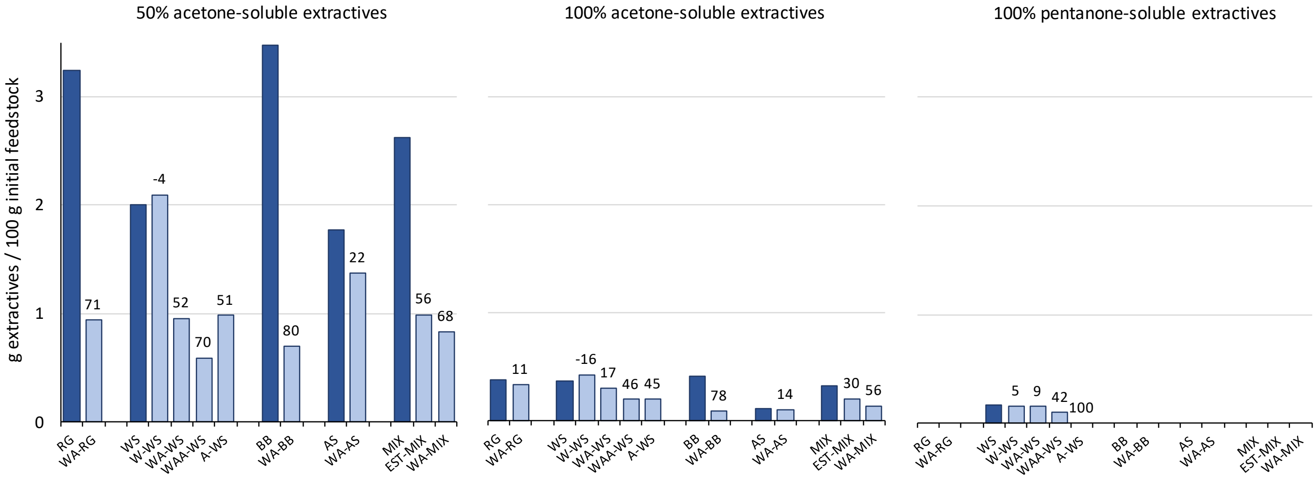

Figure S3 Extractives content of untreated and pre-extracted feedstocks. Labels show the extractives removal by pre-extraction in percentage.

Acid dose screening for fractionation

Experimental

Acid neutralising capacity (ANC) of the milled (pre-extracted) feedstocks was determined by nitric acid titration to pH 1.8 at room temperature for 48 h using a test method (prCEN/TS 15364) developed by the European standardization organization CEN.[9] Small scale organosolv screening experiments were conducted in 125 mL Parr (4748) digestion bombs with Teflon inserts. Untreated and pre-extracted biomass were mixed with 50% w/w aqueous acetone (corrected for the biomass moisture content) and sulfuric acid as catalyst using a liquid-to-solid ratio of 6 L/kg. The weight of pre-extracted biomass was corrected for the solid recovery after extraction to maintain a similar loading of lignocellulose for untreated and pre-extracted biomass. The digestion bombs were heated externally by a heating block set to 142 °C and kept isothermal to achieve approximately 60 min of reaction time at a sample temperature of 140 °C. After cooling to below 25 °C, the slurry was measured for pH and filtered over a Whatman GF/D filter. The solids were washed with 50% w/w aqueous acetone (10 L/kg initial untreated dry biomass). Washed solids were dried in a conventional oven at 60 °C overnight to determine the moisture content of the pulp as well as dry pulp yield. For each feedstock at least six concentrations of sulfuric acid were tested and the results (pH and acid dose) plotted to calculate the required acid dose to obtain a liquor pH of 1.8.

Results

In previous work, the acid dose for fractionation was corrected for the acid neutralising capacity (ANC) of the feedstock to have 35 mM sulfuric acid (70 mN H^+) available for fractionation.[2] For extractives and mineral-rich feedstocks combined with fractionation at a lower liquid-to-solid ratio (6 L /kg instead of 10 L/kg) it was found that the acid dose estimation using ANC correction is not accurate enough to ensure a similar pH during fractionation for untreated and pre-extracted feedstocks. Especially for feedstocks with high extractives content, the ANC based method underestimated the acid dose resulting in too low liquor acidity for optimal fractionation. For an accurate assessment of the effect of biomass pre-extraction on fractionation performance it is crucial that the fractionation experiments are conducted at similar acidity. Therefore, the ANC method as previously reported using water and nitric acid was compared with small scale organosolv screening experiments using a range of acid concentrations (Figure S5).
A series of sulfuric acid concentrations in 50% w/w aqueous acetone were measured for pH and plotted for calculation of the acid concentration needed to obtain a pH 1.8 in the reaction liquid (Figure S4, right). Calculations show that 20 mM sulfuric acid is needed to obtain the target pH which is slightly higher as compared to the 16 mM sulfuric acid for aqueous liquids. The biomass acid neutralising capacity is added to this value to obtain the acid dose for fractionation. Highest ANC values were found for RG with 81 mM additional sulfuric acid needed to compensate for the neutralising capacity. The acid dose requirements are significantly lower for WA-RG. ANC values and subsequent acid dose requirements decline for WS, BB and AS, respectively (including the difference between untreated and pre-extracted feedstocks). Organosolv screening experiments show higher acid dose requirements for fractionation at pH 1.8 as compared to the ANC method.

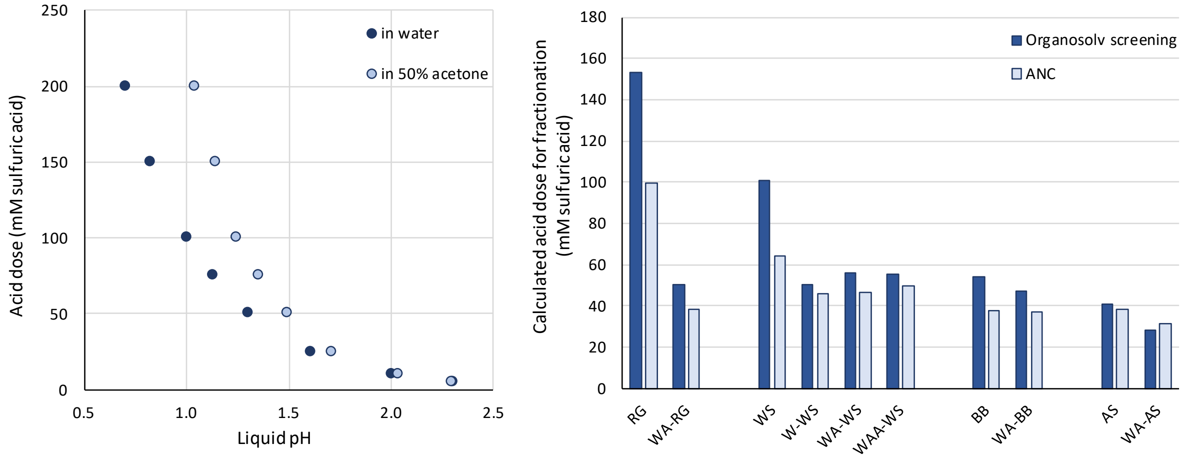

Figure S4 Left: measured pH of a series of sulfuric acid concentrations in water and 50% acetone. Right: estimated required acid dose for organosolv fractionation.

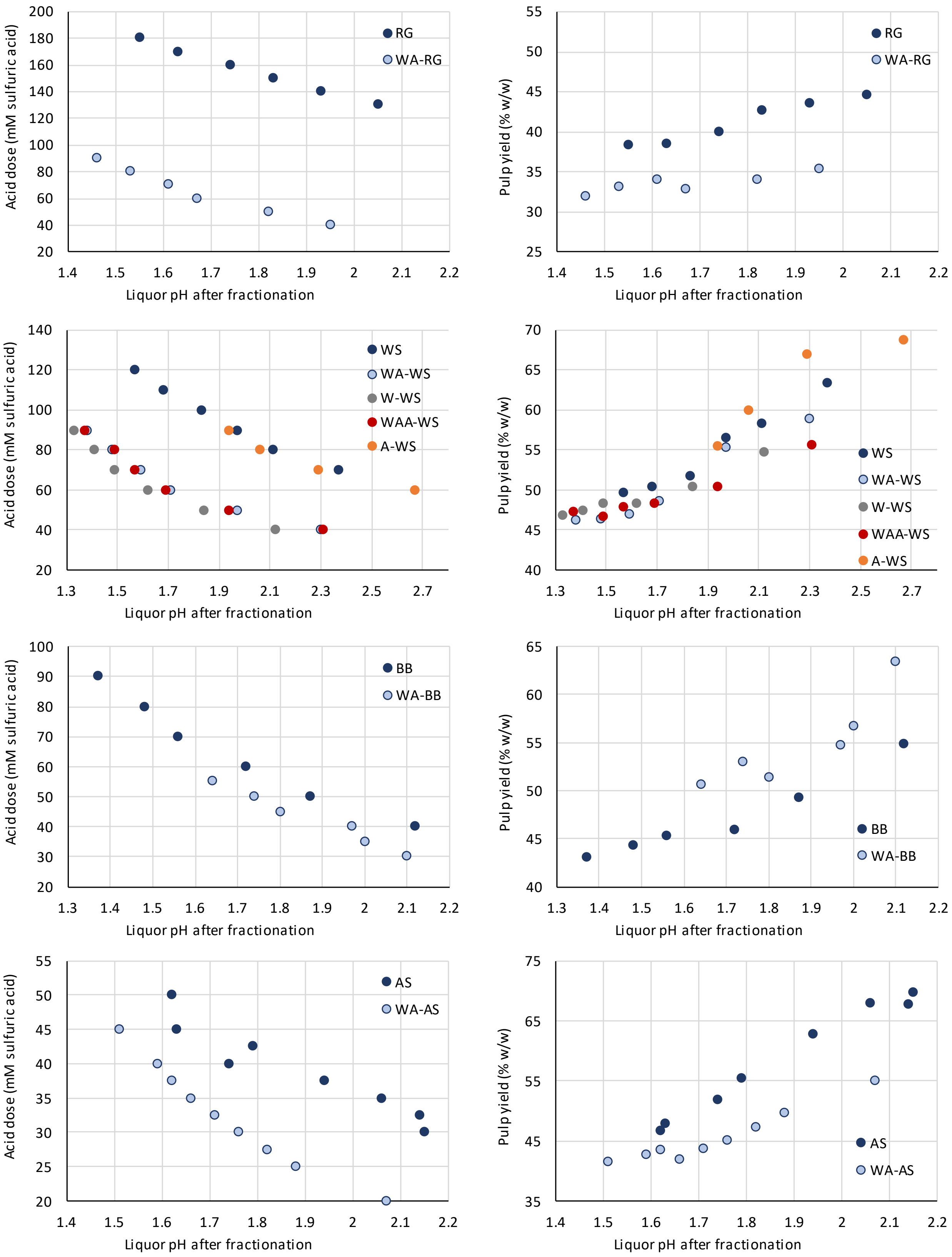

Figure S5 Small scale fractionation screening experiments using a series of acid doses.

Fractionation

Introduction

In previous work fractionation of herbaceous biomass and hardwood was conducted using a liquid to solid (L/S) ratio of 10 and 5 L/kg feedstock respectively.[2] For this study a L/S ratio of 6 L 50% aqueous acetone/kg was applied for all feedstocks. The reaction time was reduced from 120 min to 60 min at 140 °C. Downstream processing is shown in Figure S6 and involves multiple steps to obtain cellulose enriched pulp, hemicellulose hydrolysate and lignin. The design is a balance between adequate processing to obtain high quality fractions and mass balances while maintaining an outlook for cost-efficient processing and scale-up.

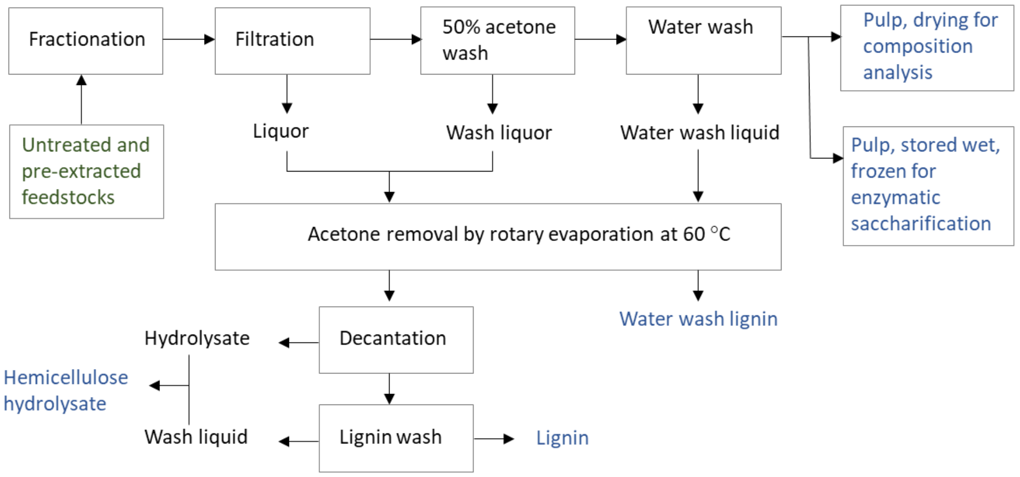

Figure S6 Mild acetone organosolv fractionation and processing of fractions.

Experimental

Fractionation of untreated and pre-extracted feedstocks

Lab-scale pretreatment experiments were performed as single experiments in a 20 L autoclave reactor (Kiloclave, Büchi Glas Uster AG, Switzerland) following a procedure published in earlier work.[2] For untreated feedstocks 2 kg (oven dry weight) was mixed with 12 L 50% w/w aqueous acetone (corrected for the biomass moisture content) to obtain a liquid-to-solid ratio of 6 L/kg. For pre-extracted feedstocks the amount was adjusted for feedstock recovery after extraction. For example, wheat straw recovery after extraction was 89%, the weight used for fractionation was 2000 g * 0.89 = 1785 g which was mixed with 12 L 50% w/w aqueous acetone. This was done to keep a similar lignocellulose loading for untreated and pre-extracted feedstocks. Sulfuric acid (acid dose calculated from the screening experiments) was added as catalyst. The mixture was heated to 140 °C and kept isothermal for 60 min, while stirring with an anchor stirrer at 500 rpm. After cooling to below 25 °C, the slurry was measured for pH and filtered over a Whatman GF/D filter. The solids were first washed with 50% w/w aqueous acetone (3 L/kg initial dry biomass) followed by a wash with water (3 L/kg initial dry biomass) to remove acetone from the pulp. A subsample was dried at 60 °C to determine the moisture content of the pulp as well as dry pulp yield. The remainder of the pulp was stored wet at -20 °C for enzymatic hydrolysis. The filtrate and first wash liquor were combined and samples were taken for analysis. The (wash)liquor and water wash liquid were analysed for monomeric and oligomeric sugars, organic acids, furanics and phenolics. Oligomeric sugars in the (wash)liquor were determined by analysis of total sugars after post-hydrolysis in 1M sulfuric acid at 100 °C for 2 h. Experimentally derived degradation factors for sugar degradation during hydrolysis were used to correct the data.

The (wash)liquor was processed using a 20 L rotary evaporator (Buchi) at 60 °C to remove acetone from the sample. The obtained organosolv hydrolysate + precipitated lignin was kept at room temperature overnight to allow for additional lignin precipitate formation. The liquid was decanted and centrifuged at 3488 *g* for 5 min. Wet lignin precipitate was redissolved in 3 L of 60% w/w aqueous acetone and processed again in the rotary evaporator. After overnight settling the wash liquid was decanted and centrifuged. Hydrolysate and wash liquid were combined and stored at 4 °C. Lignin was dried at 60 °C,

weighed, milled and sieved over a 250 µm mesh sieve. Biochemical composition analysis was conducted on the dry lignin. for the calculation of lignin precipitation yield, the data was corrected for sugars and ash present in the lignin.

Fractionation of birch chips and bark

Birch chips (without bark) were milled twice using a Retsch SM300 cutter mill equipped with a 4 mm sieve and fractionated without pre-extraction using a 2 L autoclave and the methods described for the 20 L experiments. Mixed hardwood bark (birch and poplar) was provided by Sappi. The sample was collected from the debarking unit at the pulp mill in Lanaken. Mixed hardwood bark was milled twice over a 4 mm sieve and pre-extracted using a similar but smaller scale version of the custom build pre-extraction unit. Pre-extraction methods and conditions were identical to the water-acetone (WA-) extraction used for RG, WS, BB and AS. Fractionation was conducted at identical conditions as birch chips in a 2 L autoclave. The required acid dose for fractionation was obtained from small scale organosolv screening experiments with varying sulfuric acid concentrations.

Fractionation for improved delignification

Pre-extracted birch branches and almond shells (WA-BB, WA-AS) were fractionated using a 2 L autoclave and identical process conditions as applied for the 20 L autoclave experiments. However, the reaction time was changed from 60 min to 120 min isothermal at 140 °C. A full mass balance was obtained using the methods described earlier.

Additional experiments were conducted on wet pulp obtained from the 20 L fractionation experiments of WA-BB. For these experiments a 100 ml cell of an ASE350 was filled with 9 g dry weight of wet pulp. The sample was rinsed at 100 °C with 50% w/w aqueous acetone containing either 20, 10 or 5 mM sulfuric acid (ASE350 operation: mode; standard, oven temperature; 100 °C, static time; 10 min, rinse; 100%, purge; 20 sec). The rinsed samples were subsequently fractionated for 60 min in 50% w/w acetone using three different temperature / acid dose combinations (ASE350 operation: mode; standard, oven temperature; 140-180 °C, static time; 60 min, rinse; 100%, purge; 60 sec). The combinations were 140 °C with 20 mM sulfuric acid, 160 °C with 10 mM sulfuric acid and 180 °C with 5 mM sulfuric acid. A sample of the fractionated pulp was dried to determine pulp yield and analysed for biochemical composition. The remaining wet pulp was washed extensively with demineralised water, filtered and stored for pulp enzymatic saccharification screening experiments. The liquor and wash liquor were combined and the lignin precipitated using a rotary evaporator at 60 °C. The precipitated lignin was dried without an additional washing step.

The concentrations of formic acid, acetic acid, levulinic acid, furfural and HMF (5-(hydroxy-methyl)-2-furaldehyde) were determined with High Performance Liquid Chromatography (HPLC, Thermo Scientific Vanquish) equipped with an RI detector, UV detector and a Rezex™ ROA-Organic Acid H+ (8%) LC Column + guard. 5 mM H_2SO_4 was used as eluent (0.60 mL/min) at a column temperature of 65 °C. Only a single analysis was conducted.

Samples were analysed for phenolics using a modified method described in Chen et al.[10] The concentration of phenolics was determined with High Performance Liquid Chromatography (HPLC, Thermo Scientific Vanquish) equipped with a Diode Array Detector at 210, 254 and 280 nm and an Accucore C30 (150 x 4.6 mm, 2.6 µm particle size) LC Column + guard. A gradient of 100% 0.05% trifluoroacetic acid to 90% acetonitrile in 35 min was used as eluent (1.0 mL/min). Only a single analysis was conducted.

Results

Fractionation of untreated and pre-extracted feedstocks

The product distribution after fractionation of untreated and pre-extracted feedstocks is shown in Figure S7 and the general fractionation performance in Table S10. In Figure S7 the hexose, pentose and lignin content of (pre-extracted) feedstocks are set to 100% for each feedstock individually. The obtained pulp and (wash)liquor after fractionation were then analysed and their yield expressed as % w/w of the polymer source they derived from (including a correction for sugar hydration and dehydration during hydrolysis and degradation, respectively). Herein the cellulose-rich solid fraction recovered from the fractionation process is referred to as the pulp. Sugars and their degradation products are grouped as C5 products (arabinose, xylose, furfural) and C6 products (galactose, glucose, mannose, rhamnose, HMF). As the high arabinoxylan and low mannan contents of the feedstocks indicate that glucan is mostly present in the cellulose, the C6 and C5 polymeric sugar distributions largely represent the cellulose fractions and hemicellulose fractions, respectively.

The polymeric C6 sugars were mainly recovered in the pulp, with less than 12% being hydrolysed and lost to the liquor. The solubilised fraction is mainly composed of monomeric sugars and some oligomers. Degradation of C6 sugars to HMF is very minor, except for RG. For example, 0.7% of total C6 sugars degraded to HMF for WS, A-WS and 0.3% for W-, WA- and WAA-

WS. Any levulinic acid formation was below the detection limit for most samples and not included (Table S14). The relatively high values of soluble C6 sugars and HMF seen for RG are related to its extractives sugar content (Table S6). These soluble sugars are immediately released into the liquor during fractionation leading to increased degradation. Other feedstocks showed a low extractives sugar content and pre-extraction removed most of these sugars. In general, biomass pre-extraction did not significantly affect the polymeric C6 product distribution.

As shown in Figure S7B, a considerable amount of the C5 sugars is solubilised, mostly as monomers (see also Table S10). For RG, pre-extraction improved C5 sugar solubilisation from 84% (RG) to 89% (WA-RG) and monomeric sugar yield from 35% to 66%. Consequently, sugar degradation to furfural was significantly reduced (RG, 26% vs WA-RG, 9%). For WS, C5 sugar solubilisation was comparable for WS, WA-WS and WAA-WS, with the slightly lower values for W-WS and A-WS likely resulting from liquor pH. Conversion of polymeric C5 sugars to solubilised monomers improved over the W-WS, WA-WS and WAA-WS series to 69%, 72% and 74%, respectively, as compared to 60% from the fractionation of WS. The degradation of sugars to furfural declined from 15% to 9% for the untreated and pre-extracted WS feed.

WS extracted with 95% acetone (A-WS) retained most of its water-soluble extractives and minerals (Table S1-3); the latter influenced furfural formation which was higher (14%) than with the other extracted WS (9%). BB and AS show comparable results for untreated and pre-extracted feedstocks, showing a high yield of monomeric sugars, limited furfural formation and a larger retention of polymeric C5 sugars in the pulp than with RG and WS.

Many studies have detailed potential pathways for the formation of humins, pseudo-lignin, (oligo)phenolics and organic acids from the degradation and polymerisation/condensation of C5 sugars and their (furanic) derivatives.[11-14] These pathways may include pentose self-condensation as well as condensation reactions of derivatives with lignin fragments and organic extractives. However, such reactions are typical for high severity/temperature treatments and are reported to play a minor role for mild organosolv processes.[15-17] In our case, a closed mass balance is obtained for (WA-) BB and (WA-) AS, which indeed suggests that the formation of such products is limited. Furthermore, an increase in reaction time to 120 min resulted in a minor increase in furfural formation but no loss of sugars to unidentified compounds was observed (Figure S12). The incomplete C5 sugar mass balance for RG and WS, and to a lesser extent WA-RG and WA-WS, does indicate that sugar stability/recovery is to some extent affected by non-lignocellulosic compounds. Xylulose and lyxose, which can be formed by (lewis acid catalysed) isomerisation of xylose [18], may constitute part of the missing sugars in our study. In general, the amount of missing sugars did not seem to directly correlate with, e.g. chloride or potassium content (WA-RG~BB), summative minerals, acid dose or liquor sulfate concentrations (WA-RG~WA-WS~BB), organic extractives, etc.

The lignin product distribution is shown in Figure S7C. Lignin dissolution produces lignin fragments that are heterogeneous in molar mass and molecular structure. Both the precipitated lignin and the residual lignin in the pulp are included in the product distribution. Residual lignin in pulp may be somewhat overestimated as biochemical composition analysis does not differentiate between lignin and water-insoluble non-lignin compounds (extractives and its derivatives). The precipitated lignin fraction was corrected for sugar and ash content but may contain extractives/degradation products that have reacted or co-precipitated with lignin as impurities.
Water-soluble lignin (WSL) and phenolics, two minor categories, were not included in the lignin product distribution. WSL correspond to lignin fragments that do not precipitate after acetone removal from the liquor and thus remain in the hemicellulose hydrolysate. Phenolics (mainly vanillin and syringaldehyde, Table S14) accounted for only 0.1 to 0.3% of the lignin and were therefore not included in the mass balance. All pre-extracted feedstocks showed a larger degree of delignification compared to the untreated feedstocks. It is unclear to what extent this can be attributed to improved fractionation or a lesser extent of (condensed) extractives precipitation onto the pulp.

The most pronounced effect of pre-extraction is seen in the lignin mass balance (Figure S7C), with all untreated samples exceeding 100%. Indeed, more lignin was found in the pulp and as precipitated lignin than was available in the feedstock. The excess of total lignin seen for the WS series correlates directly with a higher lignin content in the pulp, indicating presence of extractives in both pulp and lignin. Lignin mass balance also exceeded 100% for both W-WS and A-WS. This indicates that both water-soluble as well as solvent-soluble extractives may affect lignin and pulp cellulose purity. The lignin yield, defined as the weight of isolated solid precipitate/weight of solubilised lignin *100%, was highest for RG (Table S10). 2 kg of RG contained 262 g lignin from which 93 g “lignin” was retrieved in the pulp and 311 g “lignin” was precipitated from the (wash)liquor bringing the total recovered “lignin” to 404 g (154% total yield in Figure S7C). The total “lignin” yield for WS, BB and AS was 127%, 108% and 102%, respectively, illustrating the feedstock-dependent nature of the amount of co-precipitates.
Notably, untreated feedstock extractives content was more than enough to account for the excess “lignin” in the mass balance. Per fractionation experiment (2 kg initial untreated feedstock) this excess lignin is plotted to the feedstock extractives content in Figure S8 (left). For example, 2 kg of RG contained 441 g of organic extractives and 262 g lignin. This

shows that the pool of extractives is large enough to create the observed lignin impurities. Another potential route for lignin impurity is the formation of pseudo-lignin from the degradation of carbohydrates. C5 sugars not recovered as poly/oligo/monomeric sugars and furfural after fractionation were recalculated to furfural as potential source of pseudo-lignin formation. Figure S8 shows that this can potentially contribute to lignin impurity but cannot account for all the excess lignin recovered after fractionation. An interesting study from Bauer et al. detailed the effect of different steam explosion conditions on hay composition and digestibility. Under the mildest conditions (160 °C, 5-10 min) pseudo-lignin appears to be primarily formed from extractives degradation and condensation. Only at higher process severities (190-220 °C) sugar degradation led to significant pseudo-lignin formation.[19]

For all the water-acetone pre-extracted feedstocks, total lignin mass balance was between 94% and 99% except for BB which showed a lower yield of 89%. The mixed stream (WA-MIX) showed a slightly higher amount of lignin in the pulp (i.e. lower delignification) then the value expected from the separate feedstocks (EST-MIX). The pulps from (pre-extracted) BB and AS retained higher amounts of polymeric C5 sugars and lignin as compared to the herbaceous biomass, RG and WS.
To recalculate the hexose (C6) and pentose (C5) sugar data to cellulose and hemicellulose sugars, several assumptions were made as the biochemical composition analysis does not differentiate between glucose present in the cellulose and glucose present in the hemicellulose fraction:

- Glucan is only present in the hemicellulose as glucomannan in equimolar quantities.
- For every molecule of mannan present in the hemicellulose, one glucose is added to the hemicellulose fraction and deducted from the cellulose fraction.
- No oligomeric mannan was detected in the liquor, therefore no hemicellulose glucan is present as oligomer.
- The weight percent hydrolysis and conversion of mannan to monomeric sugars is also applied to hemicellulose glucan.

Contrary to the product distributions and yields in the publication, the extractives sugar contents (Table S6) are excluded from the calculations. The results are shown in Table S11. Cellulose recovery is higher than the C6 sugar recovery because the more labile / less crystalline hemicellulose glucan, galactan, mannan and rhamnan are removed from the calculations. Only minor differences are observed when comparing C5 to monomeric sugars and hemicellulose to monomeric sugars because of the low mannan, galactan and rhamnan content of the selected feedstocks.

Table S10 General fractionation performance.

% w/w		Pulp yield[a]	C6 recovery[b]	C5 solubilisation[c]	Delignification[d]	Lignin yield[e]
Roadside grass	RG	39.6	78.1	84.4	64.5	184.6
	WA-RG	34.5	94.0	89.1	76.7	93.6
Wheat straw	WS	49.5	92.4	90.0	66.5	140.8
	W-WS	48.7	95.6	86.9	70.7	110.4
	WA-WS	48.7	97.3	88.6	76.5	92.7
	WAA-WS	46.8	93.8	88.4	80.9	94.2
	A-WS	51.0	90.6	85.8	74.3	106.7
Birch branches	BB	45.9	88.8	80.0	58.5	113.9
	WA-BB	45.4	92.2	79.4	63.3	82.8
Almond shells	AS	42.7	94.5	74.3	68.6	103.7
	WA-AS	40.5	95.9	77.7	72.4	98.4
Mixed stream	EST-MIX	42.3	94.9	83.7	72.2	91.9
	WA-MIX	39.1	89.4	87.2	66.6	100.0

[a] Pulp yield calculated from untreated feedstocks (including pre-extraction solid recovery). [b] Percentage of feedstock glucan, mannan, galactan and rhamnan recovered in the solid pulp. [c] Percentage of feedstock xylan and arabinan not recovered in the solid pulp. [d] Percentage of feedstock lignin not recovered in the pulp. [e] Percentage of solubilised lignin obtained as solid after precipitation from liquor.

Table S11 Product distribution of hexoses and pentoses.

% w/w	Polymeric C6 sugar recovery	Polymeric C5 to oligomeric sugars	Polymeric C5 to monomeric sugars	Cellulose recovery	Hemicellulose to oligomeric sugars	Hemicellulose to monomeric sugars
RG	96	4	35	98	3	39
WA-RG	94	3	66	96	3	67
WS	95	3	62	97	3	63
W-WS	96	4	69	100	3	73
WA-WS	98	2	72	101	4	69
WAA-WS	94	3	75	99	3	74
A-WS	93	8	54	95	7	55
BB	92	0	77	100	1	76
WA-BB	93	1	78	102	2	76
AS	96	5	67	101	4	68
WA-AS	96	3	74	101	3	74
WA-MIX	89	3	72	92	3	76

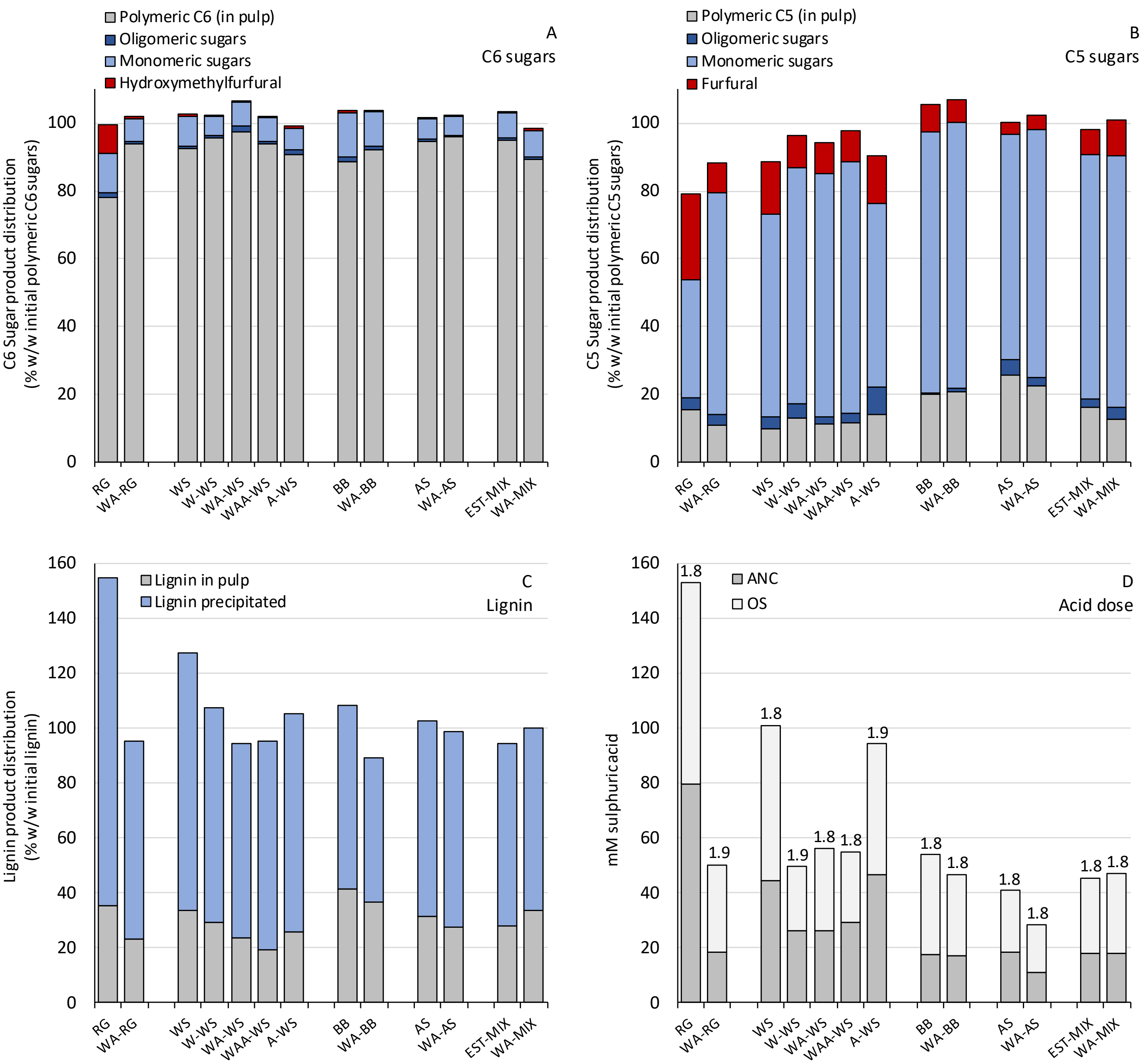

Figure S7 Product distribution of C6 sugars (A), C5 sugars (B) and lignin (C) after fractionation of (extracted) feedstocks, expressed as % w/w of the polymer source converted to the specific product. Fig D: (extracted) feedstock acid neutralising capacity (ANC) and applied total acid dose for fractionation as determined by small scale screening experiments (OS = total acid dose – ANC). Labels represent the liquor pH after fractionation.

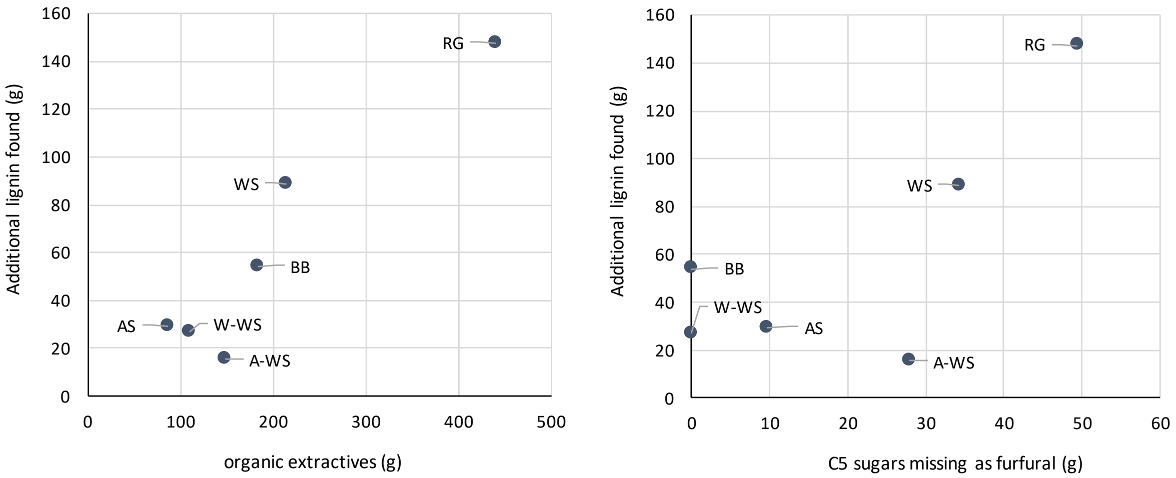

Figure S8 Correlation of excess lignin found per fractionation experiment (lignin isolated + pulp lignin – feedstock lignin) with feedstock extractives content (left) and potential pseudo-lignin formation (right).

Table S12 Mass of solids and liquids.

Gram		(Extr.) feedstock	Pulp	Precipitated (wash)liquor lignin[a]	Pulp waterwash lignin[a]	Combined (wash)liquor	Waterwash[b]	Hydrolysate[c]
Roadside grass	RG	2000	792	294	23	11572	4930	6413
	WA-RG	1436	690	159	12	11149	3945	6141
Wheat straw	WS	2000	991	278	12	11381	4150	6397
	W-WS	1776	974	212	29	10687	4428	5854
	WA-WS	1785	973	199	17	10635	4417	6044
	WAA-WS	1772	935	214	15	11087	4408	6065
	A-WS	1927	1021	224	26	11471	3526	6457
Birch branches	BB	2000	918	371	7	12537	4857	6576
	WA-BB	1804	889	264	17	12466	4516	6722
Almond shells	AS	2000	854	420	1	14477	5017	7313
	WA-AS	1937	810	413	1	14465	4930	7528
Mixed stream	WA-MIX	1730	782	272	4	12766	4619	7243

[a] Not corrected for sugar and ash content. [b] After acetone removal. [c] Combined hydrolysate and lignin wash liquid after acetone removal.

Table S13 Liquor and hydrolysate sugar composition.

	Monomeric sugars (mg/kg)						Oligomeric (mg/kg)	
	Arabinose	Xylose	Mannose	Galactose	Glucose	Rhamnose	C5 sugars	C6 sugars
(Wash) liquor								
RG	2204	9069	[a]	1393	4562	145	1140	733
WA-RG	3257	19490		1153	1661	275	1034	331
WS	2989	22937		1066	4163	198	1321	566
W-WS	2773	22824		814	2330	169	1674	510
WA-WS	3058	25281		851	3103	218	844	1059
WAA-WS	3083	25691		888	3197	221	1109	511
A-WS	2470	18633	237	829	2848		3146	1015
BB	1544	20668	649	1677	3660	762	640	124
WA-BB	1605	20166		1647	2607	766	451	316
AS	1154	25758		1127	566	533	1647	303
WA-AS	1139	27918		1138	363	583	974	153
WA-MIX	2113	23551		1180	2190	432	1048	327
Pulp waterwash								
RG	590	2719		372	1404	46	ND	ND
WA-RG	1094	6561		396	536	91	ND	ND
WS	730	5369	111	280	1073	41	ND	ND
W-WS	1414	11248		429	1198	82	ND	ND
WA-WS	1304	10501		390	1420	100	ND	ND
WAA-WS	813	6556		242	834	44	ND	ND
A-WS	1454	10241	157	507	1666		ND	ND
BB	195	2354	131	219	484	85	ND	ND
WA-BB	263	3167		262	429	123	ND	ND
AS	ND	ND	ND	ND	ND	ND	ND	ND
WA-AS	53	1337		47	19	27	ND	ND
WA-MIX	227	2437		133	232	40	ND	ND
Hydrolysate								
RG	3398	15757		2165	7455	248	ND	ND
WA-RG	5663	36349		2322	2750	554	ND	ND
WS	5148	38970		1850	7317	338	ND	ND
W-WS	4602	38109		1378	4049	258	ND	ND
WA-WS	4999	42904		1617	4989	411	ND	ND
WAA-WS	5413	44633		1580	5703	299	ND	ND
A-WS	ND	ND	ND	ND	ND	ND	ND	ND
BB	2794	36662		2936	6331	1328	ND	ND
WA-BB	2728	35248		2920	4262	1333	ND	ND
AS	2177	47137		2232	1082	1050	ND	ND
WA-AS	2109	50542		2157	743	1089	ND	ND
WA-MIX	3601	41003		1978	3486	765	ND	ND

[a] Empty cell: below detection limit. ND Not determined.

Table S14 Liquor and hydrolysate oxygenate composition.

	Organic acids (mg/kg)			Furanics (mg/kg)		Phenolics (mg/kg)	
	Formic	Acetic	Levulinic	Furfural	HMF	Vanillin	Syringaldehyde
(Wash) liquor							
RG	732	3673	[a]	5593	3055	23	18
WA-RG	485	3988		2067	214	24	9
WS	577	2901	104	4458	330	38	26
W-WS	318	2063		2472	126	27	13
WA-WS	328	2419		2448	118	32	17
WAA-WS	306	2167		2411	103	28	15
A-WS	344	2408	55	3809	289	29	37
BB	945	7581	29	1536	217	27	30
WA-BB	686	7348		1247	100	31	46
AS	680	7861		877	91	94	17
WA-AS	608	8697		1085	37	83	28
WA-MIX	549	5753		2316	142	ND	ND
Pulp waterwash							
RG	233	984	53	933	845	6	4
WA-RG	136	1061		317	71	7	2
WS	115	495		474	61	7	5
W-WS	145	886		677	49	8	10
WA-WS	109	784		487	46	11	13
WAA-WS	65	506		348	51	14	15
A-WS	174	1103		1071	119	13	17
BB	99	700		93	32	3	4
WA-BB	95	987		110	14	5	29
AS	ND	ND	ND	ND	ND	ND	ND
WA-AS	17	139		14		3	3
WA-MIX		393		149	56	ND	ND
Hydrolysate							
RG	1478	6440	394	4946	5367	27	27
WA-RG	752	6683		2078	357	31	13
WS	896	4660	160	4102	539	44	37
W-WS	514	3514	38	2791	222	43	24
WA-WS	475	3834		2647	178	40	24
WAA-WS	513	3794	29	2782	219	44	24
A-WS	ND	ND	ND	ND	ND	ND	ND
BB	1460	13029	95	1531	360	39	32
WA-BB	1053	12849		1374	147	53	61
AS	1246	15728		999	140	187	37
WA-AS	1042	16383		1364	54	149	39
WA-MIX	824	9592	132	2820	237	ND	ND

[a] Empty cell: below detection limit. [ND] Not determined.

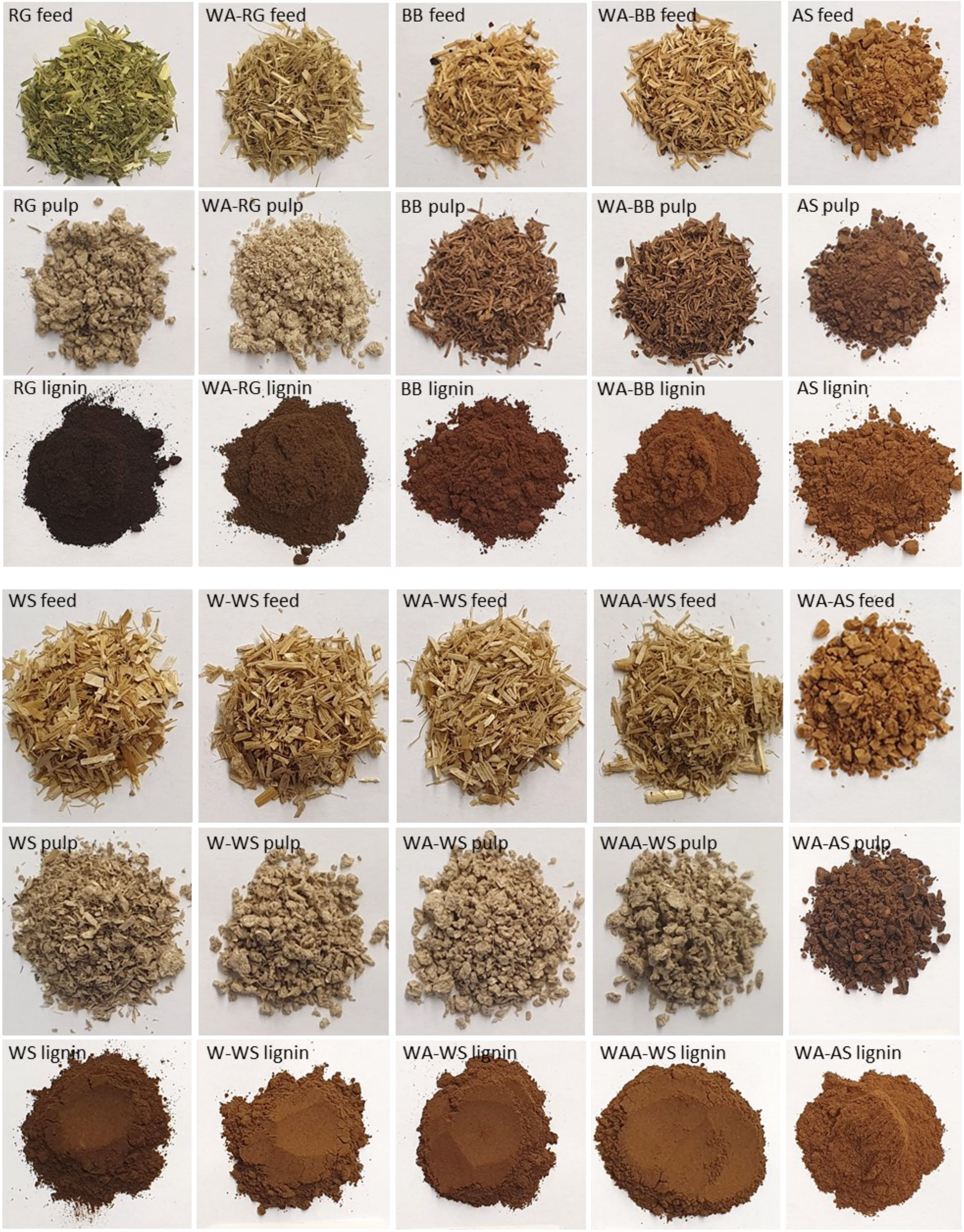

Figure S9 Pictures of feedstock, pulp and precipitated lignin.

Fractionation of birch chips and bark

Fractionation of (pre-extracted) birch branches (BB and WA-BB) showed lower delignification than observed for clean birch wood in previous work.[2] Therefore, the composition and fractionation performance of clean birch wood chips (BC), pre-extracted birch branches (WA-BB) and pre-extracted mixed hardwood bark (WA-HB) were compared to assess how the presence of bark affects the fractionation results.

Birch stem wood is relatively rich in C6 sugars (predominantly glucan) and C5 sugars (predominantly arabinoxylan) as shown in Figure S10. The feedstock sugar content decreases and lignin / ash content increases for BC, WA-BB and WA-HB, respectively.

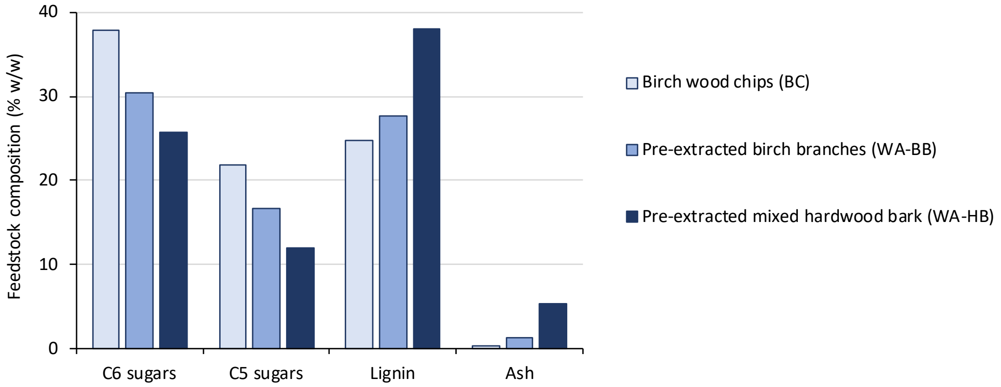

Figure S10 Birch wood chips, pre-extracted birch branches and pre-extracted hardwood bark composition

Fractionation was conducted using an identical approach as described in the previous section. Liquor pH after fractionation was comparable for BC and WA-BB (pH 1.8) but somewhat higher for WA-HB (pH 2.0). The pH difference is expected to have a minor impact on fractionation results. Pre-extraction of HB removed 15.5% of the feedstock weight as organic extractives which is more than the 7.9% extracted from BB. The fractionation product distribution is shown in Figure S11. Contrary to the mass balances in the previous section, the data for BC and WA-HB is not corrected for water-soluble sugars in the extracts and lignin sugar / ash content. This is expected to have a minor impact on fractionation results. The C6 sugar product distribution shows an increase in polymeric (pulp) sugar recovery for WA-BB and WA-HB. Typically, the biochemical composition analysis does not show such deviations and is possibly caused by interference from a co-eluting compound during sugar analysis. The C5 sugar product distribution of BC shows similar C5 sugar solubilisation as compared to WA-BB but produced more oligomers and less furfural. Reduced C5 sugar solubilisation as found for WA-HB could result from the slightly higher pH during processing. A relatively low monomeric sugar yield and furfural formation was observed and confirmed by repeated analysis.

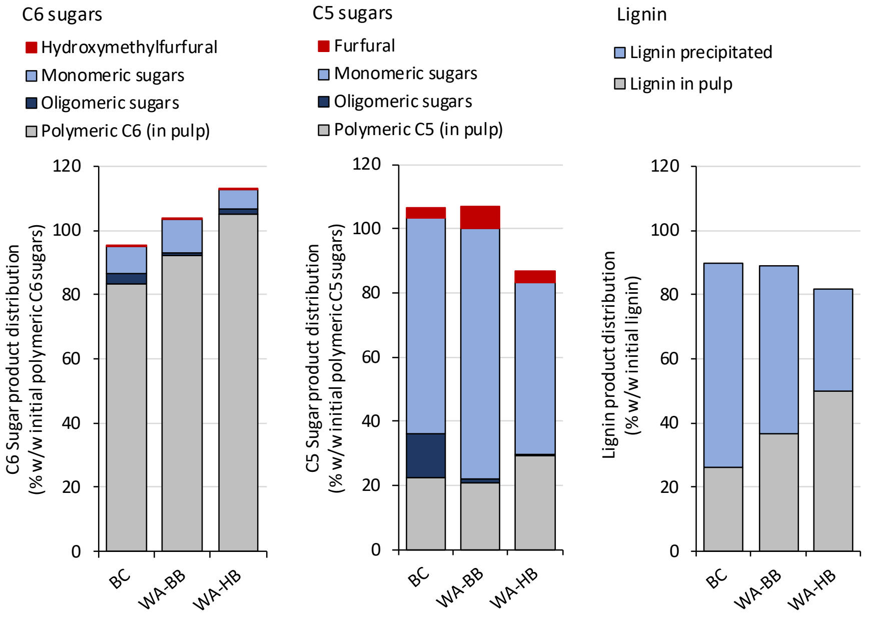

Figure S11 Birch, birch branches and hardwood bark fractionation results

The lignin product distribution clearly shows reduced feedstock delignification for WA-BB and especially for WA-HB. This indicates increased recalcitrance of a part of the lignin for mild acetone organosolv fractionation. The results are in accordance with studies into the structural characteristics of wood and bark lignin revealing a higher degree of condensation and a lower abundance of β-aryl ether linkages in bark lignin as compared to the stemwood lignin.[20-21]

Fractionation for improved delignification

The effect of increased reaction time on the fractionation performance of WA-BB and WA-AS was determined. Figure S12 shows the differences in product distribution when the reaction time is increased from 60 min to 120 min. The increase in reaction time provides surprisingly limited improvements in fractionation performance. Polymeric C6 sugar recovery decreased slightly from 92.2% to 89.8% for WA-BB and from 95.9% to 93.2% for WA-AS. A somewhat larger effect is observed for polymeric C5 hydrolysis which increased from 79.4% to 85.9% for WA-BB and from 77.7% to 83.0% for WA-AS. Delignification did not improve significantly for both WA-BB and WA-AS which increased from 63.3% to 65.3% and 72.4% to 75.8% respectively. Limited downside effects on HMF and furfural formation are observed.

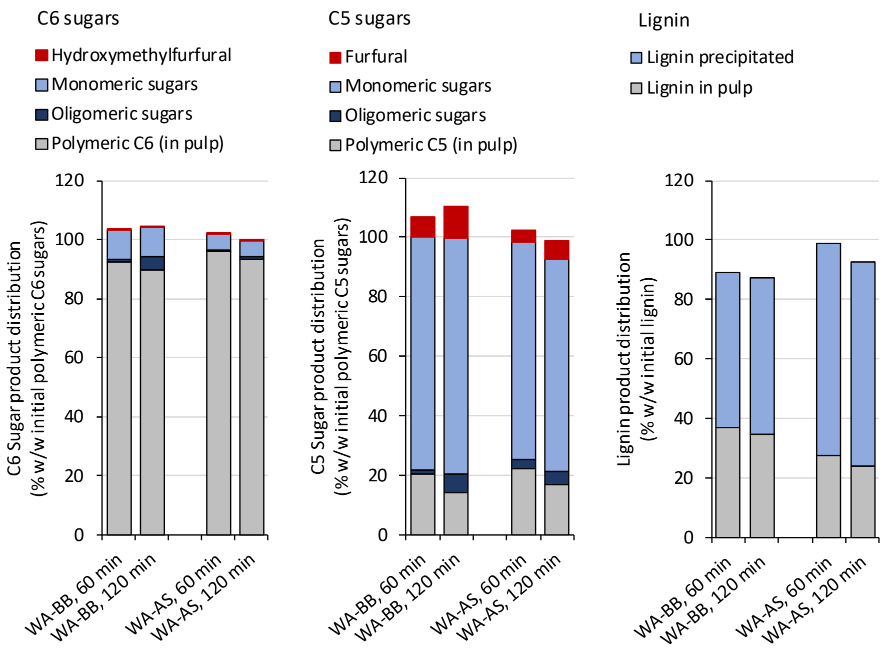

Figure S12 Product distribution for 60 and 120 min fractionation of pre-extracted birch branches and almond shells

As observed for BB and AS, mild acetone organosolv process severity is too low for achieving high delignification when processing feedstocks with increased recalcitrant lignin content. For improved delignification of BB a series of pulp post-treatment experiments were conducted to explore the potential for additional delignification without negatively affecting the organosolv process characteristics. Wet BB pulp was post-treated for 60 min at 140 °C, 160 °C and 180 °C with adjusted sulfuric acid doses (Table S15).

Table S15, Table S16 and Figure S13 shows the composition of BB pulp and the effect of post treatment at different processing regimes (i.e. 140 °C/20mM, 160 °C/10mM and 180 °C/5mM). For Figure S13 the results of post-processing are added to the mass balance of the fractionation of BB to present the mass balance of fractionation + post-processing. The results show that post-processing at 140 °C provides some improvement in polymeric C5 sugar hydrolysis and delignification. Post treatment of BB pulp for 60 min at 140 °C resulted in a higher overall delignification (69.6%) as compared to the 120 min fractionation experiment with WA-BB in Figure S12 (65.3%). The BB pulp post treatment experiments were conducted in an ASE350 where the pulp is washed at process temperature (140 - 180 °C), this might explain the difference in observed delignification as the larger-scale autoclave experiments employ pulp washing at room temperature. Pulp post-processing at 160 °C improves polymeric C5 sugar hydrolysis but does not result in increased delignification. Pulp post-processing at 180 °C completes polymeric C5 sugar hydrolysis and further improves delignification. However, the polymeric C6 sugar recovery shows a significant decline due to hydrolysis of the (crystalline) cellulose. Overall, this exploratory study indicates that high levels of BB delignification are difficult to achieve using organosolv type processes.

Table S15 Composition of (post-treated) birch branches pulp.

% w/w (dry weight basis)		Xylan	Arabinan	Glucan	Galactan	Mannan	Rhamnan	Lignin
BB pulp		7.2	[a]	57.5		1.4		25.0
Pulp treatment	140 °C, 20 mM	5.7		67.4				20.4
	160 °C, 10 mM	2.7		68.4				20.4
	180 °C, 5 mM			71.0				20.9

[a] Empty cell, below detection limit

Table S16 Results of birch branches pulp post-treatment.

% w/w (dry weight basis)			Removed from pulp after post treatment		
		Pulp yield	Glucan	Xylan	Lignin
Pulp treatment	140 °C, 20 mM	90.8	-6.4	28.4	26.9
	160 °C, 10 mM	89.9	-6.9	66.4	27.4
	180 °C, 5 mM	70.1	13.5	100	42.1

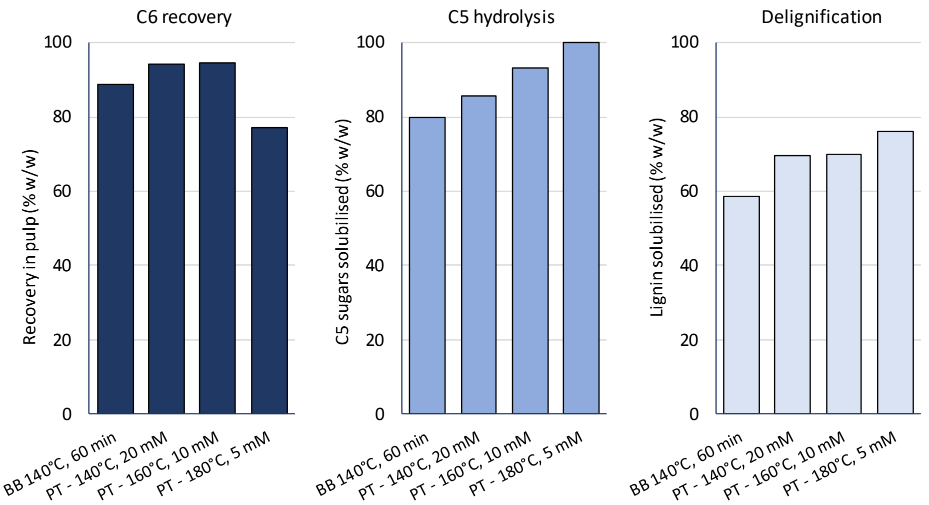

Figure S13 Combined results of fractionation of pre-extracted birch branches and pulp post-treatment

Pulp

Experimental

Dried pulp samples were analysed for biochemical composition (as described for the feedstocks composition analysis). Samples were not pre-extracted in the ASE350 before composition analysis because of the uncertainty in lignin and sugar extraction / quantification. Separate pulp samples were pre-extracted using an ASE350 and the previously described modified extraction protocol with water, 50% acetone, 100% acetone and pentanone. 1 mL extract was diluted to obtain 2 ml extract in 50% acetone. The mixture is further diluted with 98 mL 75% aqueous ethanol and its absorbance measured at 205 nm. An identical solvent mixture was used as blank and to redissolve mild acetone organosolv beech lignin as standard. The remainder of the extract was dried and weighed. Quantified lignin in the extract was subtracted from the total dry weight of extract solids.

For pulp enzymatic saccharification 5 g (dry weight) of wet pretreated material, 50 mL liquid containing 0.05 M sodium citrate buffer pH 5.0 and 0.02% w/v sodium azide (corrected for moisture in sample and enzyme dose) was mixed according to NREL/TP-510-42629. Enzymatic hydrolysis was conducted for 72 h using an IKA ks4000 rotary shaker at 50 °C and 140 rpm. The enzyme mixture MetZyme® SUNO™ 036 (www.metgen.com) was used for (hemi)cellulose hydrolysis. An enzyme dose of 0.15 g enzyme mixture solution / g pulp glucan (15%) was used for all experiments. Additionally, a higher enzyme dose was applied of 0.50 g enzyme mixture solution / g pulp glucan (50%). After 0, 6, 24, 48 and 72 h samples were taken and analysed for monomeric sugar content using HPAEC-PAD. Samples from the experiments with a 50% enzyme dose were analysed using colorimetric determination of the glucose concentration. In short, 2 mL of reagent (9% v/v o-toluidine, 1.5% w/v thiourea in glacial acetic acid) was added to 20 µL of (diluted) sample and heated in a water bath at 90 °C for 8 min. After cooling in tap water for 4 min, the absorbance was measured at 635 nm. Glucose yield is expressed as weight percentage of the pulp glucan converted to monomeric glucose.

Thermal desorption GC/MS was performed on 20 mg of sample. TD was carried out by the use of a GERSTEL Thermal Desorption Unit (TDU2) connected to a Agilent GC 7890A + MSD 5975C via a GERSTEL CIS4 (PTV injector) that was used as a cryo trap. The TD tube was rapidly heated to 350 °C for desorption under helium flow of 30 mL/min. Released volatiles were trapped on the CIS4 liner that was cooled to -150 °C after splitting 1:30. After 1 min of desorption, the TDU was cooled to 35 °C, and the CIS4 was heated to 250 °C transferring the volatiles to the GC column. The GC was equipped with a Zebron ZB-WAXplus, dimensions: 30 cm x 0.25 mm, film thickness 0.25 µm. The GC oven program was: Initial at 50 °C for 5 min, 10 °C/min to 250 °C then 35 min at 250 °C. The MS was operated in the scan acquisition mode (29–350 amu). The MS source and MS quadruple temperatures were 250 °C and 150 °C respectively while the transfer line temperature was 220 °C. The identification of components was done using the NIST library (2008).

Results

Cellulose enriched pulp obtained from mild acetone organosolv fractionation of lignocellulosic biomass can be valorised via multiple routes. For material applications it has potential for direct application in, for example, board materials or after mechanical disintegration and bleaching of the pulp to microcrystalline cellulose (MCC) or cellulose nanofibrils (CNF). Alternatively, cellulose enriched pulp can be enzymatically hydrolysed to monomeric sugars which can be converted by chemo-catalytic and fermentative processes to chemical building blocks, fuels and solvents. In this section we present the pulp lignocellulose and extractives composition and enzymatic pulp saccharification to monomeric sugars. The effects of biomass pre-extraction, through fractionation, on pulp composition and saccharification are discussed.

Table S17 Biochemical composition of pulps.

% w/w (dry weight basis)		Polymeric C5 sugars		Polymeric C6 sugars					
		Xylan	Arabinan	Glucan	Galactan	Mannan	Rhamnan	Lignin	Ash
Roadside grass	RG	7.3	[a]	60.8				11.7	9.2
	WA-RG	6.0		71.6				7.9	6.5
Wheat straw	WS	4.7		65.4				10.3	13.2
	W-WS	5.6		66.4				9.1	13.1
	WA-WS	5.1		66.1				7.2	14.2
	WAA-WS	5.2		67.5				6.0	14.3
	A-WS	6.4		64.9	0.3	0.4		7.8	18.8
Birch branches	BB	7.2		57.5		1.4		25.0	2.4
	WA-BB	7.4		59.9		1.2		21.7	2.2
Almond shells	AS	15.5		55.4				21.1	0.5
	WA-AS	14.1		59.0				19.5	0.2
Mixed stream	WA-MIX	6.6		66.5				17.5	5.4

[a] Empty cell, below detection limit.

Table S18 Inorganic elemental composition of pulps.

Ppm (dry weight basis)	RG	WA-RG	WS	W-WS[a]	WA-WS	WAA-WS[a]	BB	WA-BB	AS	WA-AS
K	7372	197	254	688	723	833	148	191	2806	233
Na	41	158	104	144	169	166	26	198	100	210
Ca	8741	3258	5055	6848	8879	8934	1412	5329	6716	2576
Mg	1291	31	19	180	182	185	15	51	206	95
Fe	803	117	50	399	429	354	165	52	1232	112
Al	35	37	41	1455	1025	1502	91	35	665	81
Cr	21	27	4	56	28	8	8	2	84	0
Mn	50	3	3	5	7	5	1	3	38	2
Ni	89	19	2	16	25	3	3	9	104	12
Zn	20	7	5	5	11	4	1	10	14	10
As	3	2	0	0	0	0	0	0	0	0
B	1	0	2	1	0	1	1	0	0	0
Ba	0	0	0	62	0	68	0	0	0	0
Cd	0	0	0	0	0	0	0	0	0	0
Co	2	0	0	7	2	7	1	0	3	0
Cu	5	5	3	3	10	4	19	7	5	9
Mo	10	9	12	24	8	3	39	2	34	0
Pb	0	0	0	0	0	0	0	3	0	4
S	0	0	0	5958	0	7393	0	0	0	0
Sb	0	0	0	0	6	0	0	3	0	4
Se	0	0	0	0	0	0	0	0	0	0
Sn	0	0	0	0	0	0	0	0	0	0
Sr	0	0	0	23	0	30	0	0	0	0
Ti	4	10	5	52	27	49	13	6	40	4
V	1	3	0	2	4	3	0	4	2	3
W	0	0	0	80	0	85	0	0	0	0
Ag	0	0	1	0	0	0	0	0	0	0
P	48	65	39	53	105	65	89	47	72	87
Si	5068	4281	99	42526	4604	46564	137	123	8782	158

[a] W-WS and WAA-WS digestion before ICP analysis was conducted using HF in combination with HCl, HNO_3 and H_2O_2 to dissolve Si completely, other samples were digested with HCl, HNO_3 and H_2O_2 only.

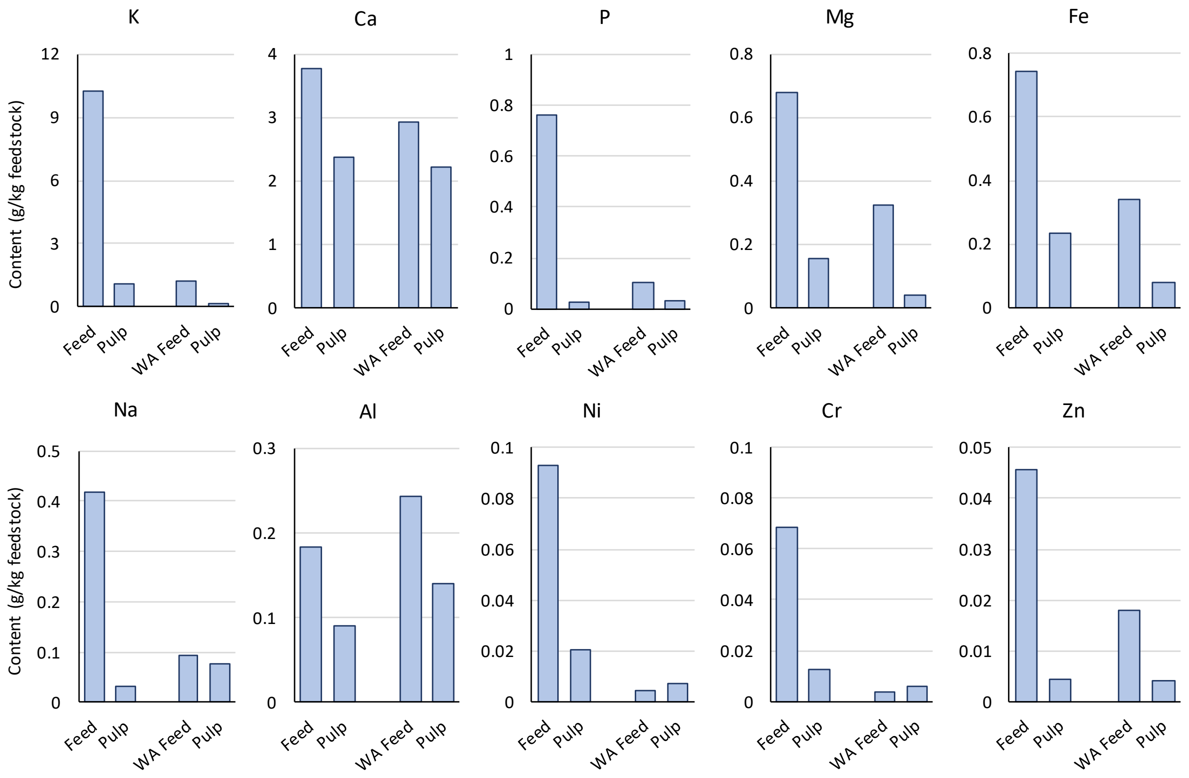

Figure S14 Mineral content of (pre-extracted) feedstock and pulp per fractionation of 1 kg feedstock. Average values for (WA)- RG, WS, BB and AS are reported.

Pulp extractives content was determined using water, acetone and pentanone extraction in an ASE350. Such an approach is challenging due to potential co-extraction of lignocellulose components such as lignin and sugars. Figure S15 shows the pulp extractives content where extract lignin content was quantified by UV measurements. Extract sugar content was not determined and can comprise a significant part of the extractives content, especially for the water-extractives. The 50% acetone extract contains most of the lignin and more lignin is detected in the higher density feedstocks BB and AS as compared to RG and WS. Possibly this is a result of incomplete washing of the pulp. Uncertainties in the extent of pulp sugar extraction and extract lignin quantification does not allow precise quantification of non-lignocellulose components. However, untreated feedstocks (and W-WS) do consistently show a higher amount of extractives.

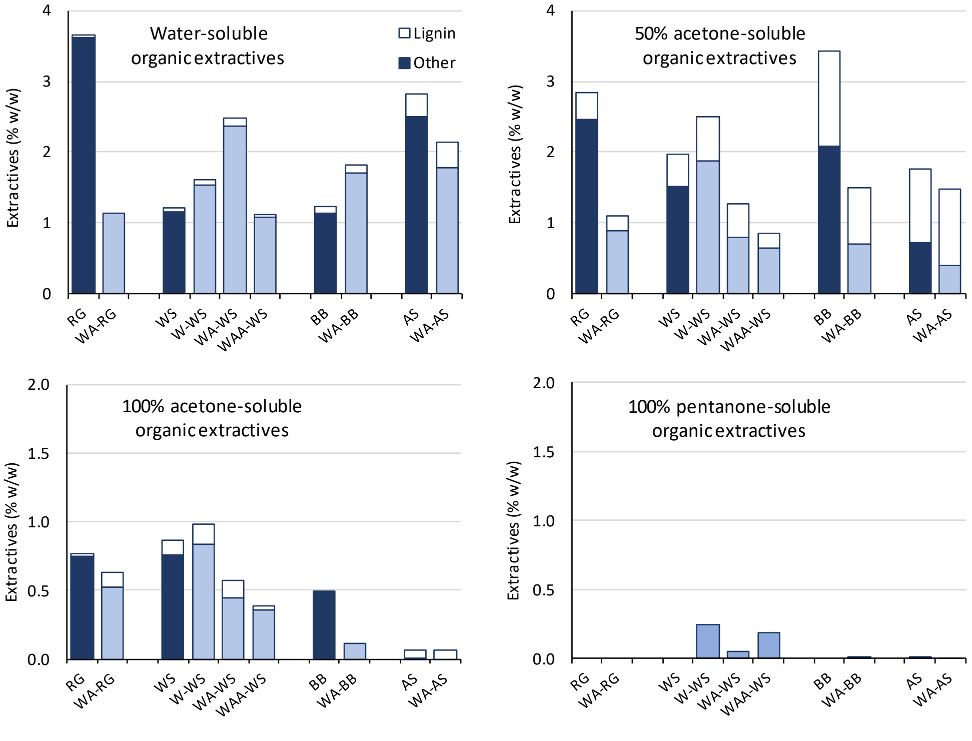

Figure S15 Pulp extractives content.

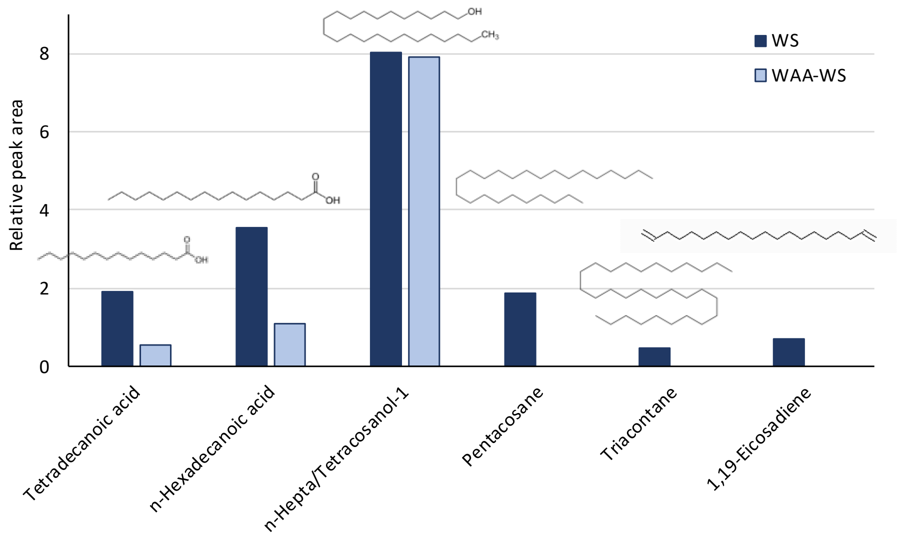

Figure S16 TD-GC/MS results of wheat straw (WS) and pre-extracted wheat straw (WAA-WS) pulps.

Pulp enzymatic saccharification

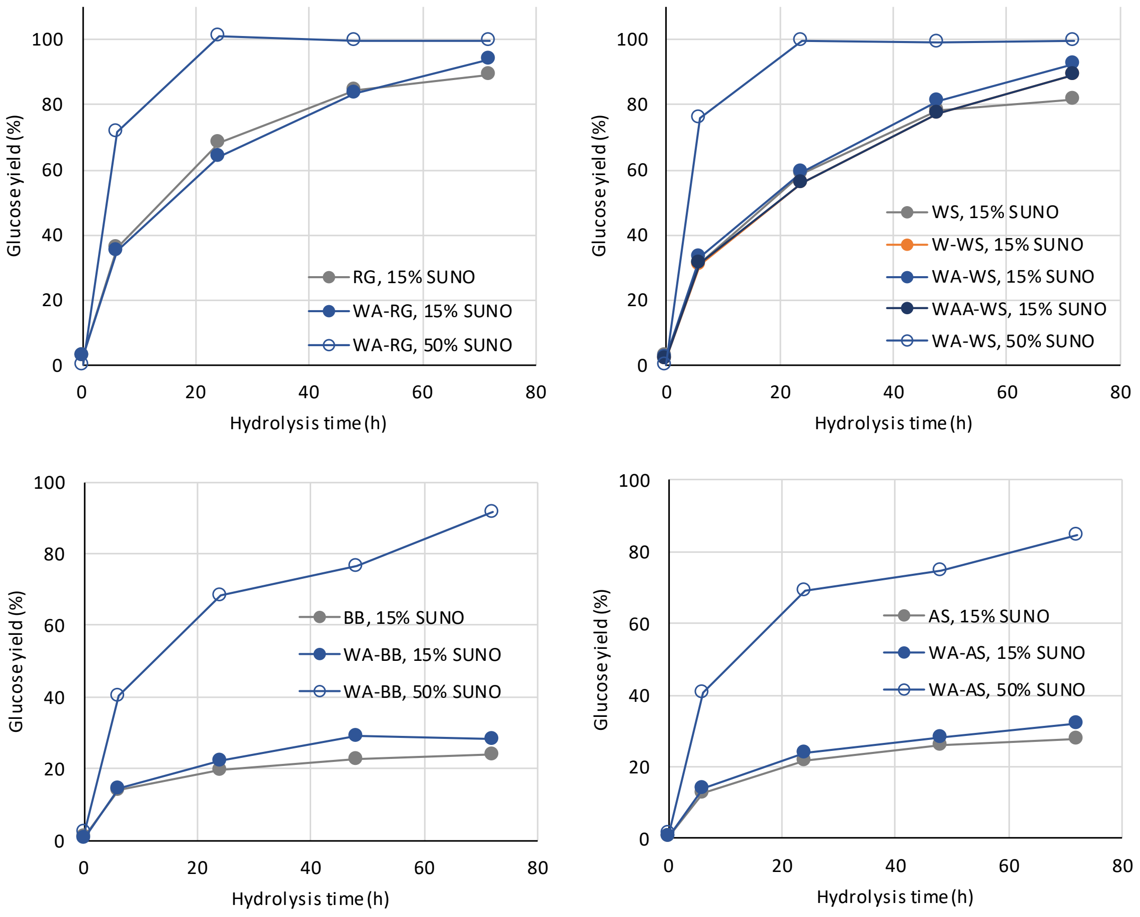

Figure S17 Glucose yield from the saccharification of pulps produced from untreated and pre-extracted feedstocks using 0.15 and 0.50 g MetZyme® SUNO^{TM} 036 /g pulp glucan.

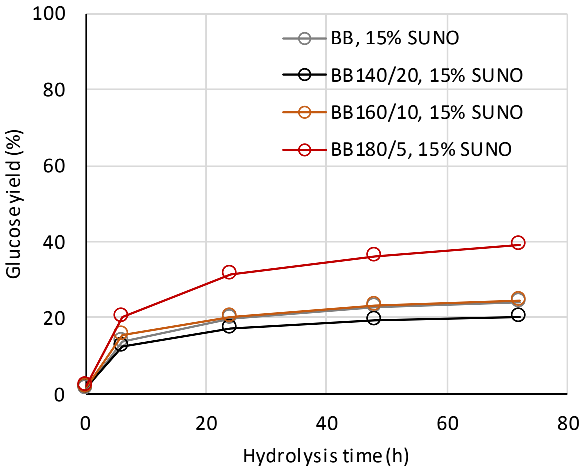

Figure S18 Glucose yields obtained from enzymatic saccharification of post-treated BB pulp (pulp treatment and composition are detailed in Table S15).

Hemicellulose hydrolysate

Experimental

For the detoxification of the hydrolysates, activated charcoal (AC) was added to 100 g of hydrolysate at weight ratios of 1.0 to 15.0 g AC / 100 g hydrolysate. Samples were agitated at room temperature for 30 min. Activated charcoal was isolated by centrifugation at 3488 *g* for 5 min.
AC from the 15.0 g AC / 100 g hydrolysate experiments were washed with 15 parts of demineralised water per part dry AC at room temperature. After centrifugation the isolated AC was washed with 4 parts of 100% acetone per part of dry AC followed by a wash with 11 parts of 60% acetone per part of dry AC to remove adsorbed components. Acetone was removed from the extraction liquid using rotary evaporation at 60 °C. For the samples where a precipitate was formed, the liquid was centrifuged and the precipitate collected and dried at 60 °C. Samples were taken from the hydrolysate, the detoxified hydrolysate, water wash and acetone wash for analysis of sugars, organic acids, furanics and phenolics.

Results

Hemicellulose mass balance

For the calculation of potential unknown components in the hemicellulose hydrolysate the amount of pulp, lignin and identified hydrolysate components were subtracted from the initial feedstock weight (normalised to 100 g). For this calculation the hydrolysate sugars and sugar derived furanics were recalculated to their polymeric origin. Formation of gypsum from pulp calcium and the sulfuric acid catalyst was not included and may cause a slight underestimation of the hydrolysate non-lignocellulose components. Sulfate incorporation into the pulp was quantified for W-WS and WAA-WS and was found to be 0.9 and 1.0 g / 100 g feedstock respectively. Because of the complex chemistry of biomass fractionation no other corrections were made and the data is best considered as an reasonable estimate. The rationale for including the unidentified hydrolysate components is to show that this mixture might be a significant factor that can affect chemo-catalytic and fermentative sugar conversion processes and subsequent downstream processing.

Potassium concentrations in the hydrolysate, calculated from the feedstock and pulp composition analysis, are 5.1, 3.0, 0.5 and 2.4 g/kg hydrolysate for RG, WS, BB and AS respectively, and concentrations of below 0.5 g/kg hydrolysate in the hydrolysates of pre-extracted feedstocks. Chloride and sulfate concentrations were analysed in the fractionation (wash) liquor. Relatively high chloride concentrations were observed only in the RG and WS liquors with 1.4 and 0.4 g/kg liquor, respectively. Only trace amounts of chloride was detected in the liquors of pre-extracted feedstocks. Fractionation (wash) liquors of RG, WS, BB and AS contained high concentrations of sulfate, i.e. 9.3, 6.3, 3.1 and 2.9 g/kg liquor, respectively. Feedstock pre-extraction reduced the acid dose requirements for fractionation and as a result lower sulfate concentrations were found in the liquors of pre-extracted feedstocks, i.e. 3.6, 3.1, 2.5 and 1.9 g/kg liquor for WA-RG, WA-WS, WA-BB and WA-AS, respectively.

Table S19 Indirect quantification of hemicellulose unidentified constituents.

Gram (dry weight basis)	Feedstock	Extracted feedstock	Pulp	Lignin	Quantified hydrolysate constituents	Unidentified hydrolysate constituents	Sulfate incorporated in pulp
RG	100		39.6	15.8	22.2	22.3	
WA-RG	100	71.8	34.5	8.6	19.8	8.9	
WS	100		49.5	14.5	24.8	11.1	
W-WS	100	88.8	48.7	12.1	21.5	6.6	0.9
WA-WS	100	89.3	48.7	10.8	22.9	6.9	
WAA-WS	100	88.6	46.8	11.4	22.5	7.9	1.0
A-WS	100	96.4	51.0	12.5	23.1	9.7	
BB	100		45.9	18.9	24.6	10.6	
WA-BB	100	90.2	44.4	14.0	22.7	9.0	
AS	100		42.7	21.0	27.3	8.9	
WA-AS	100	96.9	40.5	20.7	29.0	6.6	
WA-MIX	100	86.5	39.1	13.8	24.7	8.9	

Hemicellulose hydrolysate detoxification

Hydrolysates from each experiment were obtained after acetone and lignin removal from the organosolv (wash) liquors. The hydrolysates are combined with the lignin washing liquid after acetone removal and contain hemicellulose sugars, the solubilised cellulose sugars, organic acids, furanics, water-soluble lignin, phenolics, extractives, minerals and sulfuric acid. Especially furanics and phenolics have varying degrees of toxicity for microorganisms and can inhibit fermentation of sugars to fuels and chemical building blocks.[22-23] Therefore, these components have to be removed in a detoxification process step. A much studied and most basic approach for detoxification is to adsorb inhibitors on activated charcoal (AC) which have a high affinity for adsorbing phenolics, medium affinity for furanics and low affinity for organic acids. Hydrolysate detoxification process design at biorefinery scale will be a balanced design of streams recycled back to the fractionation process, hydrolysate concentration (thereby removing volatile organic acids and furanics) and detoxification. In this study a basic screening was conducted for hemicellulose hydrolysates using 1 – 15% activated charcoal (1 – 15 g AC / 100 g hydrolysate) to explore whether the matrix of organic components or inhibitors in the hydrolysate influences the adsorption capacity of the charcoal.

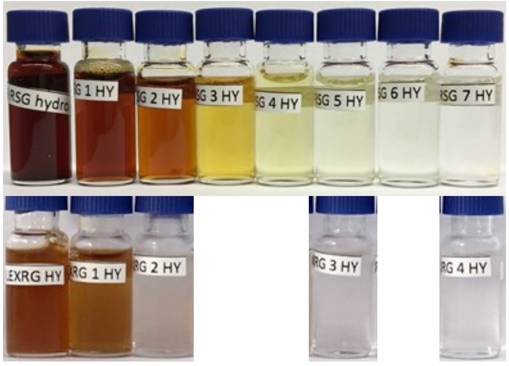

RG hydrolysate

WA-RG hydrolysate

Figure S19 Detoxified hydrolysates (0, 1, 2, 4, 6, 8, 10 and 15% activated charcoal loading).

The hydrolysate detoxification results for (WA-) RG, WS and BB using 0, 1, 4, 8 and 15% AC loading are shown in Figure S20. Acetic acid, furfural and syringaldehyde are shown as representative for each group of components (organic acids, furanics, phenolics) regarding adsorption affinity. Other components that were analysed were: formic acid, levulinic acid, HMF, 3,5 dihydroxybenzoic acid, 4-hydroxybenzoic acid, benzoic acid, phenol, guaiacol, syringol and vanillin.

Acetic acid is the most abundant inhibitor in all the samples and mainly originates from deacetylation of hemicellulose during fractionation. As expected, only minor differences were found in acetic acid concentration between hydrolysates from untreated and pre-extracted biomass (acetic acid is part of the lignocellulosic structure). The affinity of AC for organic acids is relatively low and detoxification results show only around 60% removal at the highest AC loading for all hydrolysates.
Due to the mild process conditions, lignin depolymerization to monomers is limited and low concentrations of phenolics were detected in the hydrolysate (Table S14). Combined with a high affinity for adsorbing phenolics, the detoxification results show efficient removal of syringaldehyde at low AC loadings.

Furfural has a relatively high concentration and medium affinity for adsorption to activated charcoal. The % w/w removal of furfural is mostly correlated with its concentration and no significant differences were found in AC adsorption capacity for untreated and pre-extracted hydrolysates. The primary effect of biomass pre-extraction on detoxification seems to be the reduced furanics concentration in the hydrolysate due to increased sugar stability during fractionation. Indirectly this does have a positive effect on detoxification as less AC is needed for hydrolysates from pre-extracted biomass, especially for RG and WS. Furfural concentrations in hydrolysates from BB and WA-BB were highly comparable to each other and showed identical detoxification results accordingly.

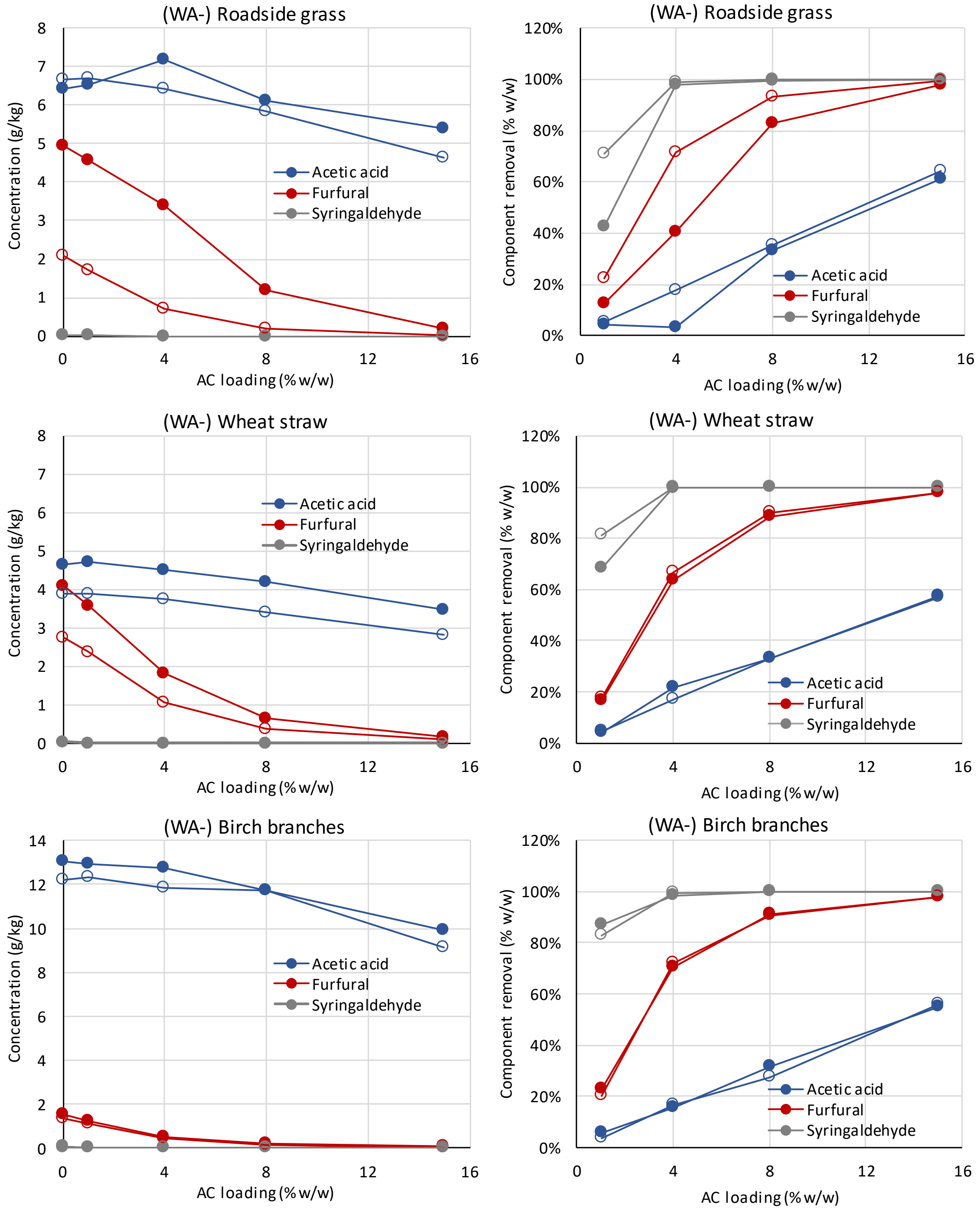

Figure S20 Detoxification of hydrolysates. Closed circles: RG, WS and BB, open circles: WA-RG, WA-WS and WA-BB.

Adsorbed components were recovered from the activated carbon, from the detoxification experiments with 15% AC, using aqueous acetone. Removal of solvent from the extraction liquid induced precipitation of organics which may contain water-soluble lignin (WSL). Most precipitate was collected from the hemicellulose hydrolysates of the fractionation of untreated feedstocks with 12, 15, 9 and 9 g for RG, WS, BB and AS, respectively (calculated to the total hydrolysate obtained per fractionation experiment). Only minor amounts were collected for the WA- hydrolysates (0 – 4 g), except for WA-AS (11 g). Quantification of WSL in the precipitates was not pursued due to suspected presence of non-lignocellulose components in the precipitate. The results are therefore not included in the feedstock lignin product distribution and mass balance.

Lignin

Experimental

For lignin fractionation 25 g of lignin (oven dry weight) was dissolved overnight in 250 mL of 60 % w/w aqueous acetone (corrected for lignin moisture content). Afterwards, the sample was centrifuged at 3488 *g* for 5 min. The liquid was decanted and demineralised water was added to obtain a 55% w/w aqueous acetone solution which was placed in an IKA shaking incubator (100 rpm) at room temperature for 30 min. The liquid was again centrifuged and decanted. This procedure was repeated to obtain lignin precipitate from (diluted) liquors containing 60 to 10% w/w aqueous acetone. All lignin pellets were dried in a conventional oven at 60 °C overnight and weighed. The 0 percent acetone sample was dried completely in a rotary evaporator at 60 °C and represents the fraction which was soluble in less than 10% w/w aqueous acetone.

Alkaline Size Exclusion Chromatography (SEC) was conducted in duplicate using a Thermo ICS-3000 system equipped with an thermostatic column oven operating at 40 °C and a Thermo VWD detector. 50 mg lignin was solubilised in 50 mL 0.5M NaOH. Injection volume was 100 µL. A commercially available column packed with PSS MCX, 5 µm particle size, 500A porosity, designed for polymers with a molecular size between 100-35.000 Da was used. The eluant was a solution of 0.5 M NaOH with a flow rate of 1 mL/min. An ultraviolet detector was used at 280 nm. The column was calibrated with poly(styrene sulfonate) sodium salts of different molecular sizes (94, 1830, 3610, 4900, 10.600, 13.200, 16.800, 29.100 and 32.900 Da).

High-field 2D NMR spectra were obtained on a Bruker Ultra 600 MHz spectrometer equipped with a Bruker 5 mm inverse triple resonance probe. ^{1}H-^{13}C Heteronuclear single quantum coherence spectroscopy (HSQC) spectra were recorded using the pulse sequence hsqcetgpsp.3 and the following parameters: A spectral window of 13 to -1 ppm was applied in F2 (1H) with 2048 collected datapoints and in F1 (13C), a spectral window of 160 to 0 ppm was applied with 256 scans, an interscan delay of 1 s and cnst2 set to 145 Hz. The spectra were processed with MestreNova software by first automatic baseline correction (polynomial fit 3rd order), followed by automatic phase correction. Apodisation was then performed by application of Gaussian (GB = 0.1), exponential functions (LB = 0.3 Hz) and a squared sine-bell function at 90° in F2, and a squared sine-bell function at 90° was applied in F1.

NMR samples were prepared by stirring approximately 200 mg of lignin in 750 µL DMSO-d6 overnight to ensure total dissolution. The chemicals shifts were referenced using the solvent signal as the shift reference (δC 39.6, δH 2.49 ppm). Semi-quantitative analysis of linkages was performed by using a section of the aromatic region as an internal standard, which was performed by setting the $S_{2,6}/2$ and G_2 integrals to 100 (i.e. the S region contains two C-H correlations so needs to be halved) for (WA-) BB and AS. For the herbaceous feedstocks (WA-) RG and WS the $S_{2,6}/2$, G_2 and $H_{2,6}/2$ integrals were used despite the overlap with extractives and residual proteins. The G_2 integral was further corrected for F_4 by subtracting the better isolated F_3 signal, the (furanic-type) unknown peak at δC/δH 112.6/6.6 was cut from the G_2 integral directly.
The alkyl area integral included DMSO-d6 and acetone peaks and were corrected accordingly. Peaks of interest were then referenced to the aforementioned aromatic region which then allowed for the expression of linkage integral values per 100 aromatic units (/100 Ar) as shown in Equation 1 where X denotes the unit of interest.

$$X = \frac{\int X}{\int \frac{S_{2,6}}{2} + \int G_2} \times 100$$

$$Equation\ 1.$$

Some the chosen integral regions of linkages correspond to multiple C-H correlations so correcting factors must be applied as shown in Table S20 below.

Table S20 Assignments and correction factors for integral regions of HSQC-NMR spectra

Notation	Chemical Shift (δ / ppm)	Assignment	Correction Factor
$-OCH_3$	55.6/3.73	C–H in methoxyls	$\int X$
$S_{2,6}$	104.3/6.6	C_2–H_2 and C_6–H_6 in syringyl units	$\int X/2$
$S'_{2,6}$	106.5/7.3	C_2–H_2 and C_6–H_6 in syringyl units with α oxidization	$\int X/2$
G_2	110.8/6.9	C_2–H_2 in guaiacyl units	$\int X$
G'_2	111.6/7.5	C_2–H_2 in guaiacyl units with α oxidization	$\int X$
G_5	115.3/6.8	C_5–H_5 in guaiacyl units	$\int X$
G_6	119.0/6.9	C_6–H_6 in guaiacyl units	$\int X$
G'_6	123.5/7.6	C_6–H_6 in guaiacyl units with α oxidization	$\int X$
$H_{2,6}$	128.2/7.2	$C_{2,6}$–$H_{2,6}$ in *p*-hydroxyphenyl units	$\int X/2$
$T_{2,6}$	104.2/7.3	C_2–H_2 and C_6-H_6 in tricin units	$\int X/2$
T_3	104.5/7.1	C_3–H_3 in tricin units	$\int X$
T_6	98.9/6.3	C_6–H_6 in tricin units	$\int X$
T_8	94.2/6.6	C_8–H_8 in tricin units	$\int X$
Fa_2	111.2/7.3	C_2–H_2 in ferulates	$\int X$
Fa_6	122.9/7.1	C_6–H_6 in ferulates	$\int X$
$Pca_{2,6}$	130.1/7.5	C_2–H_2 and C_6–H_6 in *p*-coumarate	$\int X/2$
A_α	71.9/4.9	C_α–H_α in β-O-4′ substructures	$\int X$
A_β	84.8/4.3 (G) and 86.0/4.2 (S)	C_β–H_β in β-O-4′ substructures	$\int X$
A_γ	59.8/3.4	C_γ–H_γ in γ– hydroxylated β-O-4′ substructures	$\int X$
A'_β	83.1/5.3	C_β–H_β in α-oxidized β-O-4′ substructures	$\int X$
B_α	87.3/5.5	C_α–H_α in phenylcoumaran substructures	$\int X$
B_β	53.1/3.4	C_β–H_β in phenylcoumaran substructures	$\int X$
B_γ	62.6/3.7	C_γ–H_γ in phenylcoumaran substructures	$\int X$
C_α	85.2/4.7	C_α–H_α in β–β′ resinol substructures	$\int X/2$
C_β	53.6/3.1	C_β–H_β in β–β′ resinol substructures	$\int X/2$
C_γ	71.2/4.2 and 71.3/3.8	C_γ–H_γ in β–β′ resinol substructures	$\int X/2$
C'_α	86.9/4.4	C_α–H_α in β–β′ epiresinol substructures	$\int X$
$C'_{\alpha'}$	81.4/4.8	$C_{\alpha'}$–$H_{\alpha'}$ in β–β′ epiresinol substructures	$\int X$
C'_β	70.3/4.1 and 70.3/3.7	C_β–H_β in β–β′ epiresinol substructures	$\int X$
$C'_{\beta'}$	68.9/3.8 and 68.9/3.1	$C_{\beta'}$–$H_{\beta'}$ in β–β′ epiresinol substructures	$\int X$
C'_γ	53.9/2.8	C_γ–H_γ in β–β′ epiresinol substructures	$\int X$
I_α	128.2/6.4	C_α–H_α in cinnamyl alcohol end-groups	$\int X$
I_β	128.3/6.2	C_β–H_β in cinnamyl alcohol end-groups	$\int X$
I_γ	61.0/4.1	C_γ–H_γ in cinnamyl alcohol end-groups	$\int X$
J_α	153.4/7.61	C_α–H_α in cinnamaldehyde end-groups	$\int X$
J_β	126.0/6.8	C_β–H_β in cinnamaldehyde end-groups	$\int X$

Z-EE_{α}	109.3/5.6	C_{α}–H_{α} in *Z*-enol ether	$\int X$
E-EE_{α}	112.0/6.1	C_{α}–H_{α} in *E*-enol ether	$\int X$
$SB1_{\alpha}$	125.6/7.0	C_{α}–H_{α} in *trans*-stilbene substructures (β-1)	$\int X/2$
$SB2_{\beta}$	119.9/7.2	C_{β}–H_{β} in *trans*-stilbene substructures (β-5)	$\int X$
Hk_{γ}	67.2/4.2	C_{γ}–H_{γ} in Hibbert ketone structures	$\int X/2$
F_3	122.8/7.5	C_3– H_3 in furfural	$\int X$
F_4	112.6/6.8	C_4– H_4 in furfural	$\int X$
F_5	148.9/8.1	C_5– H_5 in furfural	$\int X$
HMF_3	124.3/7.5	C_3– H_3 in 5-hydroxymethylfurfural	$\int X$
HMF_4	109.6/6.6	C_4– H_4 in 5-hydroxymethylfurfural	$\int X$
HMF_6	55.8/4.5	C_6– H_6 in 5-hydroxymethylfurfural	$\int X/2$

For ^{31}P NMR analysis, accurately weighted samples (20 mg) were dissolved in 150 µL of N,N-dimethylformamide. After dissolution, 100 µL pyridine, 100 µL internal standard solution of 0.05 M endo-N-Hydroxy-5-norbornene-2,3-dicarboximide in pyridine/$CDCl_3$ (1.6/1, v/v) and 50 µL $Cr(acac)_3$ solution (11.4 mg/1 mL) in pyridine/$CDCl_3$ (1.6/1, v/v) were added. Then, 100 µL 2-chloro-4,4,5,5-tetramethyl-1,3,2-dioxaphopholane was added drop-wise, followed by 300 µL of $CDCl_3$. The ^{31}P NMR measurements were performed immediately after sample preparation at room temperature with a Bruker Avance 500 MHz NMR spectrometer using 90° pulse and 5s pulse delay for 512 scans. Note that no duplicate analyses were conducted on the selected lignins.

Pyrolysis-GC-MS was performed on a Gerstel PYRO automated pyrolyzer. The sample (0.5-1 mg) was weighed with an accuracy of 0.01 mg in a quartz sample holder, an open tube type with quartzwool. The sample tube is attached to a sample adaptor which is introduced into the pyrolysis module by an autosampler (MultiPurpose Sampler MPS). The sample pyrolysis was performed at 400 °C, heating rate 100 °C/s, hold time = 30 sec. Gas Chromatography was performed using an Agilent GC 7890A MSD 5975C system equipped with a capillary column (Zebron ZB-WAXplus, 30 m x 0.25 mm x 0.25 µm). The GC was equipped with a Gerstel Cooled Injection System (CIS) PTV-type inlet used as a cryo-focusing trap for the pyrolysis products. The injection temperature was 250 °C, the split ratio was 40. Helium was used (99.9999 %) as carrier gas in a constant flow mode at 1 mL/min. The temperature program of the GC oven was: 50 °C for 5 min isothermal and heating up with 10 °C/min to 250 °C.

Results

The sugar content of the precipitated lignin was determined by biochemical composition analysis. However, the content of organic acids, furanics and phenolics in lignin is more difficult to determine. Therefore, a mass balance was composed from the liquors obtained from (pre-extracted) RG, WS, BB and AS before and after lignin precipitation. The components that are missing from the precipitation mass balance can give some indication of lignin purity. The mass balance is composed of sugar, organic acid, furanic and phenolic components quantification in the:

- Combined liquor: organosolv liquor + 50% acetone pulp wash liquid.
- Combined hydrolysate + lignin wash liquid after acetone and lignin removal.
- Condensate: acetone vapours are cooled, condensed and collected separately.

Figure S21 shows how much of each component is missing after lignin precipitation. The data is presented as an average of all experiments for each compound. The weight of the missing components was calculated as % w/w of the precipitated lignin (Figure S21 right). Analysis of the acetone condensate showed that only a minor amount of the acetic acid and on average 30% of the furfural was evaporated. Missing sugars showed relatively high variation between samples which might be due to the summative deviations in the sugar analysis. Biochemical composition analysis of the lignin (Table S21 and Table S22) shows that the lignin sugar content is low (1.1 – 2.8%). Significant amounts of furanics and phenolics were missing after lignin precipitation. However, due to the low concentration of HMF, vanillin and syringaldehyde only a minor contribution to lignin impurity is calculated. Due to its higher concentration in the liquor, the 10% furfural missing from the mass balance would contribute to furfural content of 1.2% in the isolated lignin. The data indicate that furfural inclusion into the precipitated lignin far exceeds that of the sugars which are present in significantly higher concentrations, possibly due to favourable interaction with lignin structure and thus promoting inclusion in the lignin precipitate. Overall, the precipitation mass balance and lignin biochemical composition analysis indicate that the contribution of sugars, organic acids, furanics, phenolics and ash to lignin impurity is limited.

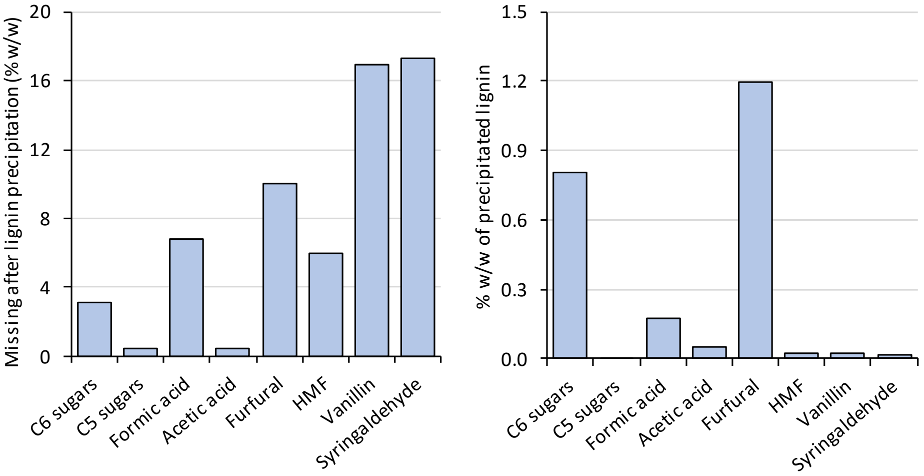

Figure S21 Lignin precipitation mass balance closure.

Biochemical composition analysis of lignin precipitated from the fractionation (wash) liquor and pulp water wash are shown in Table S21 and Table S22. Due to the applied hydrolysis steps in the composition analysis a differentiation between free monomeric sugars and lignin-carbohydrate complexes cannot be made. The lignin content is determined by the ash free weight of the solids after hydrolysis (acid-insoluble lignin, AIL) plus the quantified acid-soluble lignin in the hydrolysate (ASL). The lignin content is not included in the fractionation mass balances as this method does not differentiate between extractives and lignin.

Table S21 Biochemical composition of precipitated lignin.

% w/w	Polymeric C5 sugars		Polymeric C6 sugars					
(dry weight basis)	Xylan	Arabinan	Glucan	Galactan	Mannan	Rhamnan	Lignin	Ash
RG	0.4	0.3	0.4	[a]			92.0	0.3
WA-RG	0.8	1.2	0.2				91.2	0.5
WS	0.6	0.6	0.2				94.2	0.5
W-WS	0.9	0.9	0.2				93.6	0.5
WA-WS	1.0	0.9	0.2				93.7	0.3
WAA-WS	1.1	1.1	0.2				92.9	0.4
A-WS	0.7	0.7	0.1				93.9	0.3
BB	1.7	0.1	0.4	0.2			92.6	0.1
WA-BB	1.2		0.2	0.1			93.1	0.1
AS	2.6	0.1		0.1			90.8	0.1
WA-AS	1.8	0.3	0.4				92.7	0.0
WA-MIX	0.9	0.4					94.7	0.1

[a] Empty cell: below detection limit.

Table S22 Biochemical composition of pulp waterwash lignin.

% w/w (dry weight basis)	Polymeric C5 sugars		Polymeric C6 sugars					
	Xylan	Arabinan	Glucan	Galactan	Mannan	Rhamnan	Lignin	Ash
RG	0.7	0.4	0.6	[a]			87.4	2.2
WA-RG	1.5	1.1	0.7	0.1			88.1	0.9
WS	1.3	0.6	0.4				91.1	1.5
W-WS	1.6	0.9	1.6				90.1	1.1
WA-WS	1.3	0.9	0.3				91.9	0.0
WAA-WS	1.3	0.9	0.3				91.8	0.9
A-WS	0.9	0.7	0.3				93.3	1.0
BB	0.8		0.2				91.2	2.8
WA-BB	1.5	0.1	0.2	0.1			88.6	2.2
AS	1.3						90.7	0.7
WA-AS	1.3	0.4	0.6				90.3	0.5
WA-MIX	ND [b]							

[a] Empty cell: below detection limit. [b] ND, not determined

Py-GC/MS analysis generated 75-139 different components which are categorised in Figure S22. Lignin obtained from fractionation of pre-extracted biomass show increased abundance of phenolics in all major categories (guaiacols, syringols, p-hydroxyphenols, catechols and other aromatics) except for some variation in low abundance groups in WA-BB and WA-AS. The relative peak area of volatile fatty acids shows a large decline for the lignin obtained from pre-extracted feedstocks. This indicates significantly increased lignin purity as a result of biomass pre-extraction. Remarkably, the category "others" does not show a significant decrease for the WA-lignins, except for WA-RG.

Figure S23 shows the correlation between volatile fatty acid abundance as determined by Py-GC/MS and the lignin yield after fractionation. The lignin yield is defined as the percentage of solubilised lignin in the fractionation liquor that is obtained as solid lignin after precipitation. Lignin yields exceeding 100% indicate the presence of non-lignin components such as extractives. WA-lignins show a lower lignin yield which correlates well with the reduced response from these components from the Py-GC/MS analysis. However, a large difference in lignin yield between W-WS and WS is not explained by fatty acid content only. For all feedstocks a combined effect of water-soluble and solvent-soluble extractives plays a role during fractionation and will affect lignin yield and purity.

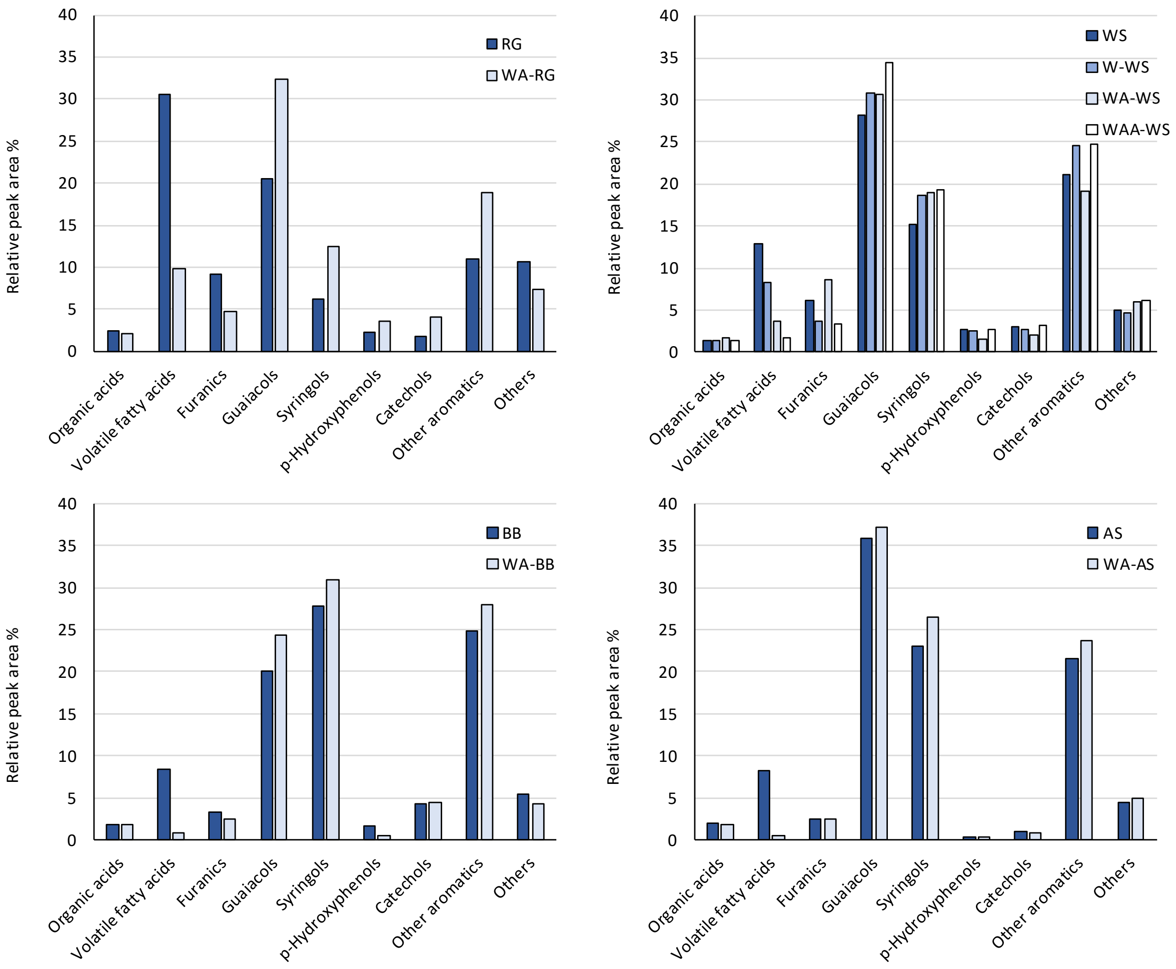

Figure S22 Lignin Py-GC/MS response per component group.

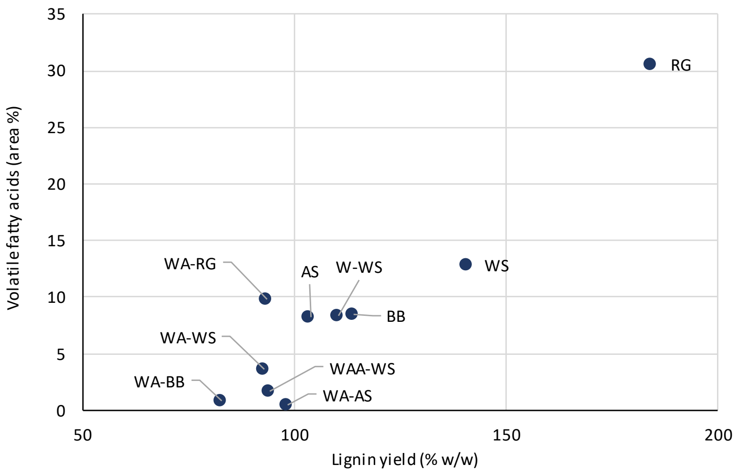

Figure S23 Correlation lignin volatile fatty acid signal (% of total Py-GC/MS analysis peak area) and lignin precipitation yield.

Table S23 Lignin Py-GC/MS response for a selection of components.

Relative peak area		RG	WA-RG	WS	W-WS	WA-WS	WAA-WS	BB	WA-BB	AS	WA-AS
Organic acids											
Formic acid		0.1	0.1	0.1	0.1	0.2	0.1	0.1	0.1	[a]	0.1
Acetic acid		2.2	1.9	1.1	1.2	1.4	1.2	1.6	1.6	2.0	1.7
Propanoic acid		0.1	0.1	0.1	0.0	0.1	0.1	0.0	0.1		
Fatty acids											
Caprylic acid	Octanoic acid	0.1	0.1	0.1				0.1	0.1		
Myristic acid	Tetradecanoic acid	0.5	0.2	2.1	1.2	0.7	0.3	0.2			
Palmitic acid	n-Hexadecanoic acid	12.6	4.2	5.1	4.4	3.0	1.2	3.3	0.7	1.5	0.5
Stearic acid	Octadecanoic acid			0.3	0.3			0.4			
Linoleic acid	9,12-Octadecadienoic acid	6.1	1.9	3.1	1.3			3.4		1.1	
Linolenic acid	9,12,15-Octadecatrienoic acid	10.7	3.3	1.0							
Oleic acid	Octadecenoic acid			1.1	0.8			0.8		5.6	
Lauric acid	Dodecanoic acid	0.2		0.2	0.1		0.1				
Furanics											
Furfural	2-Furancarboxaldehyde	6.6	4.4	5.7	3.4	8.1	3.0	2.9	2.2	2.3	2.4
HMF	2-Furancarboxaldehyde, 5-(hydroxymethyl)-	2.2	0.2	0.3	0.2	0.4	0.2	0.3			
Guaiacols											
Guaiacol	Phenol, 2-methoxy-	2.9	5.7	4.3	4.1	3.6	4.9	2.4	3.1	4.9	4.2
4-Ethyl guaiacol	Phenol, 4-ethyl-2-methoxy-	2.2	3.0	2.0	1.8	0.9	1.9	0.8	1.1	1.6	1.4
4-propyl guaiacol	Phenol, 2-methoxy-4-propyl-	0.1	0.2	0.2	0.2	0.1	1.4	0.1	0.2	3.6	4.1
4-Vinyl guaiacol[b]	2-Methoxy-4-vinylphenol	10.8	14.2	12.0	12.9	11.2	13.5	4.1	3.6	5.0	4.9
Guaiacylacetone	2-Propanone, 1-(4-hydroxy-3-methoxyphenyl)-	0.6	1.0	1.1	1.2	1.4	1.3	1.4	1.7	2.0	2.1
Creosol	Phenol, 2-methoxy-4-methyl-	1.7	4.5	4.3	5.0	4.2	5.5	3.5	4.6	6.4	7.4
Eugenol	4-Allyl-2-methoxyphenol	0.2	0.3	0.3	0.4		0.4	0.3	0.4	0.6	0.7
Isoeugenol[c]	Phenol, 2-methoxy-4-(1-propenyl)-, (E)-	1.0	1.7	1.6	1.9	2.1	1.9	4.2	5.3	3.9	3.3
γ-Hydroxy-isoeugenol	4-((1E)-3-Hydroxy-1-propenyl)-2-methoxyphenol			0.4	0.7	3.8	0.8	0.9	1.0	1.6	2.7
Vanillin	4-Hydroxy-3-methoxybenzaldehyde	0.6	1.1	1.2	1.6	2.4	1.8	1.1	1.6	3.5	3.6
Acetovanillone	Ethanone, 1-(4-hydroxy-3-methoxyphenyl)-	0.3	0.6	0.7	0.8	0.9	0.9	0.7	0.9	1.4	1.6
Homovanillic acid	Benzeneacetic acid, 4-hydroxy-3-methoxy-	0.1	0.1	0.1	0.1		0.1	0.5	0.7	1.2	1.2
Syringols											
Syringol	Phenol, 2,6-dimethoxy-	2.2	4.7	5.2	5.0	4.6	6.1	8.3	10.4	8.1	6.8
Syringaldehyde	Benzaldehyde, 4-hydroxy-3,5-dimethoxy-	0.5	0.9	1.3	1.7	2.6	2.1	4.3	6.0	4.3	4.8
Sinapaldehyde	3,5-Dimethoxy-4-hydroxycinnamaldehyde				1.8		1.0	2.1	2.4	2.5	2.4
acetosyringone	3',5'-Dimethoxyacetophenone	1.7	3.2	3.9	4.4	5.3	4.4	9.6	7.5	4.9	4.9
Methoxy eugenol	Phenol, 2,6-dimethoxy-4-(2-propenyl)-	1.3	2.2	2.7	3.5	4.2	3.4	1.4	1.9	1.2	5.4
Acetovanillone	Ethanone, 1-(4-hydroxy-3,5-dimethoxyphenyl)-	0.6	1.4	2.0	2.3	2.3	2.4	2.0	2.8	1.9	2.3
p-Hydroxyphenols											
Phenol	Phenol	0.9	1.4	0.8	0.8	0.7	0.9	0.2	0.1	0.2	
P-cresol	Phenol, 4-methyl-	0.6	1.2	0.9	0.8	0.6	1.0	1.1	0.4	0.2	0.3
	Phenol, 4-ethyl-	0.9	1.1	1.1	0.9	0.3	0.8	0.3			
Catechols											
Catechol	1,2-Benzenediol	0.7	1.5	0.8	0.7	0.5	0.8	1.4	1.0	0.7	0.6
	1,2-Benzenediol, 4-methyl-	0.1	0.4	0.2	0.2	0.1	0.3	0.9	0.5	0.3	0.3
	1,2-Benzenediol, 3-methoxy-	0.8	2.3	1.9	1.7	1.4	2.1	2.0	2.9		
Other aromatics											
	Benzofuran, 2,3-dihydro-	7.4	10.3	9.7	11.0	8.3	11.7			0.1	
	1,2,4-Trimethoxybenzene	1.1	2.9	4.1	4.7	4.1	5.2	10.1	12.6	8.4	9.4
	Benzene, 1,2,3-trimethoxy-5-methyl-	0.7	1.2	1.0		0.1	1.0		2.5	2.3	2.6
Acetone (self-) condensation products											
Furfural acetone	3-Buten-2-one, 4-(2-furanyl)-	5.3	2.9	4.0	2.6	6.3	2.9	1.9	2.0	0.6	0.8
Diacetone alcohol	2-Pentanone, 4-hydroxy-4-methyl-	0.1	0.3	0.3	0.2	0.7	0.2	0.4	0.3	0.3	0.4
Mesityloxide	3-Penten-2-one, 4-methyl-	0.2	1.3	0.3	0.2	0.8		0.5	0.2	0.3	0.5
Sum nitrogen containing compounds[d]		2.2	2.4	1.1	0.7	1.8	0.8	0.6	0.6	0.5	0.5

[a] Not detected. [b] Pyrolysis product from guaiacols and ferulates.[12] [c] Cis- + trans-isoeugenol. [d] mostly pyridines, pyrroles and pyrodinoles.

Lignin fractionation was conducted to assess lignin solubility in aqueous acetone mixtures and obtain insights into the distribution of non-lignin components over the lignin fractions. Fractionation of WS and WAA-WS lignin show slight differences in their fractionation pattern (Figure S24). However, it remains unclear to what extent the shift originates from changes in lignin characteristics or from the presence of extractives such as fatty acids. A selection of WS and WAA-WS lignin fractions were analysed by size-exclusion chromatography and the weight average molecular weight (Mw) of each fraction is shown in the data labels of Figure S24. WS lignin molar mass is 2700 g/mol with a polydispersity (PD) of 4.1. WS lignin fractionation produces fractions with an Mw of 4650 to 1340 g/mol. Polydispersity in these fractions follow a similar decreasing trend with 4.4, 3.0, 2.3, and 1.9 for the 45%, 35%, 25% and 10% acetone fractions respectively. WAA-WS lignin fractions show slightly lower Mw (except for the 45% acetone fraction) and a similar PD as compared to WS lignin. The percentage of lignin that did not dissolve in 60% w/w acetone was significantly higher for WS lignin than for WAA-WS lignin.

Figure S25 shows the distribution of fatty acids over WS and WAA-WS lignin fractions (as shown in Figure S24). Myristic acid, palmitic acid and stearic acid signal abundance is highest for the 45% acetone lignin fraction and declines in the lignin fractions obtained at a lower acetone concentration. Organic acids, mostly acetic acid, shows a relatively similar abundance for all lignin samples which indicates that the acetic acid originates from the lignin itself (or by acetylation) instead of inclusion in the lignin sample during precipitation. Pyrolysis phenolic product abundance as identified by Py-GC/MS are relatively similar for all analysed (WAA-) WS lignin fractions. Phenolics with a high abundance such as guaiacol, 4-vinyl guaiacol, syringol and 2,3-dihydro-benzofuran show an increase in each consecutive lignin fraction, most likely caused by decreased presence of fatty acids i.e. increased lignin purity.

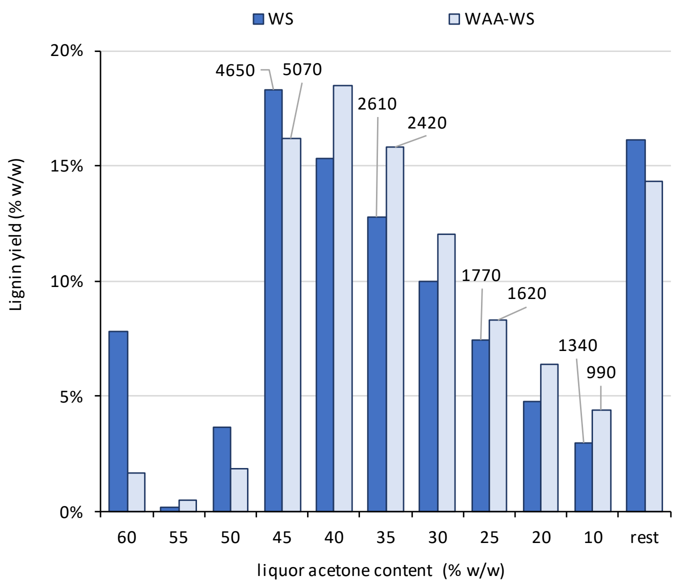

Figure S24 Fractionation results of WS and WAA-WS lignin. Labels show the weight average molecular weight of the fractions as determined by alkaline HPSEC.

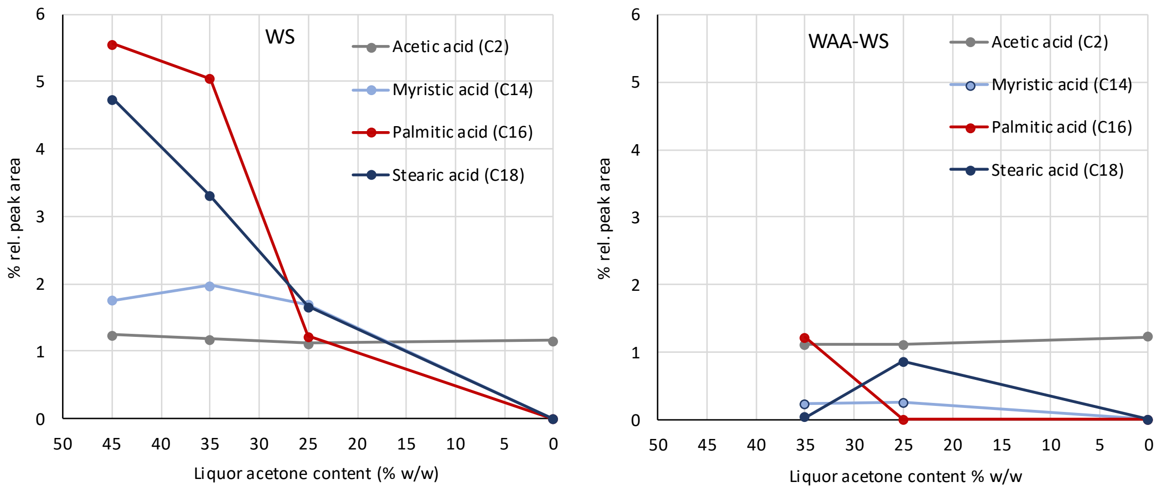

Figure S25 Py-GC/MS analysis results of WS and WAA-WS lignin fractions as reported in Figure S24

Table S24 shows the main characteristics of lignin obtained from untreated and pre-extracted feedstocks. Note that lignin self-assembly and aggregation may play a role during alkaline SEC and that the lignin molar masses may be overestimated.[24] Analysis of the aromatic region of 2D HSQC-NMR spectra (Figure S26) was complicated by contributions from furanics, for which distinct peaks were found near and in the G_2 cross-peak. Where HMF was mostly detected in RG, furfural was observed in all lignin samples. Furfural peak intensity correlated well with the calculated lignin furfural content from the lignin precipitation mass balance as well as Py-GC/MS results (Table S23). An improved lignin washing procedure may further reduce lignin furanics content. F_4 is located inside the G_2 cross peak and therefore the F_4 integral contains both F_4 and G_2 which may lead to overestimation of G-unit abundance. Therefore, F_3 was used for correction of the G_2 integral. The G_2 integral was further corrected for an unidentified peak which appeared at δC/δH 112.6/6.6 and showed a close correlation with the intensity of furfural peaks. Further study is required to assess whether this peak originates from lignin-furfural or acetone-furfural condensates.

In general, herbaceous lignins are more complex than hardwood, having e.g. hydroxycinnamic acids (p-coumaric and ferulic acids) and tricin in the native lignin structure.[25] Tricin was partially lost upon fractionation, as previously found by Constant et al.[26] Table S24 shows the abundance of H units for all lignins and as birch lignin typically contains solely G and S units, the H units abundance is likely to originate from protein-derived phenylalanine.[27] BB lignin is relatively rich in syringyl units and S/G ratio found is similar as reported for birch wood lignin, despite possible higher abundance of condensed G units in the bark.[21, 28-31] AS lignin showed a more equal distribution of S- and G-units, in accordance with almond shell Py-GC/MS results reported by Queiros et al.[7] Hibbert ketones abundance was found to be 3 to 4 per 100 Ar with similar values for the pre-extracted feedstock lignins. Small amounts of enol ethers and lignin condensation structures such as stilbenes were detected in most cases and, surprisingly, no peaks were observed for I_α, I_β and I_γ (Table S25). Cinnamaldehyde end groups (J_α) ranged from 0.3 to 2.2 per 100 Ar.

Table S24 Isolated lignin characteristics

	RG	WA-RG	WS CEL	WS[a]	WA-WS	BB	WA-BB	AS	WA-AS
	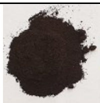	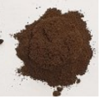		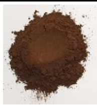	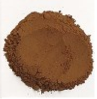	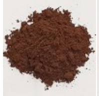	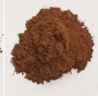	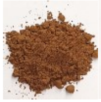	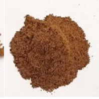
Molar mass[b]									
Mn	910	920	[h]	690	780	1120	1040	980	1000
Mw	2840	2640		2700	3010	3830	3360	3450	3460
Mw/Mn	3.1	2.9		4.1	4.1	3.4	3.2	3.5	3.5
Quantification of identified 2D HSQC NMR structures									
Guaiacyl (G_2)[c]	60	61	60	56	55	31	32	51	51
Syringyl ($S_{2/6}$)[c]	28	30	36	40	41	66	67	48	49
p-Hydroxyphenyl ($H_{2/6}$)[c, d]	12	9	5	3	4	3	1	1	1
S/G ratio	0.5	0.5	0.6	0.7	0.7	2.1	2.1	0.9	1.0
Tricin (T_8)[c]	8	5	7	3	3	0	0	0	0
Ferulate (Fa_6)[c]	10	8	3	5	5	0	0	0	0
p-Coumarate ($Pca_{2/6}$)[c]	13	11	6	10	9	0	0	0	0
β-Aryl ether (A_α)[e]	14	15	41	12	13	14	14	17	15
Oxidised β-Aryl ether (A'_β)[e]	2.4	2.9		2.2	2.4	1.8	1.8	1.4	1.5
Resinol (C_α)[e]	1	1	2	1	1	4	4	3	3
Epiresinol (C'_α)[e]	1.2	1.5		1.5	1.5	2.9	3.0	2.7	2.6
Phenylcoumaran (B_α)[e]	3	3	6	3	3	2	2	4	4
Hibbert's ketones (Hk_γ)[e]	3	3	0	3	3	4	3	3	3
Fatty acids[f]	45	12	1	9	0	6	0	4	0
Alkyl region[f]	1284	498	227	517	238	585	181	154	82
Hydroxyl groups content[g]									
Aliph. OH	1.97	2.10		1.88	2.24	2.37	2.50	3.38	3.03
5-subst. Ph-OH	0.85	0.91		1.18	1.30	1.77	1.99	1.47	1.65
G Ph-OH	0.89	0.89		0.92	0.95	0.83	0.73	0.93	0.97
H Ph-OH	0.46	0.49		0.42	0.46	0.42	0.15	0.12	0.14
Tricin	0.12	0.12		0.09	0.09	0.02	0.01	0.01	0.01
Total Ph-OH	2.21	2.29		2.52	2.72	3.01	2.87	2.52	2.76
COOH	0.78	0.53		0.53	0.43	0.32	0.23	0.22	0.18
Total OH	4.18	4.39		4.40	4.96	5.38	5.37	5.90	5.79

[a] Mw of W-WS, WAA-WS and A-WS are 2970, 2650 and 3030 g/mol respectively. [b] Determined by Size Exclusion Chromatography, values in g/mol. [c] Aromatic units RG and WS lignin expressed as percent of S + G + H, aromatic units BB and AS lignin as percent of S + G. [d] Abundance of *p*-hydroxyphenyl units may (partly) originate from overlap with the protein-derived phenylalanine cross peak. [e] RG and WS structures/linkages expressed per 100 aromatic units (S + G + H), BB and AS structures/linkages expressed per 100 aromatic units (S + G). [f] Fatty acid signal in aromatic region δ 127.5-129.7/5.3 and whole alkyl region (corrected for DMSO and acetone peaks) expressed per 100 aromatic units (S + G + H). [g] Determined by ^{31}P NMR, mmol OH groups/g lignin. [h] Empty cell: not determined.

Table S25 2D HSQC-NMR semi-quantitative analysis of lignin minor structures.

Per 100 aromatic units		RG	WA-RG	WS CEL	WS	WA-WS	BB	WA-BB	AS	WA-AS
Cinnamaldehyde end-groups	J_α	0.0	0.0	1.2	0.3	0.3	0.9	1.0	2.2	1.9
	J_β	0.6	0.6	0.9	1.0	0.7	1.4	0.9	2.1	1.6
Cinnamyl alcohol end-groups	I_α	0.0	0.0	0.9	0.0	0.0	0.0	0.0	0.0	0.0
	I_β	0.0	0.0	0.8	0.0	0.0	0.0	0.0	0.0	0.0
Enol ether	*E*-EE_α	0.0	0.0	0.0	0.1	0.2	0.1	0.2	0.2	0.3
	Z-EE_α	0.0	0.0	0.0	0.0	0.2	0.0	0.1	0.3	0.1
Stilbene	$SB5_\beta$	0.5	0.6	0.2	0.4	0.4	0.0	0.1	0.2	0.2
	$SB1_\alpha$	0.7	0.4	0.1	0.4	0.3	0.2	0.2	0.2	0.2
Secoisolariciresinol	SR_β	0.3	0.0	0.0	0.1	0.0	0.1	0.0	0.2	0.3
Furfural	F_3	7.7	3.2	0.5	6.2	3.2	1.6	1.2	1.3	1.3
	F_4	11.3	6.6	0.0	10.3	6.4	3.7	3.3	4.1	4.6
	F_5	6.4	3.0	0.0	5.8	2.4	1.1	0.9	0.5	0.6
5-hydroxymethylfurfural	HMF_3	3.8	0.6	0.0	1.1	0.3	1.0	0.3	0.2	0.7
	HMF_4	4.9	0.7	0.0	1.2	0.8	1.1	0.8	1.0	0.8
	HMF_6	4.5	0.6	0.0	0.4	0.5	0.4	0.2	0.2	0.3
Unknown structure in G_2 cross peak[a]		7.0	3.4	0.0	5.0	3.5	2.1	1.8	2.1	2.1

[a] δC/δH 112.6/6.6

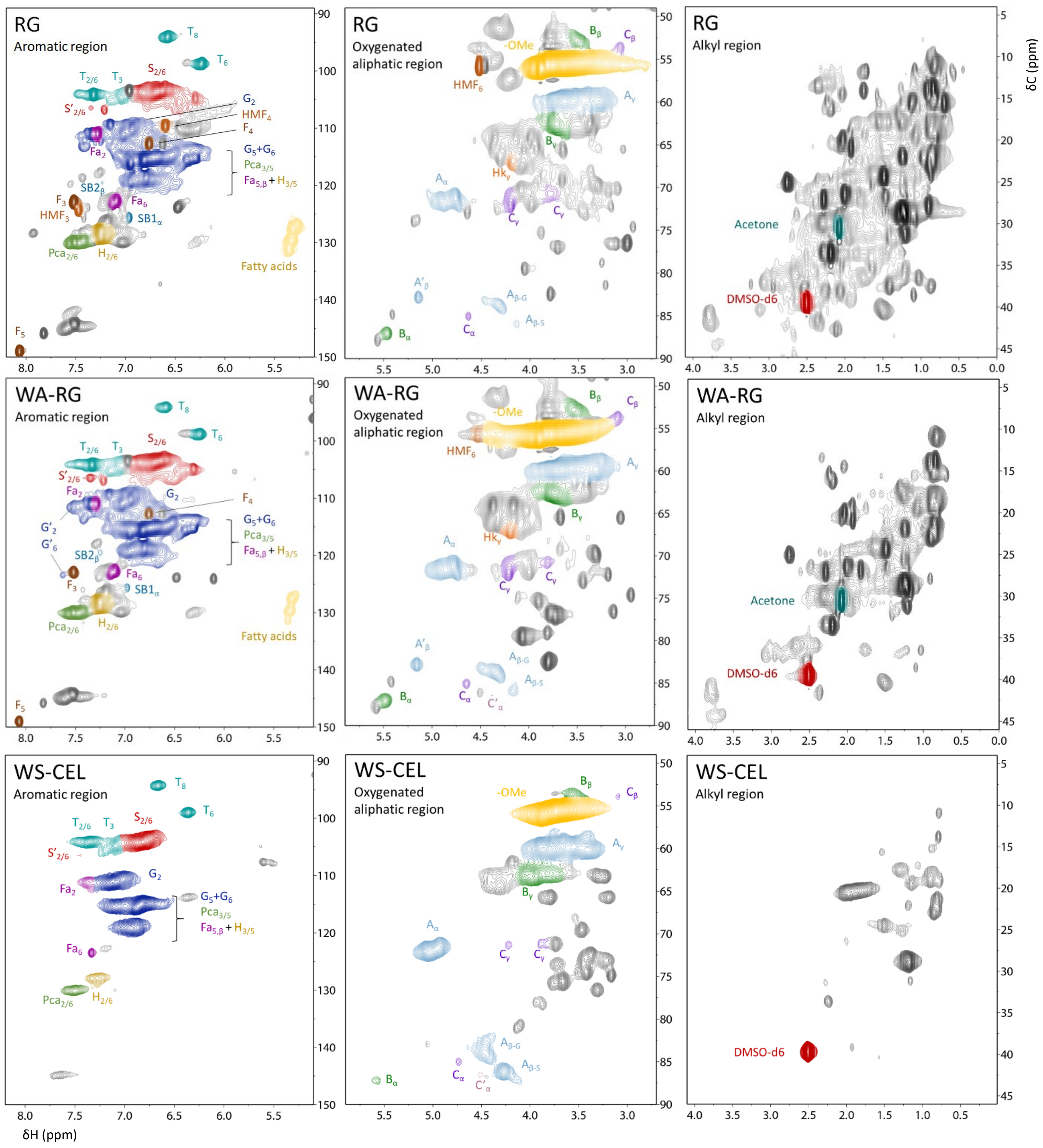
RG
Aromatic region
Oxygenated aliphatic region
Alkyl region
WA-RG
WS-CEL
Fatty acids
Acetone
DMSO-d6
-OMe
δH (ppm)
δC (ppm)

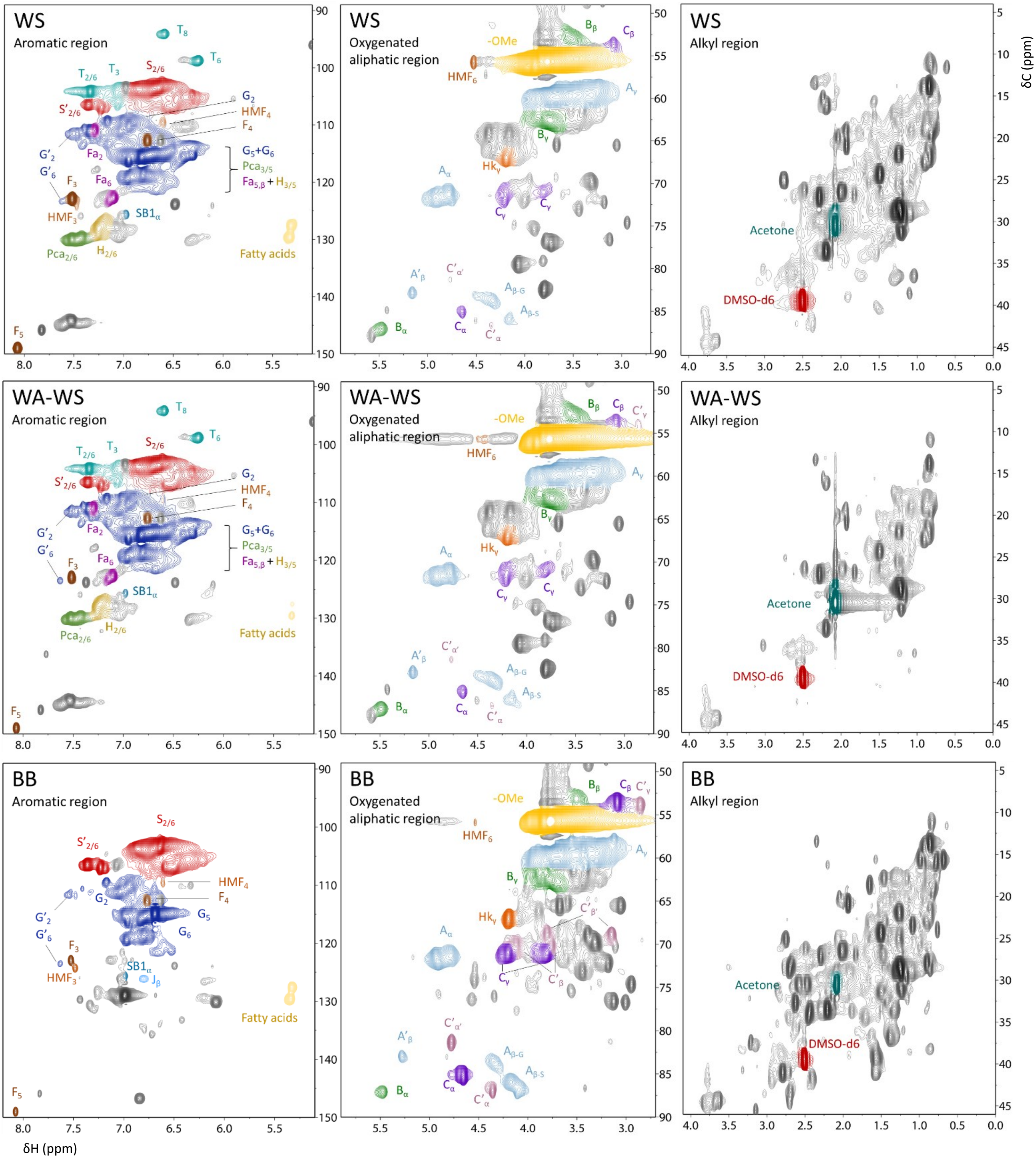
WS
Aromatic region
WS
Oxygenated aliphatic region
WS
Alkyl region
WA-WS
Aromatic region
WA-WS
Oxygenated aliphatic region
WA-WS
Alkyl region
BB
Aromatic region
BB
Oxygenated aliphatic region
BB
Alkyl region
-OMe
HMF6
Acetone
DMSO-d6
Fatty acids
δC (ppm)
δH (ppm)

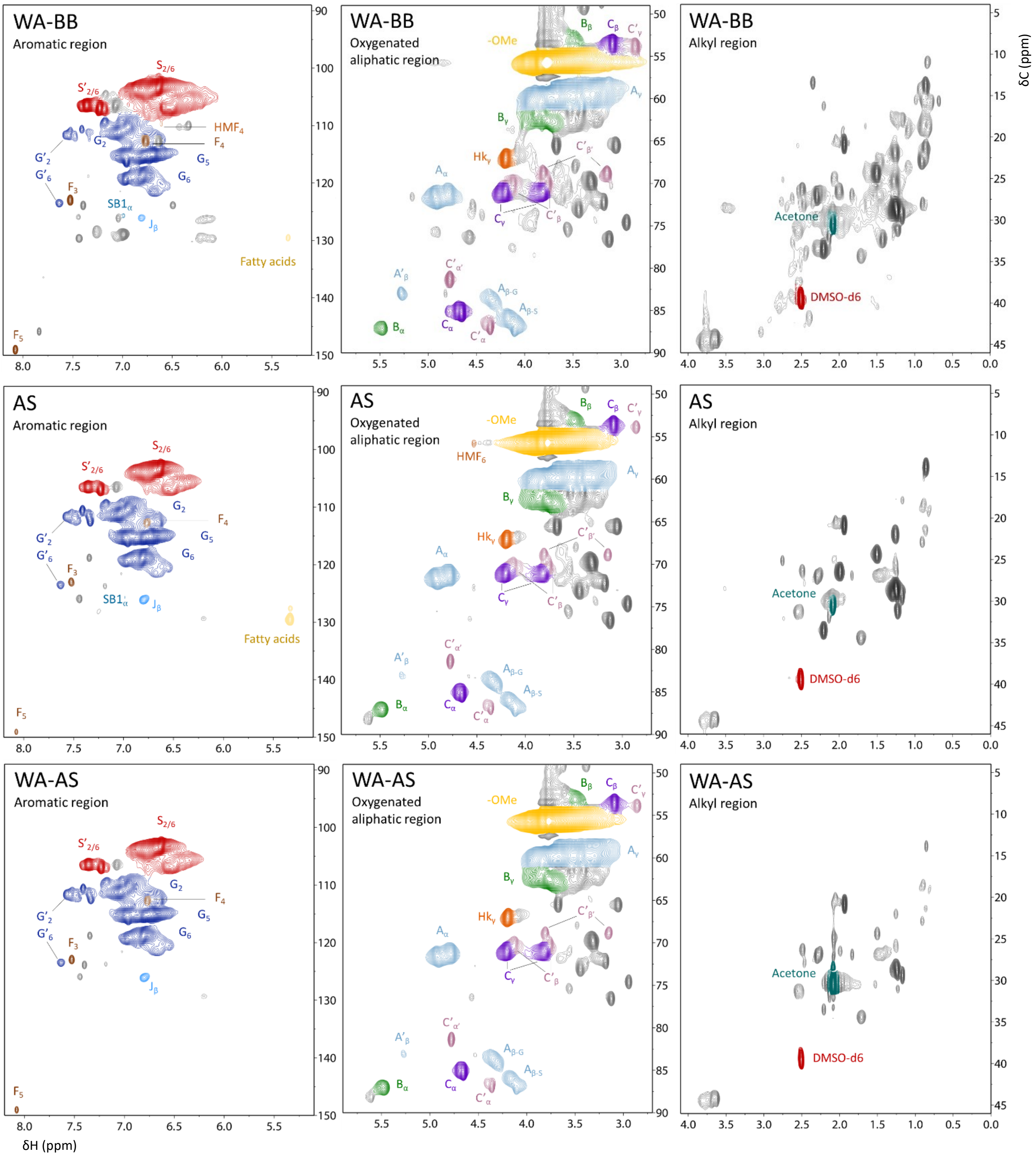
WA-BB
Aromatic region
WA-BB
Oxygenated aliphatic region
WA-BB
Alkyl region
AS
Aromatic region
AS
Oxygenated aliphatic region
AS
Alkyl region
WA-AS
Aromatic region
WA-AS
Oxygenated aliphatic region
WA-AS
Alkyl region
Fatty acids
-OMe
Acetone
DMSO-d6
δH (ppm)
δC (ppm)

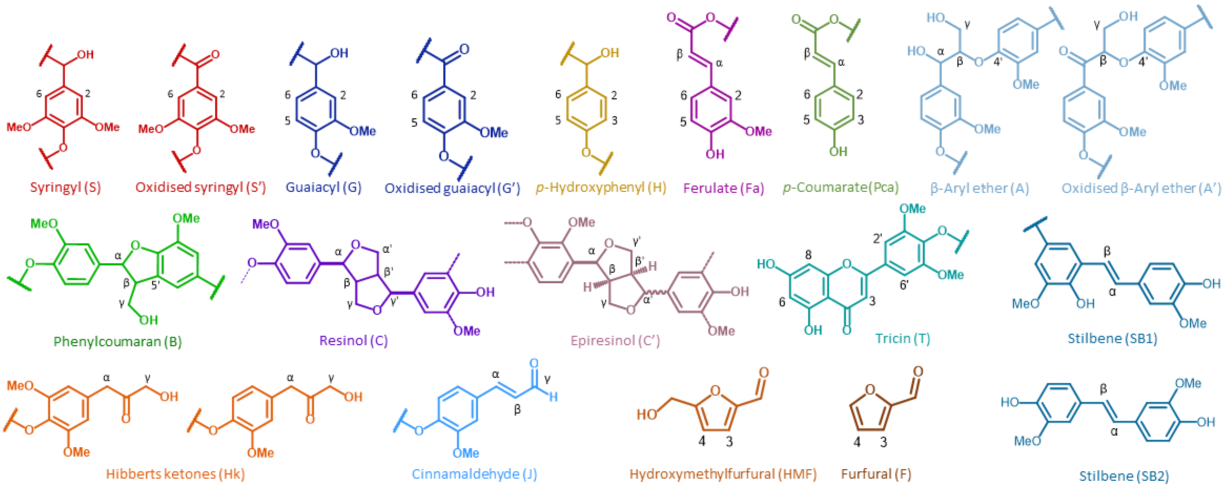

Figure S26 Aromatic/unsaturated region, oxygenated aliphatic side chain region and alkyl region of the 2D HSQC NMR spectra of lignin obtained from fractionation of the untreated and pre-extracted (WA) feedstocks roadside grass (RG), wheat straw (WS), birch branches (BB) and almond shells (AS). WS CEL is wheat straw cellulytic enzymatic lignin. The main identified structures are shown at the bottom.

Lignin G:S:H ratios obtained from Py-GC/MS correlate relatively well with the ratios obtained from 2D HSQC-NMR (Table S26). Overestimation of Py-GC/MS G- lignin units in RG and WS can occur as pyrolysis can also produce 4-vinylguaiacol from ferulates.[32] However, Py-GC/MS of WS and WA-WS lignin found a slightly lower G-unit content. Surprisingly, 4-vinylphenol typically produced from pyrolysis of p-coumarates was not identified and therefore did not contribute to overestimation of H- units in the Py-GC/MS results.

Table S26 Lignin G:S:H ratio as determined by Py-GC/MS and HSQC-NMR.

% Abundance	Py-GC/MS			2D HSQC-NMR		
	G	S	H	G	S	H
RG	71	21	8	60	28	12
WA-RG	67	26	8	61	30	9
WS	61	33	6	56	40	4
WA-WS	60	37	3	55	41	4
BB	41	56	3	31	66	3
WA-BB	44	55	1	32	67	1
AS	61	39	1	51	48	1
WA-AS	58	41	0	51	49	1

Solvent stability

Experimental

Analysis of acetone self-condensation products in organosolv liquors was done using a single analysis on a Thermo Scientific DSQII Series Single Quadrupole GC/MS. After split ratio injection, components were separated with a 30-m Phenomenex Zebron ZB-WAXplus fused silica capillary column (0.25 mm i.d. and 0.25 µm film thickness). The oven was programmed to start at 40 °C for 5 min, ramped to 245 °C at 10°C per minute, and then held for 20 min. Data were collected with Thermo Xcalibur / QuanLab Forms software. The MS was operated in the full scan mode at a scan rate of 5 scans per second.

Results

Direct condensation of acetone (or self-condensation products) to sugars and lignin is not observed in the Py-GC/MS and NMR analysis. GC/MS screening of hemicellulose hydrolysates and lignin Py-GC/MS analysis did show limited formation of furfural acetone. Direct quantification of acetone losses was explored using an autoclave fitted with a cooled sample loop and HPLC analysis of acetone concentrations in the samples. Corrections for autoclave headspace acetone-water vapor composition was applied for determination of acetone losses. However, the experimental approach lacked the sensitivity to accurately determine solvent losses in the range 0-2% especially during biomass fractionation. As a result, only DAA and MO quantification was conducted to assess acetone self-condensation during biomass fractionation. DAA/MO concentrations were determined in the process liquors of untreated and pre-extracted biomass to assess whether pre-extraction would affect acetone self-condensation kinetics. From the results in Table S27 a minor increase in acetone loss is observed after fractionation of pre-extracted feedstocks. One outlier is RG where very low DAA/MO concentrations were found. Due to the partly unknown structure of lignin, the heterogeneity of biomass components and the numerous possibilities for acetone-biomass component condensation routes it is impossible to fully map potential or actual chemical acetone losses. However, analysis of sugar hydrolysates and lignin samples did not reveal any significant presence of acetone-biomass condensates.

Table S27 Diacetone alcohol and mesityloxide concentrations in fractionation liquors

	pH liquor (-)	DAA (g/kg liquor)	MO (g/kg liquor)	Acetone loss to DAA+MO (% w/w)
RG	1.8	0.06	0.06	0.03
WA-RG	1.9	0.71	1.09	0.42
WS	1.8	0.99	1.28	0.54
WA-WS	1.8	1.01	1.55	0.57
BB	1.8	1.02	1.43	0.64
WA-BB	1.8	1.05	1.68	0.71
AS	1.8	0.66	1.07	0.53
WA-AS	1.8	0.78	1.33	0.64

References

1. Sluiter, A.; Ruiz, R.; Scarlata, C.; Sluiter, J.; Templeton, D., Determination of extractives in biomass. *Laboratory Analytical Procedure (LAP)* **2005,** *1617*.
2. Smit, A.; Huijgen, W., Effective fractionation of lignocellulose in herbaceous biomass and hardwood using a mild acetone organosolv process. *Green Chem.* **2017,** *19* (22), 5505-5514. DOI:10.1039/c7gc02379k
3. Sluiter, A.; Hames, B.; Ruiz, R.; Scarlata, C.; Sluiter, J.; Templeton, D., Determination of ash in biomass. *National Renewable Energy Laboratory* **2008,** (NREL/TP-510-42622).
4. Hames, B.; Scarlata, C.; Sluiter, A., Determination of protein content in biomass. *National Renewable Energy Laboratory* **2008**, 1-5.
5. Sun, R. C.; Sun, X., Identification and quantitation of lipophilic extractives from wheat straw. *Ind. Crops Prod.* **2001,** *14* (1), 51-64. DOI:10.1016/s0926-6690(00)00088-1
6. Dewhurst, R. J.; Scollan, N. D.; Youell, S. J.; Tweed, J. K.; Humphreys, M. O., Influence of species, cutting date and cutting interval on the fatty acid composition of grasses. *Grass Forage Sci.* **2001,** *56* (1), 68-74.
7. Queirós, C. S.; Cardoso, S.; Lourenço, A.; Ferreira, J.; Miranda, I.; Lourenço, M. J. V.; Pereira, H., Characterization of walnut, almond, and pine nut shells regarding chemical composition and extract composition. *Biomass Convers Biorefin.* **2020,** *10* (1), 175-188. DOI:10.1007/s13399-019-00424-2

8. Singh, R. D.; Nadar, C. G.; Muir, J.; Arora, A., Green and clean process to obtain low degree of polymerisation xylooligosaccharides from almond shell. *J. Clean. Prod.* **2019,** *241*, 118237. DOI:10.1016/j.jclepro.2019.118237
9. 14429, C. T., Characterization of Waste–Leaching Behaviour Tests–Influence of pH on Leaching with Initial Acid/base Addition. European Committee for Standardization (CEN): 2005.
10. Chen, S. F.; Mowery, R. A.; Castleberry, V. A.; van Walsum, G. P.; Chambliss, C. K., High-performance liquid chromatography method for simultaneous determination of aliphatic acid, aromatic acid and neutral degradation products in biomass pretreatment hydrolysates. *J. Chromatogr. A.* **2006,** *1104* (1-2), 54-61. DOI:10.1016/j.chroma.2005.11.136
11. Rasmussen, H.; Tanner, D.; Sørensen, H. R.; Meyer, A. S., New degradation compounds from lignocellulosic biomass pretreatment: routes for formation of potent oligophenolic enzyme inhibitors. *Green Chem.* **2017,** *19* (2), 464-473. DOI:10.1039/c6gc01809b
12. Shinde, S. D.; Meng, X.; Kumar, R.; Ragauskas, A. J., Recent advances in understanding the pseudo-lignin formation in a lignocellulosic biorefinery. *Green Chem.* **2018,** *20* (10), 2192-2205. DOI:10.1039/c8gc00353j
13. Rasmussen, H.; Sørensen, H. R.; Meyer, A. S., Formation of degradation compounds from lignocellulosic biomass in the biorefinery: sugar reaction mechanisms. *Carbohydr. Res.* **2014,** *385*, 45-57. DOI:10.1016/j.carres.2013.08.029
14. Danon, B.; Marcotullio, G.; de Jong, W., Mechanistic and kinetic aspects of pentose dehydration towards furfural in aqueous media employing homogeneous catalysis. *Green Chem.* **2014,** *16* (1), 39-54. DOI:10.1039/c3gc41351a
15. Nguyen, T. Y.; Cai, C. M.; Kumar, R.; Wyman, C. E., Co-solvent pretreatment reduces costly enzyme requirements for high sugar and ethanol yields from lignocellulosic biomass. *ChemSusChem* **2015,** *8* (10), 1716-1725. DOI:10.1002/cssc.201403045
16. Huijgen, W.; Telysheva, G.; Arshanitsa, A.; Gosselink, R.; De Wild, P., Characteristics of wheat straw lignins from ethanol-based organosolv treatment. *Ind. Crops Prod.* **2014,** *59*, 85-95. DOI:10.1016/j.indcrop.2014.05.003
17. Meng, X.; Ragauskas, A. J., Pseudo-lignin formation during dilute acid pretreatment for cellulosic ethanol. *Recent Adv. Petrochem. Sci.* **2017,** *1* (1). DOI:10.19080/RAPSCI.2017.01.555551
18. Choudhary, V.; Sandler, S. I.; Vlachos, D. G., Conversion of xylose to furfural using Lewis and Brønsted acid catalysts in aqueous media. *Acs Catalysis* **2012,** *2* (9), 2022-2028. DOI:10.1021/cs300265d
19. Bauer, A.; Lizasoain, J.; Theuretzbacher, F.; Agger, J. W.; Rincón, M.; Menardo, S.; Saylor, M. K.; Enguídanos, R.; Nielsen, P. J.; Potthast, A., Steam explosion pretreatment for enhancing biogas production of late harvested hay. *Bioresour. Technol.* **2014,** *166*, 403-410. DOI:10.1016/j.biortech.2014.05.025
20. Costa, C. A. E.; Pinto, P. C. R.; Rodrigues, A. E., Evaluation of chemical processing impact on E. globulus wood lignin and comparison with bark lignin. *Ind. Crops Prod.* **2014,** *61*, 479-491. DOI:10.1016/j.indcrop.2014.07.045
21. Dou, J.; Kim, H.; Li, Y.; Padmakshan, D.; Yue, F.; Ralph, J.; Vuorinen, T., Structural characterization of lignins from willow bark and wood. *J. Agric. Food Chem.* **2018,** *66* (28), 7294-7300. DOI:10.1021/acs.jafc.8b02014
22. Palmqvist, E.; Hahn-Hägerdal, B., Fermentation of lignocellulosic hydrolysates. I: inhibition and detoxification. *Bioresour. Technol.* **2000,** *74* (1), 17-24. DOI:10.1016/S0960-8524(99)00160-1
23. Palmqvist, E.; Hahn-Hägerdal, B., Fermentation of lignocellulosic hydrolysates. II: inhibitors and mechanisms of inhibition. *Bioresour. Technol.* **2000,** *74* (1), 25-33. DOI:10.1016/S0960-8524(99)00161-3
24. Bikova, T.; Treimanis, A.; Rossinska, G.; Telysheva, G., On-line study of lignin behaviour in dilute alkaline solution by the SEC-UV method. *Holzforschung* **2004,** *58* (5), 489-494. DOI:10.1515/HF.2004.074
25. Buranov, A. U.; Mazza, G., Lignin in straw of herbaceous crops. *Ind. Crops Prod.* **2008,** *28* (3), 237-259. DOI:10.1016/j.indcrop.2008.03.008
26. Constant, S.; Wienk, H. L. J.; Frissen, A. E.; Peinder, P. d.; Boelens, R.; van Es, D. S.; Grisel, R. J. H.; Weckhuysen, B. M.; Huijgen, W. J. J.; Gosselink, R. J. A.; Bruijnincx, P. C. A., New insights into the structure and composition of technical lignins: a comparative characterisation study. *Green Chem.* **2016,** *18* (9), 2651-2665. DOI:10.1039/c5gc03043a
27. Kim, H.; Padmakshan, D.; Li, Y.; Rencoret, J.; Hatfield, R. D.; Ralph, J., Characterization and elimination of undesirable protein residues in plant cell wall materials for enhancing lignin analysis by solution-state nuclear magnetic resonance spectroscopy. *Biomacromolecules* **2017,** *18* (12), 4184-4195. DOI:10.1021/acs.biomac.7b01223
28. Balakshin, M.; Capanema, E.; Gracz, H.; Chang, H.-m.; Jameel, H., Quantification of lignin–carbohydrate linkages with high-resolution NMR spectroscopy. *Planta* **2011,** *233* (6), 1097-1110. DOI:10.1007/s00425-011-1359-2
29. Lagerquist, L.; Pranovich, A.; Smeds, A.; von Schoultz, S.; Vähäsalo, L.; Rahkila, J.; Kilpeläinen, I.; Tamminen, T.; Willför, S.; Eklund, P., Structural characterization of birch lignin isolated from a pressurized hot water extraction and mild alkali pulped biorefinery process. *Ind. Crops Prod.* **2018,** *111*, 306-316. DOI:10.1016/j.indcrop.2017.10.040
30. Rencoret, J.; José, C.; Gutiérrez, A.; Martínez, Á. T.; Li, S.; Parkås, J.; Lundquist, K., Origin of the acetylated structures present in white birch (Betula pendula Roth) milled wood lignin. *Wood Sci. Technol.* **2012,** *46* (1), 459-471. DOI:10.1007/s00226-011-0417-z
31. Paulsen Thoresen, P.; Lange, H.; Crestini, C.; Rova, U.; Matsakas, L.; Christakopoulos, P., Characterization of Organosolv Birch Lignins: Toward Application-Specific Lignin Production. *ACS Omega* **2021,** *6* (6), 4374-4385. DOI:10.1021/acsomega.0c05719
32. Del Río, J. C.; Rencoret, J.; Prinsen, P.; Martínez, A. n. T.; Ralph, J.; Gutiérrez, A., Structural characterization of wheat straw lignin as revealed by analytical pyrolysis, 2D-NMR, and reductive cleavage methods. *J. Agric. Food Chem.* **2012,** *60* (23), 5922-5935. DOI:10.1021/jf301002n
